# Pregnancy-associated systemic gene expression compared to a pre-pregnancy baseline, among healthy women with term pregnancies

**DOI:** 10.3389/fimmu.2023.1161084

**Published:** 2023-06-05

**Authors:** Matthew L. Wright, Dana E. Goin, Mette Kiel Smed, Nicholas P. Jewell, J. Lee Nelson, Jørn Olsen, Merete Lund Hetland, Vibeke Zoffmann, Damini Jawaheer

**Affiliations:** ^1^ Children’s Hospital Oakland Research Institute, Oakland, CA, United States; ^2^ Northwestern University, Feinberg School of Medicine, Chicago, IL, United States; ^3^ Department of Obstetrics, Gynecology, and Reproductive Sciences, University of California, San Francisco, San Francisco, CA, United States; ^4^ Juliane Marie Centeret, Rigshospitalet, Copenhagen, Denmark; ^5^ Department of Medical Statistics, London School of Hygiene and Tropical Medicine, London, United Kingdom; ^6^ Clinical Research Division, Fred Hutchinson Cancer Research Center, Seattle, WA, United States; ^7^ Department of Medicine, University of Washington, Seattle, WA, United States; ^8^ Department of Epidemiology, University of California, Los Angeles, Los Angeles, CA, United States; ^9^ Department of Clinical Epidemiology, Aarhus University Hospital, Aarhus, Denmark; ^10^ DANBIO Registry and Copenhagen Centre for Arthritis Research, Centre for Rheumatology and Spine Diseases, Rigshospitalet, Glostrup, Denmark; ^11^ Faculty of Health and Medical Sciences, University of Copenhagen, Copenhagen, Denmark

**Keywords:** pregnancy, gene expression, RNA sequencing (RNAseq), long non-coding (lnc) RNA, healthy women

## Abstract

**Background:**

Pregnancy is known to induce extensive biological changes in the healthy mother. Little is known, however, about what these changes are at the molecular level. We have examined systemic expression changes in protein-coding genes and long non-coding (lnc) RNAs during and after pregnancy, compared to before pregnancy, among healthy women with term pregnancies.

**Methods:**

Blood samples were collected from 14 healthy women enrolled in our prospective pregnancy cohort at 7 time-points (before, during and after pregnancy). Total RNA from frozen whole blood was used for RNA sequencing. Following raw read alignment and assembly, gene-level counts were obtained for protein-coding genes and long non-coding RNAs. At each time-point, cell type proportions were estimated using deconvolution. To examine associations between pregnancy status and gene expression over time, Generalized Estimating Equation (GEE) models were fitted, adjusting for age at conception, and with and without adjusting for changes in cell type proportions. Fold-changes in expression at each trimester were examined relative to the pre-pregnancy baseline.

**Results:**

Numerous immune-related genes demonstrated pregnancy-associated expression, in a time-dependent manner. The genes that demonstrated the largest changes in expression included several that were neutrophil-related (over-expressed) and numerous immunoglobulin genes (under-expressed). Estimated cell proportions revealed a marked increase in neutrophils, and less so of activated CD4 memory T cells, during pregnancy, while most other cell type proportions decreased or remained unchanged. Adjusting for cell type proportions in our model revealed that although most of the expression changes were due to changes in cell type proportions in the bloodstream, transcriptional regulation was also involved, especially in down-regulating expression of type I interferon inducible genes.

**Conclusion:**

Compared to a pre-pregnancy baseline, there were extensive systemic changes in cell type proportions, gene expression and biological pathways associated with different stages of pregnancy and postpartum among healthy women. Some were due to changes in cell type proportions and some due to gene regulation. In addition to providing insight into term pregnancy among healthy women, these findings also provide a “normal” reference for abnormal pregnancies and for autoimmune diseases that improve or worsen during pregnancy, to assess deviations from normal.

## Introduction

Human pregnancy represents a period during which an intricate series of changes occur in the mother at multiple levels, leading to the remarkable development of a fertilized ovum into a small human being. In addition to its crucial role in protecting and supporting growth of the fetus to term, pregnancy also appears to have other unexpected benefits; for example, it can induce a natural improvement of some incurable diseases such as rheumatoid arthritis (RA) and multiple sclerosis, and the incidence of these diseases appears to be significantly reduced during pregnancy (1–3). While much effort has been devoted over the years to understand the influence of pregnancy immunology on fetal survival (4–6), little is known about systemic biological changes that pregnancy induces in the mother at the molecular level in health or in disease. Such data about maternal changes that occur during the course of pregnancy among healthy women with term pregnancies could represent a “normal baseline” to which data from abnormal or disease pregnancies could be compared to assess deviations from normal.

As we previously demonstrated (7), monitoring systemic gene expression over time as pregnancy progresses is one way to assess pregnancy-induced physiologic changes. Only a few other studies have examined gene expression in the circulation of healthy women during pregnancy (8–13), and of those, even fewer have focused on all trimesters of pregnancy (8, 12). Importantly, none of these studies included a pre-pregnancy baseline, which is crucial to determine what changes are brought about by pregnancy. Instead, studies used data from unrelated non-pregnant women to represent the “pre-pregnancy” period, although this introduces heterogeneity in the data due to between-person variation, or late pregnancy data were compared to those from early pregnancy.

In our previous study (7), we reported gene expression changes observed in each trimester of pregnancy, compared to a pre-pregnancy baseline, in a combined group of 5 healthy women and 20 women with RA. We have now built on those previous findings by examining longitudinal gene expression patterns among 14 healthy women using RNA sequencing (RNA-seq) data from pre-pregnancy, through each trimester, until 3 months postpartum. We have tested the hypothesis that pregnancy-induced gene expression changes observed in the maternal periphery are due to a combination of changes in different cell proportions in the bloodstream and gene regulation. Further, given the emerging role of long non-coding (lnc) RNAs as regulators of gene expression in numerous biological processes including pregnancy (14–16), we have examined lncRNA expression patterns as well as the co-expression between coding genes with pregnancy-associated expression and lncRNAs that may be regulating their expression epigenetically.

## Subjects and methods

### Study subjects

Healthy women of Danish descent who were planning a pregnancy were enrolled in our pregnancy cohort in Denmark, and were prospectively followed, as previously described (7, 17). Women who had a history of autoimmune diseases and/or who were on fertility treatment were excluded from the cohort. A set of 14 healthy women from this cohort were included in the present study. The study was approved by the Ethics Committee for Region Hovedstaden (Denmark) (Protocol #: H-2-2009-150), the Danish Data Protection Agency, the Children’s Hospital Oakland Research Institute Institutional Review Board (IRB number: 2009-073), and the Northwestern University IRB (IRB number: STU00217093). All subjects provided written informed consent prior to enrollment.

### Sample collection and processing

Blood samples were drawn into PAXgene tubes at 7 pre-defined time-points before, during (i.e. once each trimester, in gestational weeks 6-8, 24 and 32, respectively) and after pregnancy (3, 6 and 9 months postpartum), and frozen. Because conception is estimated to happen 11-21 days after the first day of the last period, the dates of conception were estimated as 16 days (average) after the first day of the last period. Total RNA was manually extracted from the blood samples using the PAXgene Blood RNA Kit according to the manufacturer’s protocol, and RNA integrity was assayed using a 2100 Bioanalyzer. 250 ng of total RNA were first depleted of ribosomal RNAs and globin mRNAs using the KAPA RiboErase kit (Roche), and KAPA globin depletion hybridizing oligos (Roche), respectively. Barcoded and stranded cDNA libraries were then prepared using the KAPA RNA HyperPrep kit. Pooled libraries were sequenced on an Illumina NovaSeq 6000 instrument, targeting an average of 100 million 150 bp paired-end reads.

### Bioinformatics processing

Rigorous quality control (QC) of the raw data was performed using FASTQC (18), Picard (19) and HTSTREAM (20). Raw FASTQ reads were trimmed using Cutadapt (v2.4) (21) and aligned to the human genome (GRCh38; Ensembl v98) using HISAT2 (v 2.1.0) (22). Multi-mapped reads were filtered using Samtools (23, 24). Aligned reads were assembled into transcripts and merged using StringTie (v 2.1.1) (25).

Novel lncRNAs in our assembled transcripts were assessed by removing: 1) transcripts overlapping any known transcript on the same strand (Bedtools v 2.28.0); 2) transcripts with open reading frames (ORFs) > 100 amino acids (TransDecoder v 5.5.0); 3) any transcripts that, when translated, had similarity to known proteins/protein domains [blastx hits to the RefSeq Protein or Pfam databases] (Blast+ v 2.7.1, flags: -strand plus -max_target_seqs 1 -evalue 1e-5); 4) any transcripts classified as coding by at least one of 3 tools for detecting coding potential [CPAT (v 3.0.2) (26), CPC (v 2.0) (27), and FEELnc (v 0.2) (28)]; 5) single-exon transcripts. Remaining transcripts were classified as “novel lncRNAs” and appended to the Ensembl v98 gtf file. The resulting annotation file (gtf) containing all known and newly discovered (from our data) transcripts was used as reference to obtain gene level counts for all known genes and lncRNAs as well as novel lncRNAs with featureCounts (v 2.0.0, flags: -s 2 -p) (29).

Raw counts were loaded into R and “rRNA” and “pseudogene” gene types were removed, along with gene types “misc_RNA”, “Mt_tRNA”, “scaRNA”, “snRNA”, “snoRNA”, and “TEC”. Genes with low expression were removed by keeping only genes with CPM > 10/L in 8 or more samples, where L is the minimum library size in millions. Library size was normalized in edgeR (v 3.30.3) (30) with the trimmed mean of M-values (TMM) method using the calcNormFactors function, and exported normalized counts for downstream statistical analyses.

### Statistical analysis

#### Deconvolution of bulk RNA-seq data

To estimate cell type proportions in the samples from different time-points, raw reads were aligned to the Ensembl v98 transcriptome using kallisto (v 0.46.1) and aggregated to gene-level data using tximport (v 1.18) in R. Gene-level data for each sample were deconvolved using CIBERSORTx and the accompanying LM22 signature matrix that is based on 22 human immune cell types (31). We used Principal Components Analysis (PCA) to condense the information about changes in all 22 estimated cell type proportions into principal components (PCs) as proposed by Kong et al. (32), and tested them for association with gene expression. The PCs significantly associated with gene expression were included as covariates in the regression models described below.

#### Longitudinal regression model

To examine associations between pregnancy status (7 time-points: pre-pregnancy, each trimester, and every 3 months after childbirth until 9 months postpartum) and gene expression levels over those time points, Generalized Estimating Equation (GEE) models were fitted, using normalized gene counts as the outcome variable with repeated measures and pregnancy status as the main explanatory (factor) variable, employing robust standard error estimation (STATA v14.2). Age at conception was adjusted for in the model. A negative binomial link function was used to handle over-dispersion in RNA-seq gene counts. Both independent and exchangeable correlation structures were considered. The Benjamini-Hochberg False Discovery Rate (FDR) method was used to correct for multiple testing. A threshold of FDR<0.05 was used to assess statistical significance. In order to assess whether observed changes in gene expression over time were due to changes in cell proportions or to those genes being regulated, we repeated the analyses, adjusting for significant PCs that were associated with gene expression. To address whether parity influenced the gene expression results, we re-ran the GEE models, adjusting for parity (0, 1, 2).

#### Temporal changes in gene expression

For genes demonstrating a significant association with pregnancy status in the GEE models, fold-changes in expression levels at each trimester were examined relative to the T0 baseline using differential gene expression analysis in edgeR. Changes in cell type proportions were also adjusted for in edgeR when assessing expression changes for genes significant in the adjusted GEE model.

#### Functional enrichment

Enrichment of GO terms, and KEGG and Reactome pathways were assessed using WebgestaltR (33). Interactions between proteins encoded by the significant genes were based on data from the STRING database (34) and visualized in Cytoscape (v3.8.1) (35).

#### Transcription factor analysis

Enrichment of transcription factor targets was performed using the *fora* function in the fgsea R package (36). Transcription factor-target regulons were pulled from the DoRothEA database, using confidence levels A, B, and C (37, 38).

#### Co-expression network analysis

In order to determine whether lncRNAs could be involved in regulating the expression of genes that exhibited expression changes due to transcriptional regulation (significant in GEE model adjusted for cell type proportions), co-expression of those genes and lncRNAs was examined. Co-expression analysis of normalized gene-level counts was performed in R using the Weighted Gene Co-expression Network Analysis (WGCNA) package (v1.69) (39) with power=5, networkType=signedHybrid, corType=bicor, maxPOutliers=0.1, and mergeCutHeight=0.25. Further, since genes co-expressed within a module tend to be co-regulated and functionally related, functional analysis of the modules that included both protein-coding genes and lncRNAs was performed to gain insight into potential functions of the lncRNAs being co-expressed with specific genes within different modules. Enrichment of pregnancy-associated genes within co-expression modules was assessed by hypergeometric test.

## Results

### Study subjects

Of the 14 women included in the analyses, 7 were nulliparous, 6 had had one live birth and 1 had had two live births. Data were available from pre-pregnancy (T0) to 9 months postpartum for 8 women, and up to 3^rd^ trimester (T3) for the other 6, one of whom was also missing T0 data. The average age at conception was 28.9 ± 1.0 years. The average length of gestation was 40.3 ± 0.3 weeks. The average time between the pre-pregnancy visits and the estimated conception dates was 83 ± 27 days.

### Data

After rigorous QC, the gene expression (RNA-seq) data were visualized on a PCA plot (**Figure S1**). The data clustered by time-point, and clusters/time-points with similar gene expression patterns were found to overlap. All results are presented henceforth up to the 3 months postpartum (PP3) time-point.

### Changes in cell type proportions with advancing pregnancy

**Figure 1:**
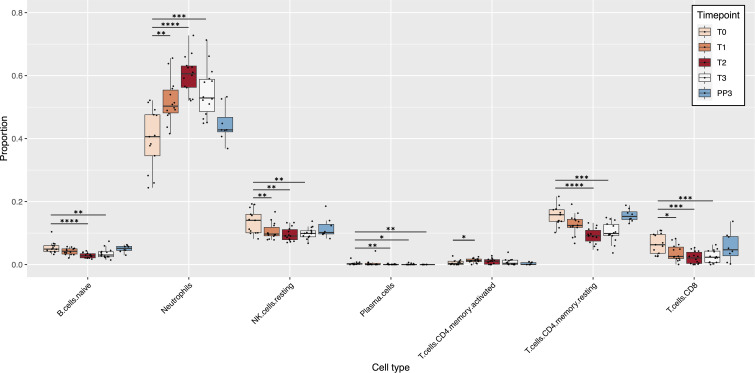
Pregnancy-induced changes in immune cell populations. The box plots show changes in the estimated relative proportions of cell types in blood samples from T0 to PP3. These relative proportions were estimated from bulk RNA-seq data using CIBERSORTx. Only cell types that demonstrated significant changes in relative proportions (at one or more time-point) compared to the T0 baseline are shown. p values for significant changes in proportions from the T0 baseline were as follows: * p<0.05; ** p<0.01; *** p<0.001; **** p<0.0001. [T0: Pre-pregnancy; T1, 1^st^ trimester; T2, 2^nd^ trimester; T3, 3^rd^ trimester; PP3, 3 months postpartum].

Deconvolution of the RNA-seq data using the LM22 reference produced relative cell type proportions at each time point for the 22 immune cell types present in the LM22 reference. Of those 22 cell types, 7 showed significant changes in their proportions during pregnancy compared to T0 (**Figure 1**). Among the cell types that showed significant changes in their proportions during pregnancy, only neutrophils and activated CD4 memory T cells showed an increase in proportions compared to pre-pregnancy, both increasing at the start of pregnancy and then decreasing slightly at T3 and PP3. The cell populations that showed a significant decrease in proportions with advancing pregnancy relative to T0 included naïve B cells, resting CD4 memory T cells, CD8 T cells, resting NK cells, and plasma cells. Cell proportions decreased up to 2^nd^ trimester (T2) and then increased again until PP3. There were no significant changes observed for memory B cells, dendritic cells (resting and activated), eosinophils, macrophages (M0, M1 and M2), mast cells (resting and activated), monocytes, NK cells (activated), CD4 naïve T cells, regulatory T cells, follicular helper T cells and gamma delta T cells (**Figure S2**). Among the 8 women who had postpartum data available, there were no significant changes compared to the T0 baseline, except for plasma cells which were at a lower proportion at PP3 (median: 0% vs 0.002%; p=0.001).

### Protein-coding genes

#### Changes in gene expression during pregnancy

**Figure 2:**
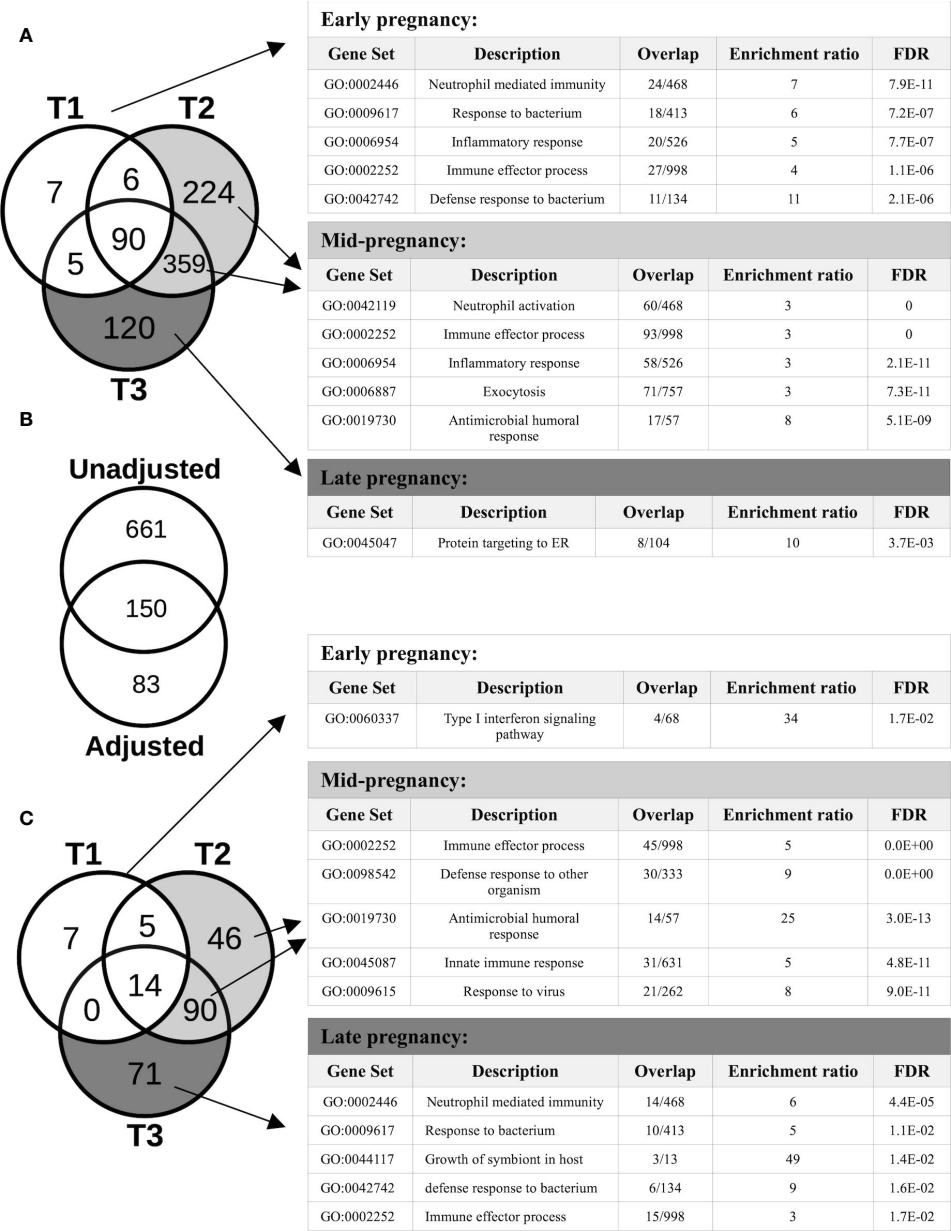
Protein-coding genes with pregnancy-associated expression. **(A)** The Venn diagram shows the number of protein-coding genes with pregnancy-associated expression (fold-change, FC≥2, FDR<0.05) in each trimester (vs the pre-pregnancy baseline) in the unadjusted analysis. The Gene ontology (GO) biological processes shown in the tables are enriched in genes that started demonstrating pregnancy-associated expression at different stages of pregnancy. **(B)** Of the 811 genes showing pregnancy-associated expression in the unadjusted model, 150 remained significant after adjusting for changes in cell type proportions, and another 83 became newly significant in the adjusted model. **(C)** After adjusting for changes in cell type proportions in the GEE model, the number of genes demonstrating pregnancy-associated expression that was due to gene regulation became apparent. The GO biological processes shown are enriched in genes that started demonstrating pregnancy-associated expression at different stages of pregnancy.

Overall, 9,266 genes demonstrated pregnancy-associated expression (FDR<0.05) in the Generalized Estimating Equations (GEE) model unadjusted for cell type proportions, irrespective of fold-changes in expression. After applying our fold-change (FC) cut-off for significance (FC>=2), 811 genes exhibited expression patterns that were significantly associated (FDR<0.05; FC>=2) with different stages of pregnancy, compared to the T0 baseline. As shown in the Venn diagram of **Figure 2A**, different sets of genes showed significant changes in expression at different stages of pregnancy. In particular, for those that were the most over-expressed by the 3^rd^ trimester, the changes appeared early on in pregnancy, and persisted through pregnancy, becoming more prominent by mid- or late pregnancy. Genes whose expression were significantly associated with pregnancy in the 1^st^ trimester (T1) were either 2-8-fold over-expressed (such as MMP8/9, CD177, DEFA1, CRISP3, CAMP, ORM1, S100A8/9/12, S100P, CA4, PGLYRP1, ARG1 and TLR5), or 2-4-fold under-expressed (such as IL34, IDO1 and several immunoglobulin heavy and light chain variable genes). Most of these genes became even more over- or under-expressed by T2 and they remained so at T3. Among the genes whose expression patterns were significantly associated with pregnancy at or from T2 were IL1B, IL1R1/2, IL4R, CD55, CXCL1/6, CXCR1, CEACAM3/6/8, ELANE, FOXQ1, IFNGR1, SERPINA1/B1/B10, DEFA3/4, IL18RAP, NLRC4, NLRP6, TLR1/4/6/8, PADI2/4, MMP1/25, OLFM4, FOXQ1 and numerous genes from the solute carrier (SLC) family (2-15-fold over-expressed), together with GATA2, IFI44, IFI44L, OAS1/2/3, IL4, NLRP7 and numerous immunoglobulin genes (2-7-fold under-expressed). Genes whose expression were significantly modulated only in late pregnancy (T3) included CD24, complement genes C1QA/B/C and several genes encoding ribosomal proteins (2-4-fold over-expressed), and GATA6, FGFR2, IL5RA, SOX5 and some immunoglobulin variable genes (2-12-fold under-expressed). The Gene Ontology (GO) terms that were enriched in the genes that started to show pregnancy-associated expression at each trimester are shown in **Figure 2A**. A comprehensive list of genes with pregnancy-associated expression at each trimester is provided in **Table S1**.

**Figure 3:**
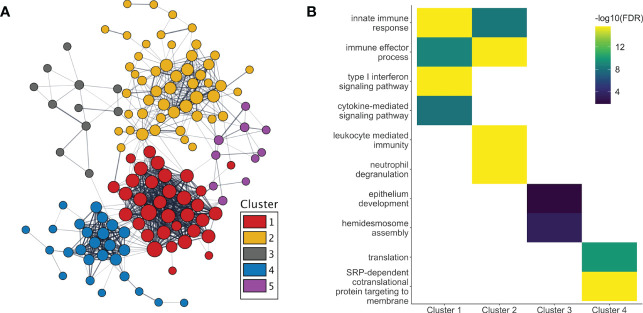
Protein interactions and functional enrichment of genes whose expression are regulated during pregnancy. **(A)** The protein products of the genes with pregnancy-associated expression in the adjusted analyses were part of a common protein-protein interaction network. Five distinct clusters were observed within the network. **(B)** The GO terms that were the most significantly enriched in genes from clusters 1-4 are shown. No GO term enrichment was found for genes in cluster 5. [T1, 1^st^ trimester; T2, 2^nd^ trimester; T3, 3^rd^ trimester].

After adjusting for changes in cell type proportions in the GEE model, 233 genes were found to be significantly associated with pregnancy. Three different patterns of expression were apparent (**Figure 2B**). First, the majority (661 of 811, i.e. 82%) of genes with pregnancy-associated expression in the unadjusted model (such as IL1B, IL1R1/R2, IL27, MMP1/9/25, PADI2/4, TLR1/4/5/6/8 and numerous immunoglobulin genes) were no longer significant in the adjusted model. Thus, most of the pregnancy-induced changes in expression appeared to be due to changes in cell type proportions as pregnancy progressed over time. Second, some genes remained significant in the adjusted model (n=150; for example, OLFM4, CD24, CD177, CAMP, CRISP3, IDO1, GATA2, ELANE, IL34, IL4, DEFA1/3/4, IFI44/44L, CEACAM6/8, OAS1/2/3, MMP8, S100P, S100A8/9/12, RPL7/23/34/39/41), indicating that changes in their expression were due to a combination of changes in cell proportions and transcriptional regulation. For a third group of genes (n=83), their pregnancy-associated expression only became apparent after adjusting for cell type proportions, which suggests that changes in their expression during pregnancy was most likely due to transcriptional regulation. These included the type I Interferon inducible genes HERC5/6, OASL, IFI6, IFIH1, IFIT1/3, MX1, CMPK2, SIGLEC1 and STAT2 (down-regulated), and some genes encoding ribosomal proteins such as RPL9/31, RPS7/18/21 (up-regulated), among others. As in the unadjusted model, different sets of genes showed significant changes in expression at different stages of pregnancy, and different GO terms enriched in those genes (**Figure 2C**). Of the proteins encoded by genes whose pregnancy-associated expression was at least in part under transcriptional regulation, 56% (131 of 233) belonged to a common interaction network which included 5 tightly interacting clusters (**Figure 3A**). The GO terms that were enriched in the genes within these sub-clusters are shown in **Figure 3B**. The list of genes with significant pregnancy-associated expression in the adjusted model is available as **Table S2**.

Overall, when we combined the results from the two GEE models unadjusted or adjusted for cell type proportions, we found that a large number of genes (n=12,694) demonstrated significant pregnancy-associated expression (FDR<0.05) when fold-changes in expression were not included in the criteria for significance. Those were enriched in numerous biological pathways as can be expected during pregnancy (**Table 1**).

**Table 1:** KEGG pathways enriched in genes with pregnancy-associated expression.

		KEGG Pathway	No. of genes identified (No. of genes in pathway)	FDR^*^
Pathways enriched in genes over-expressed during pregnancy:				
Metabolism				
	**Carbohydrate metabolism**			
		Starch and sucrose metabolism	14 (24)	1.0E-02
		Pentose phosphate pathway	12 (22)	4.0E-02
		Galactose metabolism	12 (22)	4.0E-02
	**Energy metabolism**			
		Oxidative phosphorylation	55 (115)	2.0E-04
Genetic Information Processing				
	**Translation**			
		Ribosome	55 (85)	4.0E-09
	**Folding, sorting and degradation**			
		Ubiquitin mediated proteolysis	62 (128)	7.0E-05
Environmental Information Processing				
	**Signal Transduction**			
		MAPK signaling pathway	95 (206)	9.0E-06
		JAK-STAT signaling pathway	49 (102)	5.0E-04
		ErbB signaling pathway	34 (72)	6.0E-03
		VEGF signaling pathway	29 (59)	6.0E-03
		mTOR signaling pathway	22 (44)	1.0E-02
		TGF-beta signaling pathway	28 (64)	4.0E-02
Cellular Processes				
	**Transport and catabolism**			
		Endocytosis	83 (164)	7.0E-07
		Lysosome	59 (116)	3.0E-05
		Regulation of autophagy	11 (19)	3.0E-02
	**Cell growth and death**			
		Apoptosis	40 (80)	7.0E-04
	**Cellular community - eukaryotes**			
		Focal adhesion	63 (154)	8.0E-03
		Adherens junction	29 (63)	2.0E-02
	**Cell motility**			
		Regulation of actin cytoskeleton	76 (161)	3.0E-05
Organismal Systems				
	**Immune system**			
		Fc gamma R-mediated phagocytosis	52 (89)	7.0E-07
		Chemokine signaling pathway	74 (142)	7.0E-07
		Leukocyte transendothelial migration	47 (83)	8.0E-06
		NOD-like receptor signaling pathway	33 (55)	4.0E-05
		Toll-like receptor signaling pathway	45 (84)	5.0E-05
		B cell receptor signaling pathway	38 (70)	1.0E-04
		Fc epsilon RI signaling pathway	35 (64)	2.0E-04
		Complement and coagulation cascades	23 (40)	1.0E-03
		Natural killer cell mediated cytotoxicity	47 (101)	1.0E-03
		T cell receptor signaling pathway	44 (99)	6.0E-03
	**Endocrine system**			
		Insulin signaling pathway	55 (114)	2.0E-04
		Adipocytokine signaling pathway	27 (56)	1.0E-02
		Progesterone-mediated oocyte maturation	33 (73)	1.0E-02
		GnRH signaling pathway	33 (74)	2.0E-02
	**Nervous system**			
		Neurotrophin signaling pathway	58 (109)	7.0E-06
		Long-term potentiation	26 (48)	2.0E-03
Human Diseases				
	**Cancer: overview**			
		Pathways in cancer	109 (260)	1.0E-04
	**Cancer: specific types**			
		Pancreatic cancer	36 (65)	1.0E-04
		Non-small cell lung cancer	29 (49)	2.0E-04
		Chronic myeloid leukemia	36 (67)	2.0E-04
		Renal cell carcinoma	35 (65)	3.0E-04
		Prostate cancer	39 (78)	8.0E-04
		Acute myeloid leukemia	29 (54)	1.0E-03
		Endometrial cancer	25 (47)	3.0E-03
		Glioma	26 (52)	7.0E-03
		Melanoma	24 (49)	1.0E-02
		Colorectal cancer	27 (58)	2.0E-02
		Small cell lung cancer	32 (73)	2.0E-02
	**Infectious disease: bacterial**			
		Epithelial cell signaling in Helicobacter pylori infection	34 (59)	9.0E-05
		Vibrio cholerae infection	24 (43)	2.0E-03
		Pathogenic Escherichia coli infection	24 (47)	7.0E-03
	**Infectious disease: parasitic**			
		Leishmaniasis	33 (66)	2.0E-03
	**Neurodegenerative diseases**			
		Alzheimer disease	69 (144)	4.0E-05
		Huntington disease	71 (156)	2.0E-04
		Parkinson disease	52 (114)	1.0E-03
		Amyotrophic lateral sclerosis	22 (39)	2.0E-03
	**Endocrine and metabolic disease**			
		Type II diabetes mellitus	17 (35)	5.0E-02
Pathways enriched in genes under-expressed during pregnancy:				
Metabolism				
	**Carbohydrate metabolism**			
		Citrate cycle (TCA cycle)	19 (27)	0.03
	**Amino acid metabolism**			
		Valine, leucine and isoleucine degradation	27 (42)	0.03
	**Glycan biosynthesis and metabolism**			
		N-Glycan biosynthesis	29 (44)	0.01
Genetic Information Processing				
	**Translation**			
		Aminoacyl-tRNA biosynthesis	16 (22)	0.03
	**Replication and repair**			
		DNA replication	23 (36)	0.04
Human Diseases				
	**Immune disease**			
		Primary immunodeficiency	24 (31)	0.001

Numerous KEGG pathways were enriched in genes that were significantly associated with pregnancy in the GEE model adjusted for cell type proportions and/or the unadjusted model (without any fold-change cut-off). These have been grouped under broad functional categories. KEGG pathways enriched in genes that were over-expressed are shown separately from those enriched in genes that were under-expressed. (* FDR: FDR-adjusted p-value).

The results did not change when the GEE models were adjusted for parity (Pearson correlation > 0.98).

#### Postpartum changes in expression

Compared to T3, 558 genes showed significant changes in expression at PP3 (FC≥2, FDR<0.05) in the model unadjusted for cell type proportions. Of these, 70% (n=390) were among those whose expression were significantly associated with pregnancy in one or more trimesters. These genes were reverting to pre-pregnancy expression levels as suggested by the inverse correlation between their expression changes from T3 to PP3 and from T0 to T3 (pairwise correlation=-0.96, p<0.00005). The genes showing the largest changes in expression at PP3 (vs T3) included OLFM4, MMP8/9, CD177, DEFA1/3/4, CEACAM6/8, CRISP3, CAMP, FOXQ1, ELANE, S100A8/9, S100P, C1QA/B, among others (2-45-fold under-expressed), and IL4, GATA2/6, IL5RA, IL34, and some T cell receptor genes (2-31-fold over-expressed). After adjusting for cell type proportions, 359 of the 558 genes (64%) were no longer significantly associated with the PP3 time-point while another 17 became newly associated. Compared to T0, 36 genes showed significant changes in expression at PP3 (FC≥2, FDR<0.05) in the unadjusted model, all being under-expressed at PP3 and most (24 of 36, i.e. 67%) being immunoglobulin genes.

#### Transcription factor analysis

Transcription factor (TF) target enrichment analysis revealed that the genes showing pregnancy-associated expression at/from T1 (n=26) were significantly enriched in target genes of STAT2 (CMPK2, EPSTI1, OAS2, OAS3; fold-enrichment=15.5, FDR=0.04) and IRF9 (OAS2, OAS3, RSAD2; fold-enrichment=25.7, FDR=0.04). Genes that became associated with pregnancy at or from T2 (n=136) were significantly enriched in additional target genes of STAT2 (DDX60, DHX58, EIF2AK2, IFI6, IFIH1, IFIT1, IFIT3, ISG15, MOV10, MX1, OAS1, OASL, PARP10, PARP12, PML, PNPT1, RNF213, RTP4, XAF1; fold-enrichment=19.9, FDR=4.6E-17) and IRF9 (IFIT1, IFIT3, ISG15, MX1, PML; fold-enrichment=11.6, FDR=1.4E-2). No significant TF target enrichment was observed among the genes that started showing pregnancy-associated expression only at T3 (n=71) and those with expression associated with PP3. A list of TFs and their target genes that were among those demonstrating pregnancy-associated expression in the adjusted GEE model is provided in **Table S4**, irrespective of whether those target genes were significantly enriched or not.

### lncRNAs

**Figure 4:**
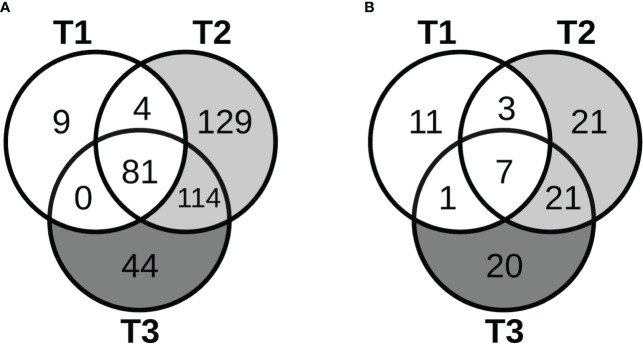
Long non-coding RNAs with pregnancy-associated expression. The Venn diagrams show the number of lncRNAs that started to show pregnancy-associated expression at the different stages of pregnancy in the **(A)** unadjusted analyses, and **(B)** adjusted analyses.

The patterns of expression of lncRNAs during pregnancy, including known and novel ones and a few microRNAs (miRNAs) identified in our data, were similar to those for protein-coding genes. As shown in **Figure 4A**, most of the lncRNAs that demonstrated significant changes in expression in early pregnancy (T1) continued to show significant changes in mid- (T2) and late pregnancy (T3). Furthermore, for the majority of lncRNAs that were the most over-expressed (3- to 24-fold) by T3, the changes were already significant in early pregnancy, persisting through pregnancy, and becoming more prominent by mid- or late pregnancy. After cell type proportions were adjusted for in the GEE model, many of these lncRNAs were no longer associated with pregnancy, while others became newly associated, similar to the coding genes (**Figure 4B**). Similar results were also obtained at 3 months postpartum.

### Co-expression network analysis

**Figure 5:**
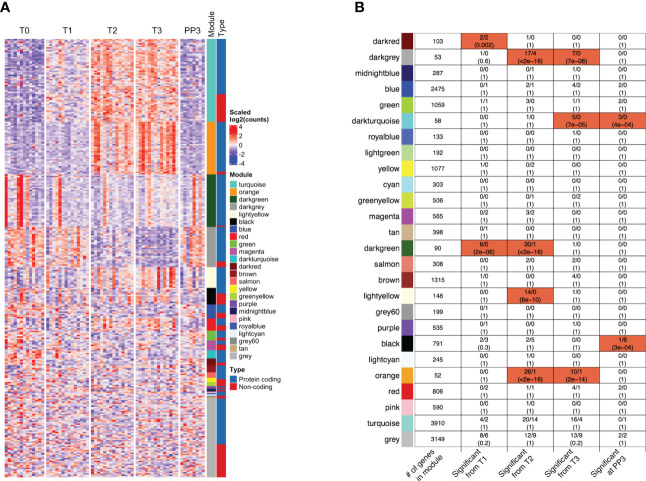
Co-expression of protein-coding genes and/or long non-coding RNAs within functional modules. **(A)** The patterns of expression of the protein-coding genes and lncRNAs whose expression were significantly associated (FC≥2, FDR<0.05) with one or more trimesters of pregnancy in the model adjusted for changes in cell type proportions are shown in the heatmap. Many of the functional modules identified through Weighted Gene Co-expression Network Analysis (*WGCNA*) [sidebar labeled Module] included lncRNAs that were co-expressed with the coding genes [shown in sidebar labeled Type]. **(B)** The total number of genes (among all genes analyzed) that clustered within each WGCNA co-expression module (with color labels) are shown in the left-most column. For each module, the four columns to the right indicate the number of genes (protein-coding/lncRNAs) that become significantly associated with pregnancy at different stages. The modules highlighted in red were significantly enriched in genes/lncRNAs whose expression were associated with pregnancy in specific trimesters or at 3 months postpartum (FDR values shown). Co-expression of protein-coding genes and lncRNAs was also observed in several modules (darkred, darkgrey, blue, green, magenta, darkgreen, black, orange, red, turquoise and grey). [T1, 1^st^ trimester; T2, 2^nd^ trimester; T3, 3^rd^ trimester; PP3: 3 months postpartum].

Weighted Gene Co-expression Network Analysis (WGCNA) of all genes analyzed in the adjusted GEE model identified 25 co-expression modules, i.e. densely connected sub-networks of genes with highly correlated expression. Of the protein-coding genes and lncRNAs with pregnancy-associated expression, those that clustered together within a co-expression module showed similar patterns of expression, as expected (**Figure 5A**). Additionally, within each module, we profiled enrichment of pregnancy-associated genes and lncRNAs (**Figure 5B**). Genes and lncRNAs showing pregnancy-associated expression were enriched in specific modules in a time-dependent manner. For example, the time-points at which they were associated with pregnancy and the modules in which they were enriched were as follows - T1 only: darkred module (enrichment FDR=0.002); T1 and T2: darkgreen module (FDR=2E-06); T2 only: lightyellow module (FDR=6E-10); T2 and T3: darkgrey (FDR<2.2E-16) and orange (FDR<2.2E-16) modules; T3 and PP3: darkturquoise module (FDR=7E-05); and finally, PP3 only: black module (FDR=3E-04). Because genes clustered within a module are strongly co-expressed, modules are often related to biological function. The GO terms that each module was enriched in are shown in **Table S3**. While the darkturquoise and darkgrey modules were not enriched in any specific GO terms, darkturquoise consisted mostly of immunoglobulin V and C genes (45/58, 78%) and, based on cell type signature gene sets downloaded from the molecular signatures database, darkgrey was enriched in markers of mast cells (FDR=1.1E-05).

Since the genes with expression changes significant in the adjusted GEE model were likely being transcriptionally regulated, their co-expression with any lncRNAs that could potentially be regulating their expression was examined. There were 11 modules in which both protein-coding genes and lncRNAs were co-expressed: darkred, darkgrey, blue, green, magenta, darkgreen, black, orange, red, turquoise and grey (**Figure 5B**). The specific genes and lncRNAs co-expressed in those modules, together with GO terms associated with the modules are shown in **Table S3**. Of interest, the Darkgreen module that was functionally related to type I interferon signaling included Type I interferon-inducible genes co-expressed with lncRNA AC131011.1. Within the Orange module, several neutrophil-related genes were co-expressed with lncRNAs LINC00900 and LINC02009. In the Turquoise module, some other neutrophil-related genes were co-expressed with several lncRNAs including known and novel ones identified in our data.

## Discussion

We present here the findings from our unique pregnancy cohort of healthy women with term pregnancies that, unlike previous cohorts, includes a pre-pregnancy baseline. The women were enrolled in our cohort while they were still planning a pregnancy and they were prospectively followed through pregnancy and postpartum. Thus, this is the first report of systemic changes in the expression of protein-coding genes and lncRNAs that occur among healthy women with term pregnancies from pre-pregnancy, through each trimester, until 3 months postpartum, based on RNA-seq data from the maternal periphery. Here, we have focused only on healthy Danish women (n=14), demonstrating progressive changes in expression patterns at each trimester, compared to the pre-pregnancy baseline, and how those changes relate to cell type proportion, transcriptional regulation and biological function. We have also examined co-expression of the protein-coding genes and lncRNAs showing pregnancy-associated expression within functional modules.

Overall, the pregnancy-associated patterns of gene expression from our unadjusted model were very similar to what we had previously described for patterns that overlapped between healthy women and women with RA (7). In our present dataset, the small degree of overlap in the findings from the unadjusted and adjusted GEE models provide evidence that, to a large extent, pregnancy-related changes that occur in the maternal periphery result from changes in the proportions of different cell populations during the course of pregnancy. Nonetheless, there was also evidence that a small proportion of pregnancy-associated changes were the result of transcriptional regulation. Several of the genes that appeared to be transcriptionally regulated were also found to be target genes for transcription factors. These included several type I interferon-inducible genes whose expression were down-regulated during pregnancy. We speculate that this is a mechanism to maintain “normal” levels of these genes and their products, and promote a healthy pregnancy, since high levels of type I interferons and interferon-inducible genes during pregnancy have been associated with preeclampsia and other fetal complications (40–42). Co-expression network analysis showed that many of the transcriptionally regulated genes were highly co-expressed and functionally related within different modules, and these genes within a module were being modulated during pregnancy in a time-dependent manner. Another interesting finding was that, for a large proportion of genes that showed significant changes in expression by 3 months postpartum, those postpartum changes were inversely correlated with their changes during pregnancy. Thus, clearly, these genes reverted their expression back to pre-pregnancy levels after childbirth.

There were many neutrophil-related genes among those that showed the largest increases in expression during pregnancy (2- to 27-fold) compared to the T0 baseline. This neutrophil signature is consistent with the notable increase in our estimated proportions of neutrophils in the maternal periphery during pregnancy. This pregnancy-related increase in neutrophils is supported by previous reports (13, 43). Additionally, our results from the GEE models demonstrate that many of the neutrophil-related genes (such as CD177, OLFM4, CEACAM6/8, DEFA1/3/4, S100A8/9/12, S100P, MMP8, CRISP3, CAMP and ELANE) were significantly associated with pregnancy in the models unadjusted and adjusted for cell type proportions. These findings suggest that, while the increasing prominence of the neutrophil expression signature during pregnancy was in part due to increasing numbers of neutrophils in the bloodstream, these genes were also being more actively transcribed (2- to 15-fold compared to T0) as a result of transcriptional regulation. Yet, little is known about the function of neutrophils in the context of pregnancy. It has been postulated that, in general, neutrophils can assume pro- or anti-inflammatory, as well as immune-regulatory roles (43). Thus, while they have been implicated in pregnancy pathologies (44, 45), our findings in the circulation of healthy mothers with term pregnancies show that they also have important roles in maintaining a healthy pregnancy.

We found numerous lncRNAs to be expressed during and after pregnancy, in a trimester-associated pattern. While we do not know what regulated the expression of these specific lncRNAs in a temporal manner, it is of interest that expression of some lncRNAs can be modulated by estrogen (46). Further, co-expression within a module has been shown to correlate with functional relatedness (47). Thus, the co-expression of lncRNAs within the same functional modules as protein-coding genes whose expression are associated with pregnancy suggests that those genes and lncRNAs likely share similar functions. Additionally, the lncRNAs could potentially be involved in regulating expression of the co-expressed genes, especially since we demonstrated that the expression changes among those genes were, at least in part, due to transcriptional regulation. Although the lncRNAs did not map to the regulatory regions of the genes that they were co-expressed with (data not shown), there still remains a possibility that they may be epigenetically regulating the expression of those genes.

There is some overlap between our findings and what others have previously reported, although differences in study design and the lack of a pre-pregnancy baseline in those studies did not allow direct comparisons. Using RNA from whole blood collected in PAXgene tubes, Gomez-Lopez et al. (12) had observed increased expression (>1.5-fold) of CD177, ARG1, CAMP, MMP8, DEFA4, S100A12 and OLFM4, among others, and decreased expression of some immunoglobulin genes during pregnancy. The magnitudes of the expression changes were modest compared to what we observed, probably because the reported changes were relative to a baseline of late first trimester (gestational week 10) in that study, whereas we report here changes relative to a pre-pregnancy baseline. Additionally, this study also found that expression of genes that were the most highly modulated increased with advancing gestational age, which we also observed in our data. Another observation from that study was that a large number of genes associated with pregnancy mapped to chromosome 14. While this was replicated in our data (not shown), we did not consider that to be a significant finding because that was due to the large number of immunoglobulin genes, which map to chromosome 14, being significantly modulated by pregnancy. Based on cell type proportions estimated from DNA methylation data from peripheral blood mononuclear cells (PBMCs), Knight et al. (11) reported a significant decrease in the proportion of B cells and natural killer (NK) cells during uncomplicated term pregnancies, which is consistent with the decrease in estimated naïve B cells and NK cells in our data. There was some degree of overlap in genes with pregnancy-associated expression between that study and ours (for example, CEACAM6/8, CRISP3, DEFA1/3/4, ELANE, IFIT1B, IFI27, MMP8/9, OLFM4, S100P and S100A12), although the specific changes were not comparable since that study used data from only 2 broad time-points during pregnancy (gestational weeks 6-15 and 22-33). Hong et al. (13) reported an increase in neutrophils during pregnancy and a decrease postpartum, using cell sorting, thus supporting our observations. While gene expression results were not directly comparable between that study and ours, reduced expression of interferon-related genes and increased expression of neutrophil-related genes were observed during pregnancy in both studies. Some of the genes that had pregnancy-associated expression in our data (OLFM4, CEACAM6/7, DEFA3/4, MMP8/9, CD177, IFI44, IFI27, ELANE, among others) were reported by Heng et al. (9) to be the most differentially expressed between T2 and T3 in healthy term pregnancies, although fold-changes were very modest (1.2-1.9), as can be expected between T2 and T3. We found little overlap between the most up- or down-regulated genes in our data and those reported by Al-Garawi et al. (T3 vs T1) (10).

Our study has many strengths: (1) The availability of time-dependent data from the same women at different time-points before, during and after pregnancy allowed pregnancy-induced gene expression changes to be compared to a pre-pregnancy baseline, while at the same time controlling for unmeasured confounders; (2) The homogeneous genetic background of the study population was an advantage for the gene expression studies performed; (3) The use of RNA-seq technology to assess gene expression was a significant advantage over microarray data used in previous studies; (4) Using RNA from whole blood (rather than PBMCs only) enabled us to examine overall systemic gene expression changes that occur in the maternal periphery during and after pregnancy, and to identify the neutrophil signature during pregnancy. Further, since the RNA samples were stabilized in PAXgene tubes right at the time of each blood draw, the expression profiles are likely to reflect those *in vivo*; (5) Adjusting for cell type proportions in the GEE model and comparing to the unadjusted model enabled us to tease out which pregnancy-induced expression changes were due to changes in cell type proportions and which were brought on by epigenetic regulation of transcription. (6) The use of total RNA enabled us to examine both protein-coding and lncRNAs from the same sample. Furthermore, in addition to providing insight into gene expression changes that happen during pregnancy and postpartum among healthy women, these findings also bear significance for studies of abnormal pregnancies or autoimmune diseases that improve or worsen during pregnancy, such as rheumatoid arthritis. That is, using the expression changes observed in normal healthy pregnancies as a baseline makes it possible to assess which genes in disease conditions show expression changes that deviate from normal. Our findings presented here from healthy women with term pregnancies provide the first such normal reference. There are also limitations in our study. First, the sample size was relatively small. However, the availability of longitudinal data from the same women enabled us to overcome some of the limitations of having a small sample size. Furthermore, to our knowledge, this is the only pregnancy cohort of healthy women currently available with a pre-pregnancy baseline. Thus, even though the sample was small, our findings add new knowledge to the current literature, advancing this field forward. Second, while the ethnic homogeneity of the study subjects was a strength for gene expression studies, that limits the extent to which the findings can be generalized to non-Caucasian populations. Third, the use of total RNA from whole blood could imply that expression profiles of neutrophils dominated a large part of the observed expression patterns. However, the sensitivity of RNA-seq technology enabled us to also detect transcripts that were not neutrophil-specific, including those specific to cell types present in low proportions in blood. Fourth, RNA from whole blood originates from a heterogeneous mixture of cells, and therefore, it was not possible to identify cell type-specific expression changes associated with pregnancy. Last, we did not collect data on whether the pregnancies were uncomplicated or not.

## Conclusion

In summary, our results demonstrate that, compared to a pre-pregnancy baseline, there are extensive systemic changes in cell type proportions, expression of genes and lncRNAs, and biological pathways that are associated with each trimester of term pregnancies and postpartum among healthy women. The expression changes in the maternal bloodstream are in large part due to changes in cell type proportions, although epigenetic control of transcriptional regulation also appears to play an important role. Whether lncRNAs are partly responsible for this transcriptional regulation is not clear. Nonetheless, those lncRNAs co-expressed with genes showing pregnancy-associated expression are excellent candidates as epigenetic regulators of their expression. Overall, our findings represent a “normal reference” to which data from abnormal or disease pregnancies can be compared to assess deviations from normal.

## Data availability statement

The datasets presented in this article are not readily available because the data and materials are protected by the General Data Protection Regulation (GDPR) of the European Union (2016/679), and by the Danish Data Protection Act enacted in May 2018 to supplement the GDPR. Requests to access the analyses of the dataset should be directed to Damini.Jawaheer@northwestern.edu.

## Ethics statement

The studies involving human participants were reviewed and approved by Ethics Committee for Region Hovedstaden (Denmark), the Children’s Hospital Oakland Research Institute Institutional Review Board (IRB), and the Northwestern University IRB. The patients/participants provided their written informed consent to participate in this study.

## Author contributions

MW analyzed the data, interpreted the results and contributed in writing the original draft of the manuscript. JLN and JO contributed to the conceptualization and design of the study. MS was responsible for acquisition of the data. MH and VZ contributed to the data acquisition. DG and NJ contributed to the methodology for the longitudinal data analyses and interpretation of the results. DJ contributed to the conceptualization and design of the overall study and the experiments, to the analysis and interpretation of the data, and to writing the original draft of the manuscript. All authors contributed to the article and approved the submitted version.

## Supplementary material

Supplementary Figure 1 PCA plot of normalized counts for all samples. Principal Components Analysis (PCA) plot of z-score estimates for the 500 genes with the highest variance, for all samples included in the analysis at all time-points (T0 to PP9), following rigorous quality control of the data. PC1 (x-axis) separates the non-pregnancy samples on the left (i.e. T0, PP3, PP6 and PP9) from the mid- to late-pregnancy samples on the right (T2 and T3), with early pregnancy (T1) samples being in the middle. Extensive overlap was observed between T2 and T3 clusters, as may be expected, while the postpartum sample clusters overlapped partly with the T1 cluster and partly with the T0 cluster. [T0: Pre-pregnancy; T1: 1^st^ trimester; T2: 2^nd^ trimester; T3: 3^rd^ trimester; PP3: 3 months postpartum; PP6: 6 months postpartum; PP9: 9 months postpartum].

Supplementary Figure 2 Changes in relative proportions of different cell populations from pre-pregnancy to 3 months postpartum. The box plots show how the relative proportions of different cell types estimated using CIBERSORTx changed from pre-pregnancy (T0) to 3 months postpartum (PP3) among healthy women in our dataset. Only cell types included in the LM22 reference dataset are shown. For some LM22 cell types, proportion estimates were not obtained from CIBERSORTx; those are not shown here (resting dendritic cells, eosinophils, macrophages M1, activated mast cells, activated NK cells, follicular helper T cells, gamma delta T cells). [T0: Pre-pregnancy; T1: 1^st^ trimester; T2: 2^nd^ trimester; T3: 3^rd^ trimester; PP3: 3 months postpartum].
