# Supplementary figures and images for: Pregnancy-associated systemic gene expression compared to a pre-pregnancy baseline, among healthy women with term pregnancies
Source: Front Immunol. 2023 Jun 5;14:1161084. doi: 10.3389/fimmu.2023.1161084 (PMC10277629; doi:10.3389/fimmu.2023.1161084)

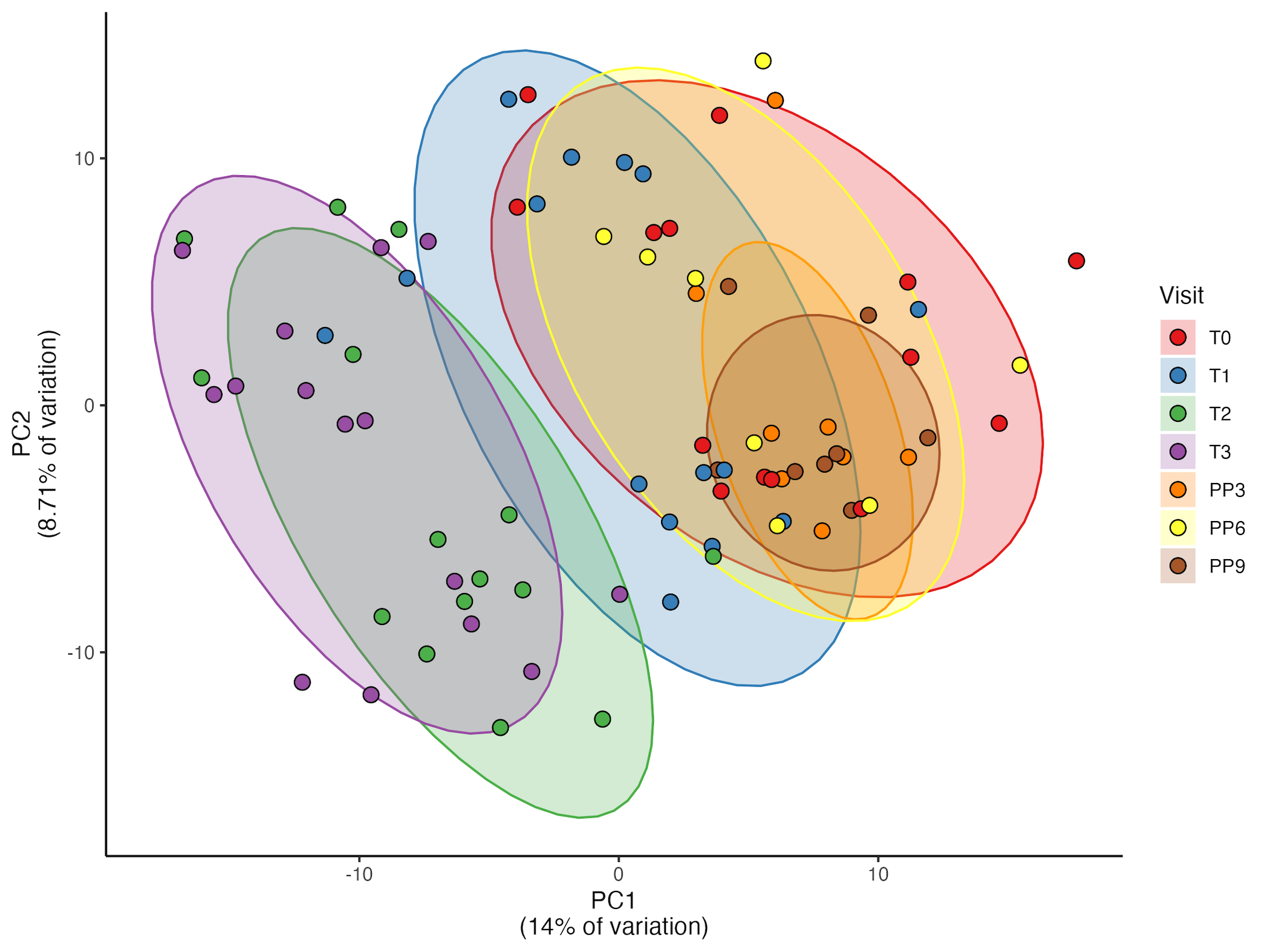

Supplement: Supplementary Figure 1 — PCA plot of normalized counts for all samples. Principal Components Analysis (PCA) plot of z-score estimates for the 500 genes with the highest variance, for all samples included in the analysis at all time-points (T0 to PP9), following rigorous quality control of the data. PC1 (x-axis) separates the non-pregnancy samples on the left (i.e. T0, PP3, PP6 and PP9) from the mid- to late-pregnancy samples on the right (T2 and T3), with early pregnancy (T1) samples being in the middle. Extensive overlap was observed between T2 and T3 clusters, as may be expected, while the postpartum sample clusters overlapped partly with the T1 cluster and partly with the T0 cluster. [T0: Pre-pregnancy; T1: 1st trimester; T2: 2nd trimester; T3: 3rd trimester; PP3: 3 months postpartum; PP6: 6 months postpartum; PP9: 9 months postpartum]. [file Image_1.tiff]

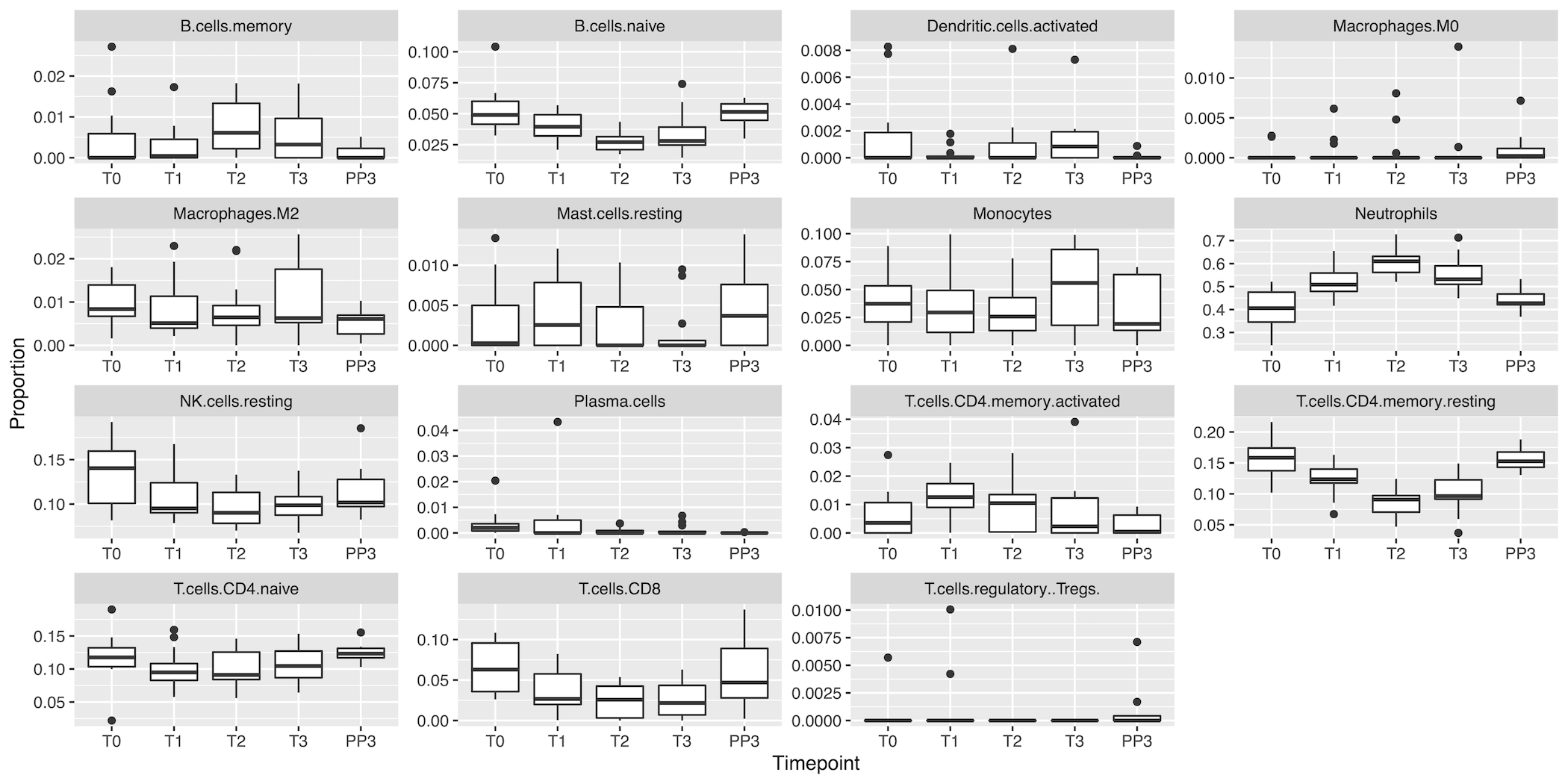

Supplement: Supplementary Figure 2 — Changes in relative proportions of different cell populations from pre-pregnancy to 3 months postpartum. The box plots show how the relative proportions of different cell types estimated using CIBERSORTx changed from pre-pregnancy (T0) to 3 months postpartum (PP3) among healthy women in our dataset. Only cell types included in the LM22 reference dataset are shown. For some LM22 cell types, proportion estimates were not obtained from CIBERSORTx; those are not shown here (resting dendritic cells, eosinophils, macrophages M1, activated mast cells, activated NK cells, follicular helper T cells, gamma delta T cells). [T0: Pre-pregnancy; T1: 1st trimester; T2: 2nd trimester; T3: 3rd trimester; PP3: 3 months postpartum]. [file Image_2.tiff]
