# Supplementary material for: Pregnancy-associated systemic gene expression compared to a pre-pregnancy baseline, among healthy women with term pregnancies
Source: Front Immunol. 2023 Jun 5;14:1161084. doi: 10.3389/fimmu.2023.1161084 (PMC10277629; doi:10.3389/fimmu.2023.1161084)
Supplement: Supplementary file 3 [file Table_1.docx]

**Table S1.**

**Genes with significant pregnancy-associated expression in the GEE model unadjusted for cell type proportions.**

Fold-changes (FCs) in expression at each trimester (compared to the pre-pregnancy [T0] baseline) are shown (to 1 decimal place), together with FDR-adjusted p values (FDR), for genes that were significantly associated with pregnancy (FC≤-2 or FC≥2, FDR<0.05). FCs corresponding to a decrease in expression are shown as negative values. FC and FDR values in bold indicate at which trimester a gene first became significantly associated with pregnancy.

|  | **T1** | | **T2** | | **T3** | |
| --- | --- | --- | --- | --- | --- | --- |
| **Gene name** | **FC (vs T0)** | **FDR** | **FC (vs T0)** | **FDR** | **FC (vs T0)** | **FDR** |
| IGLV1-36 | **-4.1** | **2.2E-03** | -5.3 | 2.9E-04 | -5.2 | 4.7E-03 |
| CCL2 | **-3.7** | **4.0E-02** | -5.9 | 1.2E-02 | -5.8 | 6.3E-02 |
| IDO1 | **-3.1** | **9.4E-08** | -2.5 | 3.4E-04 | -2.1 | 2.9E-03 |
| IGLV3-21 | **-2.5** | **2.4E-02** | -3.8 | 1.9E-06 | -3.4 | 2.4E-04 |
| IL34 | **-2.5** | **5.0E-06** | -2.1 | 2.4E-02 | -2.2 | 1.3E-02 |
| OTOF | **-2.5** | **4.1E-03** | -3.8 | 1.3E-04 | -2.1 | 8.9E-02 |
| PRSS41 | **-2.5** | **1.2E-03** | -3.6 | 2.4E-03 | -1.7 | 3.9E-02 |
| PLAAT5 | **-2.3** | **3.3E-07** | -1.9 | 5.2E-02 | -2.0 | 1.4E-02 |
| IGHV1-46 | **-2.2** | **3.5E-02** | -3.3 | 4.9E-03 | -3.7 | 7.8E-03 |
| IGLV1-47 | **-2.2** | **4.1E-04** | -3.5 | 2.7E-10 | -3.2 | 3.8E-07 |
| IGLV4-69 | **-2.2** | **1.2E-02** | -3.3 | 1.5E-06 | -3.1 | 2.5E-04 |
| IGHV3-13 | **-2.1** | **2.9E-05** | -1.7 | 7.8E-01 | -2.1 | 8.1E-03 |
| RPL3L | **-2.1** | **2.7E-02** | -3.5 | 1.3E-06 | -3.1 | 4.2E-04 |
| ADAMTS14 | **-2.0** | **1.1E-03** | -1.9 | 7.0E-06 | -1.2 | 5.6E-01 |
| CHD5 | **-2.0** | **8.8E-03** | -1.4 | 5.6E-02 | -2.0 | 4.8E-03 |
| OR13A1 | **-2.0** | **1.5E-02** | -1.4 | 5.5E-02 | -1.3 | 2.7E-01 |
| OR3A1 | **-2.0** | **5.3E-05** | -1.1 | 5.3E-01 | -1.2 | 2.6E-01 |
| SIGLEC8 | **-2.0** | **2.1E-08** | -1.9 | 1.4E-02 | -1.8 | 6.9E-02 |
| SPATA9 | **-2.0** | **5.0E-05** | -1.4 | 6.1E-02 | -1.5 | 6.1E-02 |
| AQP9 | **2.0** | **6.3E-14** | 2.8 | 9.4E-36 | 2.3 | 2.4E-16 |
| BCL6 | **2.0** | **2.0E-15** | 2.7 | 1.5E-34 | 2.2 | 1.1E-13 |
| CMTM2 | **2.0** | **1.4E-11** | 2.8 | 9.7E-33 | 2.5 | 1.1E-19 |
| CR1 | **2.0** | **8.6E-09** | 3.0 | 1.6E-30 | 2.6 | 1.2E-15 |
| DEFA1 | **2.0** | **5.8E-03** | 17.3 | 8.4E-17 | 18.9 | 4.5E-19 |
| DSC2 | **2.0** | **1.4E-23** | 3.9 | 0.0E+00 | 3.6 | 2.3E-30 |
| FFAR3 | **2.0** | **3.4E-02** | 4.1 | 1.1E-10 | 3.2 | 1.3E-07 |
| G0S2 | **2.0** | **1.2E-04** | 3.0 | 6.0E-12 | 2.5 | 1.2E-08 |
| GCA | **2.0** | **1.9E-25** | 3.1 | 0.0E+00 | 2.6 | 1.6E-21 |
| GLIS2 | **2.0** | **6.5E-03** | 3.2 | 9.0E-12 | 2.9 | 3.0E-05 |
| HTRA3 | **2.0** | **3.8E-02** | 9.4 | 1.9E-29 | 13.1 | 6.8E-17 |
| MME | **2.0** | **1.2E-10** | 2.4 | 1.0E-17 | 1.9 | 1.1E-05 |
| MRVI1 | **2.0** | **1.7E-10** | 2.9 | 3.1E-24 | 2.4 | 5.2E-15 |
| NAIP | **2.0** | **1.0E-09** | 3.3 | 1.0E-18 | 2.8 | 3.5E-14 |
| NAMPT | **2.0** | **1.7E-30** | 2.8 | 0.0E+00 | 2.3 | 2.5E-21 |
| NECAB2 | **2.0** | **3.3E-05** | 2.7 | 2.0E-10 | 2.1 | 4.1E-04 |
| NKAPL | **2.0** | **7.1E-05** | 2.0 | 3.8E-03 | 1.5 | 2.6E-02 |
| PAPPA2 | **2.0** | **5.6E-03** | 3.0 | 2.5E-07 | 3.2 | 3.0E-15 |
| PPP1R3B | **2.0** | **3.5E-20** | 2.7 | 2.5E-34 | 2.2 | 9.6E-16 |
| PYGL | **2.0** | **9.9E-13** | 2.8 | 2.7E-32 | 2.4 | 4.2E-16 |
| SCRT2 | **2.0** | **4.6E-02** | 1.5 | 1.2E-01 | 2.3 | 4.2E-02 |
| SEMA6B | **2.0** | **1.0E-08** | 3.9 | 3.9E-22 | 3.3 | 6.2E-14 |
| SEMG1 | **2.0** | **3.9E-02** | 2.8 | 8.9E-05 | 2.9 | 3.1E-03 |
| SLC26A8 | **2.0** | **2.7E-07** | 3.1 | 8.5E-24 | 2.7 | 1.8E-14 |
| SLC2A3 | **2.0** | **3.5E-16** | 2.7 | 0.0E+00 | 2.2 | 2.7E-18 |
| TMEM92 | **2.0** | **1.1E-02** | 3.5 | 2.6E-09 | 3.7 | 2.7E-07 |
| VSIG8 | **2.0** | **1.1E-02** | 1.4 | 4.9E-02 | 1.6 | 1.7E-02 |
| ACSL1 | **2.1** | **4.2E-12** | 3.1 | 3.5E-34 | 2.5 | 4.9E-16 |
| ADGRG3 | **2.1** | **8.1E-11** | 2.9 | 2.1E-34 | 2.5 | 4.8E-19 |
| BASP1 | **2.1** | **3.6E-14** | 2.9 | 0.0E+00 | 2.4 | 4.2E-16 |
| BCL2A1 | **2.1** | **2.0E-10** | 3.7 | 5.7E-36 | 3.5 | 1.5E-20 |
| FAM169B | **2.1** | **7.4E-08** | 3.0 | 1.2E-17 | 2.4 | 1.9E-07 |
| HPD | **2.1** | **1.7E-02** | 3.1 | 8.8E-05 | 2.4 | 1.7E-07 |
| KAZN | **2.1** | **2.0E-09** | 3.7 | 8.9E-20 | 3.1 | 3.6E-15 |
| LRG1 | **2.1** | **1.4E-14** | 3.2 | 0.0E+00 | 2.6 | 1.2E-21 |
| MANSC1 | **2.1** | **2.9E-16** | 2.5 | 5.1E-23 | 2.1 | 2.5E-10 |
| MGAM | **2.1** | **1.5E-10** | 2.8 | 1.7E-27 | 2.5 | 2.6E-13 |
| PFKFB3 | **2.1** | **2.1E-08** | 3.2 | 1.5E-35 | 2.8 | 8.9E-20 |
| S100A9 | **2.1** | **9.1E-10** | 3.4 | 0.0E+00 | 3.4 | 0.0E+00 |
| SFN | **2.1** | **3.1E-04** | 2.4 | 7.5E-07 | 2.2 | 3.8E-05 |
| SHROOM4 | **2.1** | **1.1E-06** | 4.1 | 4.8E-32 | 3.6 | 9.3E-17 |
| SLC51A | **2.1** | **6.3E-04** | 6.7 | 1.5E-31 | 7.5 | 1.2E-34 |
| SYN2 | **2.1** | **4.0E-03** | 4.5 | 1.6E-22 | 3.4 | 3.3E-15 |
| TXNDC2 | **2.1** | **2.5E-03** | 2.4 | 1.1E-06 | 1.9 | 1.2E-03 |
| ZDHHC19 | **2.1** | **6.0E-05** | 5.7 | 5.0E-23 | 6.4 | 2.2E-19 |
| ZNF112 | **2.1** | **1.2E-03** | 1.6 | 4.5E-02 | 1.6 | 4.6E-02 |
| CRISP3 | **2.2** | **1.5E-03** | 9.9 | 9.0E-27 | 13.8 | 6.2E-36 |
| IL10 | **2.2** | **7.9E-03** | 1.7 | 1.5E-02 | 2.0 | 2.6E-02 |
| MGAM2 | **2.2** | **5.3E-08** | 3.3 | 1.4E-16 | 2.8 | 4.5E-11 |
| SLPI | **2.2** | **8.2E-09** | 4.0 | 0.0E+00 | 3.9 | 1.8E-33 |
| TLR5 | **2.2** | **1.7E-13** | 3.6 | 0.0E+00 | 3.2 | 6.4E-25 |
| ADM | **2.3** | **2.5E-13** | 3.2 | 2.1E-31 | 2.7 | 5.1E-16 |
| DOK4 | **2.3** | **4.1E-09** | 3.6 | 2.6E-24 | 2.9 | 8.9E-10 |
| LYVE1 | **2.3** | **3.6E-07** | 3.3 | 2.2E-13 | 2.6 | 9.3E-11 |
| MMP9 | **2.3** | **2.5E-07** | 3.7 | 7.0E-33 | 3.5 | 6.9E-25 |
| OPLAH | **2.3** | **1.3E-07** | 3.4 | 0.0E+00 | 3.2 | 5.8E-20 |
| OR56A1 | **2.3** | **4.5E-02** | 3.1 | 2.2E-03 | 2.7 | 1.2E-02 |
| ORM1 | **2.3** | **2.6E-04** | 6.8 | 1.6E-14 | 6.3 | 1.7E-16 |
| OSM | **2.3** | **4.2E-09** | 4.1 | 0.0E+00 | 3.3 | 2.7E-32 |
| S100P | **2.3** | **3.3E-07** | 3.7 | 6.9E-24 | 3.4 | 7.8E-16 |
| TRIM9 | **2.3** | **1.2E-15** | 3.4 | 2.7E-20 | 2.9 | 6.7E-10 |
| C5orf67 | **2.4** | **2.8E-05** | 3.2 | 4.2E-13 | 3.0 | 1.9E-07 |
| CA4 | **2.4** | **6.6E-13** | 3.9 | 1.8E-28 | 3.4 | 1.2E-15 |
| PGLYRP1 | **2.4** | **6.2E-14** | 4.3 | 0.0E+00 | 4.2 | 0.0E+00 |
| PROK2 | **2.4** | **1.4E-13** | 3.6 | 7.5E-30 | 2.9 | 6.4E-15 |
| ARG1 | **2.5** | **1.0E-11** | 6.1 | 0.0E+00 | 6.6 | 0.0E+00 |
| CAMP | **2.5** | **1.3E-13** | 9.4 | 1.6E-33 | 11.3 | 0.0E+00 |
| INHBB | **2.5** | **9.7E-06** | 4.2 | 1.8E-26 | 3.5 | 3.5E-17 |
| S100A8 | **2.5** | **2.0E-10** | 5.1 | 2.8E-32 | 5.7 | 4.6E-20 |
| SOCS3 | **2.5** | **3.8E-11** | 4.5 | 0.0E+00 | 3.4 | 2.3E-28 |
| BMX | **2.6** | **2.4E-12** | 4.1 | 1.3E-26 | 3.6 | 8.5E-20 |
| GNG10 | **2.7** | **4.9E-06** | 3.0 | 1.7E-08 | 3.3 | 1.1E-07 |
| KREMEN1 | **2.7** | **1.8E-14** | 4.4 | 0.0E+00 | 3.7 | 4.4E-27 |
| S100A12 | **2.7** | **1.2E-09** | 5.7 | 0.0E+00 | 6.1 | 0.0E+00 |
| ALPL | **2.8** | **1.8E-14** | 4.2 | 2.0E-36 | 3.7 | 2.0E-17 |
| ANOS1 | **2.8** | **1.6E-09** | 6.7 | 0.0E+00 | 7.0 | 0.0E+00 |
| MCEMP1 | **2.8** | **8.3E-08** | 7.1 | 0.0E+00 | 6.5 | 0.0E+00 |
| MMP8 | **2.8** | **6.1E-07** | 17.3 | 0.0E+00 | 24.1 | 0.0E+00 |
| ANXA3 | **2.9** | **5.1E-15** | 6.0 | 0.0E+00 | 5.2 | 1.8E-33 |
| AP3B2 | **3.1** | **4.6E-10** | 5.5 | 1.7E-30 | 5.0 | 3.2E-30 |
| ITGB4 | **3.2** | **1.3E-08** | 6.0 | 2.7E-25 | 5.4 | 8.6E-11 |
| GALNT14 | **3.4** | **5.6E-14** | 6.1 | 4.5E-34 | 4.6 | 3.1E-20 |
| ADAMTS2 | **3.5** | **2.4E-04** | 5.0 | 7.4E-04 | 4.4 | 4.1E-03 |
| COX6B2 | **3.7** | **6.7E-06** | 8.0 | 7.9E-15 | 7.0 | 1.2E-13 |
| NDST3 | **3.7** | **4.4E-03** | 7.7 | 1.8E-08 | 6.9 | 6.9E-06 |
| INSC | **3.9** | **5.4E-05** | 6.8 | 4.1E-09 | 5.9 | 2.4E-07 |
| CAPN13 | **4.1** | **4.0E-10** | 8.8 | 9.6E-26 | 7.4 | 8.3E-21 |
| CBSL | **4.5** | **2.5E-04** | 7.7 | 5.6E-10 | 6.0 | 1.2E-05 |
| CD177 | **8.7** | **4.5E-10** | 27.2 | 0.0E+00 | 25.6 | 0.0E+00 |
| C10orf82 | 1.4 | 8.9E-01 | **-6.9** | **1.7E-04** | -32.8 | 2.1E-07 |
| IGLV3-27 | -1.7 | 2.3E-01 | **-5.7** | **3.1E-03** | -4.6 | 6.2E-01 |
| RSAD2 | -3.6 | 2.3E-01 | **-4.4** | **2.7E-02** | -3.1 | 1.0E-01 |
| KRT86 | -1.4 | 1.3E-01 | **-4.3** | **3.3E-04** | -3.5 | 3.4E-04 |
| IGHA1 | -1.9 | 3.3E-01 | **-4.0** | **1.2E-08** | -4.3 | 7.7E-09 |
| ITGB8 | -1.6 | 8.7E-02 | **-4.0** | **5.8E-08** | -10.4 | 1.6E-33 |
| LAMP3 | -2.8 | 1.3E-01 | **-4.0** | **1.2E-02** | -3.2 | 3.6E-02 |
| IGHV6-1 | -1.8 | 5.9E-01 | **-3.8** | **1.6E-06** | -2.9 | 5.7E-05 |
| GPRC5D | -1.3 | 7.3E-01 | **-3.6** | **2.8E-03** | -4.3 | 3.9E-03 |
| IGHV2-70 | -2.3 | 1.9E-01 | **-3.6** | **4.3E-03** | -3.1 | 2.1E-03 |
| USP18 | -2.9 | 9.6E-02 | **-3.5** | **1.0E-02** | -2.8 | 4.1E-02 |
| GATA2 | -1.5 | 3.4E-03 | **-3.4** | **9.2E-10** | -7.0 | 0.0E+00 |
| GLDC | -1.5 | 5.7E-01 | **-3.4** | **4.8E-10** | -3.5 | 3.0E-05 |
| HDC | -1.5 | 3.2E-01 | **-3.4** | **3.1E-06** | -9.1 | 0.0E+00 |
| **IFI44L** | -3.0 | 3.0E-01 | **-3.4** | **2.7E-02** | -2.3 | 2.6E-01 |
| IGKV1D-13 | -1.7 | 1.9E-01 | **-3.4** | **5.3E-04** | -3.6 | 9.4E-03 |
| SDC1 | -1.1 | 8.2E-01 | **-3.4** | **4.6E-08** | -2.0 | 9.1E-03 |
| IGHV2-70D | -1.9 | 1.8E-03 | **-3.3** | **1.2E-02** | -2.9 | 1.7E-02 |
| IL4 | -1.6 | 2.4E-01 | **-3.3** | **6.7E-06** | -11.2 | 1.0E-15 |
| **OAS3** | -2.6 | 1.8E-01 | **-3.3** | **8.3E-03** | -2.5 | 6.0E-02 |
| IGHG1 | -1.8 | 6.9E-02 | **-3.2** | **1.6E-05** | -2.7 | 9.7E-03 |
| IGHV4-61 | -1.2 | 7.0E-01 | **-3.2** | **1.1E-05** | -2.4 | 1.3E-03 |
| IGKV2-24 | -1.1 | 7.4E-01 | **-3.2** | **4.4E-08** | -2.8 | 1.7E-09 |
| ISG15 | -2.7 | 2.2E-01 | **-3.2** | **4.7E-02** | -2.5 | 1.1E-01 |
| IGHV3-64 | -1.4 | 6.8E-02 | **-3.1** | **8.1E-15** | -3.4 | 1.4E-05 |
| IGKV1-17 | -1.8 | 1.2E-01 | **-3.1** | **4.5E-02** | -2.8 | 7.3E-03 |
| IGKV1-6 | -1.3 | 4.2E-01 | **-3.1** | **1.4E-03** | -2.6 | 3.3E-02 |
| IGLV7-43 | -1.4 | 5.4E-01 | **-3.1** | **1.6E-03** | -2.6 | 4.6E-06 |
| MS4A2 | -1.4 | 1.6E-01 | **-3.1** | **2.3E-04** | -7.3 | 0.0E+00 |
| AGRN | -2.2 | 1.4E-01 | **-3.0** | **1.2E-02** | -2.2 | 5.2E-02 |
| IGHA2 | -1.3 | 4.8E-01 | **-3.0** | **1.1E-03** | -3.3 | 2.2E-07 |
| IGHV1-69D | -1.3 | 7.9E-01 | **-3.0** | **2.5E-03** | -2.2 | 8.8E-02 |
| IGHV3-7 | -1.6 | 9.0E-01 | **-3.0** | **1.4E-03** | -3.4 | 2.5E-04 |
| IGHV5-51 | -1.8 | 2.4E-02 | **-3.0** | **1.4E-09** | -2.8 | 8.3E-06 |
| IGKV1-9 | -2.0 | 1.3E-01 | **-3.0** | **2.0E-02** | -2.6 | 3.0E-02 |
| AKAP12 | -1.1 | 5.2E-01 | **-2.9** | **1.8E-05** | -10.3 | 0.0E+00 |
| ENPP3 | -1.4 | 2.3E-02 | **-2.9** | **5.5E-10** | -5.4 | 3.2E-22 |
| IGHV4-4 | -1.8 | 5.1E-01 | **-2.9** | **9.3E-04** | -2.7 | 1.1E-03 |
| IGKV4-1 | -1.2 | 9.9E-01 | **-2.9** | **4.9E-05** | -2.6 | 1.5E-04 |
| IGLV1-40 | -1.4 | 4.5E-01 | **-2.9** | **1.5E-06** | -2.4 | 7.8E-03 |
| IGLV1-44 | -1.9 | 3.8E-07 | **-2.9** | **3.8E-04** | -3.1 | 1.2E-03 |
| IGLV2-18 | 1.0 | 6.1E-01 | **-2.9** | **2.9E-02** | -2.0 | 6.6E-02 |
| CCNA1 | -1.7 | 1.2E-01 | **-2.8** | **5.1E-05** | -1.6 | 1.7E-01 |
| FCER1A | -1.4 | 2.4E-01 | **-2.8** | **5.4E-08** | -4.5 | 4.8E-19 |
| IGHV3-30 | -1.3 | 8.7E-01 | **-2.8** | **4.3E-07** | -2.4 | 1.1E-07 |
| IGLC2 | -1.4 | 5.6E-01 | **-2.8** | **2.5E-09** | -2.7 | 8.8E-05 |
| IGLV3-10 | -1.2 | 9.8E-01 | **-2.8** | **1.2E-05** | -2.5 | 1.8E-04 |
| IGHV3-15 | -1.5 | 5.7E-04 | **-2.7** | **4.5E-03** | -3.1 | 7.1E-05 |
| IGKV1-5 | -1.1 | 9.3E-01 | **-2.7** | **4.0E-05** | -2.3 | 8.0E-05 |
| IGKV3-11 | -1.2 | 6.0E-01 | **-2.7** | **8.6E-05** | -2.7 | 3.8E-08 |
| IGLC3 | -1.6 | 5.8E-01 | **-2.7** | **2.5E-07** | -2.5 | 6.2E-06 |
| IGLV2-23 | -1.4 | 2.7E-01 | **-2.7** | **3.3E-08** | -2.4 | 4.3E-03 |
| IGLV3-1 | -1.6 | 1.8E-01 | **-2.7** | **5.1E-03** | -2.4 | 2.7E-01 |
| RMI2 | -2.5 | 8.6E-02 | **-2.7** | **2.1E-02** | -2.6 | 3.4E-02 |
| CACNG6 | -1.9 | 1.4E-05 | **-2.6** | **2.0E-04** | -3.5 | 5.6E-05 |
| CCDC187 | -1.1 | 9.2E-01 | **-2.6** | **7.3E-03** | -2.4 | 2.4E-02 |
| DGKK | -1.4 | 5.3E-02 | **-2.6** | **3.3E-06** | -2.7 | 6.4E-07 |
| ESM1 | -1.5 | 4.9E-02 | **-2.6** | **2.9E-04** | -1.5 | 1.1E-01 |
| IGHV1-69 | -1.4 | 5.0E-01 | **-2.6** | **3.0E-04** | -2.5 | 3.0E-02 |
| IGHV2-26 | -1.7 | 4.7E-02 | **-2.6** | **6.3E-04** | -2.2 | 1.2E-02 |
| IGHV2-5 | -1.4 | 4.6E-02 | **-2.6** | **2.9E-06** | -2.6 | 2.1E-04 |
| IGHV3-43 | -1.8 | 2.8E-02 | **-2.6** | **2.5E-04** | -2.0 | 2.8E-02 |
| IGHV3-48 | -1.2 | 5.5E-01 | **-2.6** | **1.2E-17** | -2.6 | 8.5E-12 |
| IGKC | -1.3 | 9.4E-01 | **-2.6** | **9.8E-06** | -2.7 | 5.8E-07 |
| IGLV3-25 | -1.4 | 2.3E-01 | **-2.6** | **5.2E-04** | -2.5 | 5.5E-08 |
| IGLV6-57 | 1.0 | 3.2E-01 | **-2.6** | **6.4E-06** | -2.6 | 2.2E-04 |
| NLRP7 | -1.1 | 7.1E-01 | **-2.6** | **1.9E-04** | -2.4 | 2.5E-03 |
| SCARA5 | 1.2 | 2.9E-01 | **-2.6** | **1.3E-03** | -1.4 | 2.8E-02 |
| **SERPING1** | -2.0 | 2.9E-01 | **-2.6** | **2.2E-02** | -2.1 | 9.0E-02 |
| DCT | -1.1 | 7.0E-01 | **-2.5** | **7.6E-04** | -1.7 | 2.0E-02 |
| ETV7 | -2.1 | 2.1E-01 | **-2.5** | **4.2E-02** | -2.0 | 1.4E-01 |
| IGHV3-20 | -1.2 | 9.7E-01 | **-2.5** | **2.6E-02** | -1.7 | 1.2E-01 |
| IGKV1-27 | -1.6 | 2.6E-01 | **-2.5** | **5.3E-03** | -2.1 | 4.8E-04 |
| IGKV3-15 | -1.5 | 3.5E-01 | **-2.5** | **3.5E-04** | -2.5 | 7.2E-04 |
| JCHAIN | -1.2 | 7.6E-01 | **-2.5** | **2.3E-10** | -2.5 | 5.2E-07 |
| KCNN3 | -1.7 | 4.7E-01 | **-2.5** | **7.7E-19** | -2.6 | 3.5E-05 |
| MEIS2 | -1.1 | 7.0E-01 | **-2.5** | **6.4E-03** | -1.7 | 2.4E-01 |
| MZB1 | -1.2 | 9.2E-01 | **-2.5** | **1.5E-07** | -2.4 | 2.7E-06 |
| NTRK1 | -1.7 | 2.0E-02 | **-2.5** | **4.1E-03** | -4.1 | 5.3E-13 |
| SLC1A7 | -1.6 | 7.6E-03 | **-2.5** | **8.7E-06** | -2.2 | 7.0E-10 |
| SLC45A3 | -1.4 | 2.0E-03 | **-2.5** | **3.6E-10** | -3.6 | 6.6E-27 |
| SYCP2L | -1.6 | 1.1E-03 | **-2.5** | **2.8E-05** | -2.3 | 9.5E-09 |
| CACNG8 | -1.9 | 3.6E-05 | **-2.4** | **1.4E-03** | -2.6 | 2.9E-03 |
| **IFI44** | -2.3 | 3.7E-01 | **-2.4** | **4.9E-02** | -1.9 | 2.0E-01 |
| IGHV1-18 | -1.5 | 1.0E-01 | **-2.4** | **1.3E-05** | -2.2 | 1.5E-03 |
| IGHV3-33 | -1.3 | 9.5E-01 | **-2.4** | **3.2E-07** | -2.2 | 1.4E-05 |
| IGHV3-49 | -1.1 | 6.0E-01 | **-2.4** | **8.3E-03** | -2.8 | 7.5E-04 |
| IGHV3-73 | -1.4 | 8.0E-01 | **-2.4** | **2.0E-02** | -2.4 | 9.4E-03 |
| IGKV1-8 | -1.6 | 2.2E-07 | **-2.4** | **7.9E-04** | -2.2 | 9.6E-04 |
| IGKV3-20 | -1.3 | 9.9E-01 | **-2.4** | **1.3E-10** | -2.5 | 7.5E-15 |
| IGLV3-19 | -1.8 | 1.2E-01 | **-2.4** | **1.3E-03** | -2.1 | 2.8E-02 |
| IGLV4-60 | -1.2 | 4.8E-01 | **-2.4** | **3.2E-03** | -1.5 | 2.9E-01 |
| SH3RF2 | -1.7 | 6.3E-03 | **-2.4** | **1.5E-05** | -1.6 | 1.5E-03 |
| TMEM132C | 1.1 | 9.7E-01 | **-2.4** | **5.7E-03** | -1.2 | 3.5E-01 |
| AC233755.1 | -1.2 | 8.1E-01 | **-2.3** | **3.1E-06** | -2.1 | 2.0E-02 |
| ARHGAP8 | -1.5 | 2.7E-02 | **-2.3** | **1.3E-03** | -1.7 | 9.7E-03 |
| BHLHA15 | -1.3 | 9.9E-01 | **-2.3** | **8.5E-04** | -2.0 | 9.4E-03 |
| BOK | -1.3 | 5.1E-01 | **-2.3** | **1.0E-04** | -2.0 | 2.4E-04 |
| CNNM1 | -1.6 | 1.3E-01 | **-2.3** | **2.2E-02** | -1.6 | 9.5E-03 |
| CRLF2 | -1.3 | 9.1E-01 | **-2.3** | **1.9E-02** | -1.6 | 3.3E-01 |
| DSCC1 | -1.4 | 3.6E-01 | **-2.3** | **4.0E-05** | -1.2 | 4.8E-01 |
| EPSTI1 | -2.1 | 1.3E-01 | **-2.3** | **1.0E-02** | -1.7 | 1.2E-01 |
| IGHG2 | -1.0 | 5.1E-01 | **-2.3** | **9.2E-04** | -2.3 | 1.4E-06 |
| IGHV3-66 | -1.4 | 1.2E-01 | **-2.3** | **2.3E-03** | -2.3 | 2.5E-05 |
| IGHV4-31 | -1.6 | 3.8E-01 | **-2.3** | **3.6E-07** | -2.4 | 3.5E-06 |
| LY6E | -1.9 | 1.7E-01 | **-2.3** | **4.8E-03** | -1.8 | 5.5E-02 |
| MET | -1.8 | 9.6E-01 | **-2.3** | **1.5E-02** | -2.8 | 5.5E-03 |
| PTPRG | -1.0 | 8.9E-01 | **-2.3** | **3.2E-11** | -2.0 | 3.0E-04 |
| SEPTIN4 | -1.7 | 3.8E-01 | **-2.3** | **4.9E-02** | -1.9 | 1.6E-01 |
| SPATS2L | -2.0 | 1.5E-01 | **-2.3** | **2.1E-02** | -2.0 | 7.4E-02 |
| **TRBV12-5** | -1.0 | 5.2E-01 | **-2.3** | **2.5E-04** | -1.3 | 1.1E-01 |
| TSHR | -1.6 | 4.5E-01 | **-2.3** | **2.2E-03** | -2.4 | 1.9E-03 |
| TXNDC5 | -1.3 | 9.3E-01 | **-2.3** | **3.4E-11** | -1.8 | 2.7E-04 |
| CHAD | -1.2 | 5.3E-01 | **-2.2** | **9.6E-04** | -2.4 | 2.0E-03 |
| COLGALT2 | -1.3 | 3.0E-02 | **-2.2** | **1.5E-12** | -2.3 | 5.9E-29 |
| DNASE1L3 | -1.2 | 8.0E-01 | **-2.2** | **7.5E-04** | -1.7 | 2.1E-02 |
| FBXO39 | -1.7 | 8.7E-02 | **-2.2** | **4.4E-03** | -2.1 | 1.0E-02 |
| FOSB | -1.5 | 9.7E-02 | **-2.2** | **3.9E-09** | -2.4 | 1.5E-10 |
| FZD7 | -1.2 | 8.4E-01 | **-2.2** | **2.6E-02** | -1.7 | 3.4E-02 |
| HRH4 | -1.5 | 1.1E-04 | **-2.2** | **3.0E-05** | -2.4 | 1.2E-04 |
| HRK | -1.8 | 5.7E-05 | **-2.2** | **1.2E-06** | -2.5 | 7.1E-06 |
| IGHV1-3 | -1.1 | 6.8E-01 | **-2.2** | **9.5E-07** | -1.8 | 5.8E-05 |
| IGKV1-12 | 1.0 | 9.2E-01 | **-2.2** | **1.6E-04** | -1.6 | 2.5E-01 |
| IGKV1D-39 | -1.3 | 8.2E-01 | **-2.2** | **1.4E-02** | -1.9 | 9.4E-02 |
| IGKV2D-29 | -1.2 | 4.6E-01 | **-2.2** | **1.8E-03** | -2.6 | 4.3E-12 |
| IGLV5-45 | -1.0 | 5.4E-01 | **-2.2** | **1.5E-02** | -1.4 | 2.4E-01 |
| IGLV8-61 | -1.2 | 9.5E-01 | **-2.2** | **1.8E-02** | -3.1 | 6.3E-05 |
| KIR3DL2 | -1.4 | 2.0E-02 | **-2.2** | **3.3E-09** | -2.2 | 7.4E-08 |
| LRRC43 | -1.4 | 2.1E-01 | **-2.2** | **3.5E-04** | -2.0 | 1.3E-03 |
| LURAP1 | -1.5 | 1.6E-01 | **-2.2** | **1.1E-03** | -1.4 | 3.1E-01 |
| **OAS2** | -1.9 | 8.9E-02 | **-2.2** | **5.1E-03** | -1.9 | 3.2E-02 |
| PTPRB | -1.4 | 7.5E-02 | **-2.2** | **1.0E-03** | -1.9 | 1.0E-03 |
| SORBS2 | -1.4 | 2.7E-01 | **-2.2** | **4.9E-04** | -2.5 | 2.9E-03 |
| TERT | -1.6 | 1.2E-01 | **-2.2** | **7.6E-03** | -2.0 | 4.8E-04 |
| THSD7A | -1.2 | 2.6E-01 | **-2.2** | **5.1E-04** | -4.3 | 3.4E-18 |
| **TRBJ2-6** | -1.2 | 3.6E-01 | **-2.2** | **2.0E-03** | -1.6 | 9.6E-02 |
| **TRBV11-3** | -1.4 | 4.0E-01 | **-2.2** | **3.3E-03** | -1.3 | 3.0E-01 |
| TRO | -1.7 | 6.1E-03 | **-2.2** | **6.4E-04** | -1.8 | 1.9E-03 |
| TTLL7 | -1.3 | 6.5E-02 | **-2.2** | **5.3E-06** | -2.3 | 1.2E-03 |
| ZNF541 | -1.6 | 1.6E-03 | **-2.2** | **3.9E-06** | -1.3 | 1.5E-01 |
| BMP8A | -1.1 | 7.3E-01 | **-2.1** | **1.5E-02** | -1.4 | 5.8E-01 |
| **CA3** | 1.0 | 8.7E-01 | **-2.1** | **7.7E-06** | -1.4 | 1.5E-01 |
| CADM2 | -1.5 | 4.8E-01 | **-2.1** | **3.3E-02** | -1.7 | 2.4E-01 |
| CDC25A | -1.3 | 8.6E-01 | **-2.1** | **4.3E-03** | -1.6 | 2.3E-01 |
| CPA3 | -1.1 | 9.1E-01 | **-2.1** | **9.9E-05** | -5.1 | 1.0E-20 |
| CPNE7 | -1.1 | 9.3E-01 | **-2.1** | **6.0E-03** | -2.0 | 5.8E-05 |
| CTAGE8 | -1.3 | 2.7E-01 | **-2.1** | **2.1E-05** | -1.3 | 2.1E-01 |
| IGHV3-21 | -1.3 | 7.9E-01 | **-2.1** | **9.5E-05** | -2.2 | 2.8E-05 |
| IGHV3-23 | 1.1 | 4.9E-01 | **-2.1** | **4.1E-09** | -2.2 | 2.6E-06 |
| IGHV4-39 | -1.7 | 1.3E-01 | **-2.1** | **3.2E-04** | -2.2 | 2.6E-04 |
| IGHV4-59 | 1.1 | 3.7E-01 | **-2.1** | **9.9E-08** | -2.2 | 7.7E-08 |
| IGHV7-4-1 | -1.1 | 8.6E-01 | **-2.1** | **6.0E-04** | -2.6 | 2.8E-06 |
| IGLV2-11 | -1.3 | 7.4E-01 | **-2.1** | **3.0E-04** | -2.4 | 3.7E-08 |
| IGLV2-8 | -1.1 | 9.2E-01 | **-2.1** | **2.4E-06** | -1.9 | 2.8E-02 |
| IRF6 | -1.6 | 1.3E-05 | **-2.1** | **1.2E-08** | -1.4 | 3.6E-03 |
| KCNK17 | -1.6 | 5.2E-01 | **-2.1** | **6.5E-04** | -1.8 | 3.0E-02 |
| KIR2DL1 | -1.3 | 3.4E-02 | **-2.1** | **1.0E-10** | -2.0 | 1.2E-09 |
| NUAK1 | -1.6 | 5.6E-03 | **-2.1** | **6.8E-14** | -2.3 | 3.4E-10 |
| **OAS1** | -2.0 | 1.5E-01 | **-2.1** | **4.1E-02** | -1.8 | 1.2E-01 |
| OLIG2 | -1.9 | 4.0E-03 | **-2.1** | **1.1E-02** | -1.7 | 8.5E-02 |
| PTGER3 | -1.1 | 5.4E-01 | **-2.1** | **1.8E-03** | -3.7 | 5.6E-16 |
| SCUBE1 | 1.0 | 7.9E-01 | **-2.1** | **1.6E-03** | -1.8 | 5.4E-03 |
| **TNFRSF17** | -1.1 | 7.1E-01 | **-2.1** | **4.8E-05** | -1.4 | 4.4E-02 |
| ZMAT4 | -1.3 | 1.3E-02 | **-2.1** | **2.3E-21** | -1.9 | 1.3E-04 |
| AXL | -1.5 | 2.3E-01 | **-2.0** | **1.1E-02** | -2.2 | 3.4E-04 |
| CDC20 | -1.4 | 1.0E+00 | **-2.0** | **5.3E-03** | -1.6 | 1.6E-01 |
| COL13A1 | -1.5 | 4.0E-02 | **-2.0** | **5.4E-05** | -2.3 | 4.7E-09 |
| CYP4F22 | -1.6 | 9.3E-05 | **-2.0** | **4.2E-07** | -1.7 | 6.3E-08 |
| EPOP | -1.3 | 2.3E-02 | **-2.0** | **5.6E-08** | -1.7 | 2.0E-03 |
| FAM72B | -1.1 | 8.9E-01 | **-2.0** | **1.4E-02** | -1.2 | 7.4E-01 |
| FPR3 | -1.7 | 1.7E-02 | **-2.0** | **9.6E-05** | -1.7 | 9.3E-02 |
| GLB1L2 | -1.3 | 1.0E-02 | **-2.0** | **5.0E-04** | -1.8 | 9.1E-22 |
| HIST1H3G | -1.7 | 5.8E-01 | **-2.0** | **1.1E-02** | -1.3 | 4.1E-01 |
| HIST1H3J | -1.5 | 5.0E-01 | **-2.0** | **1.9E-03** | -1.4 | 2.7E-01 |
| IGHV1-2 | -1.2 | 5.8E-01 | **-2.0** | **8.8E-06** | -1.4 | 4.7E-01 |
| IGHV3-74 | -1.0 | 8.6E-01 | **-2.0** | **3.7E-04** | -1.9 | 5.8E-06 |
| IGHV4-34 | -1.1 | 9.8E-01 | **-2.0** | **8.1E-11** | -1.6 | 4.9E-06 |
| IGKV1-33 | -1.5 | 2.3E-01 | **-2.0** | **3.7E-03** | -2.4 | 9.7E-02 |
| IGKV2-30 | 1.1 | 9.3E-01 | **-2.0** | **7.4E-03** | -2.0 | 3.6E-03 |
| IGKV3D-20 | -1.5 | 7.5E-01 | **-2.0** | **1.5E-03** | -2.3 | 7.0E-04 |
| KIF26B | -1.2 | 1.1E-01 | **-2.0** | **1.9E-05** | -1.7 | 1.4E-03 |
| LMCD1 | -1.2 | 5.0E-01 | **-2.0** | **3.6E-02** | -1.5 | 2.6E-01 |
| LRP1B | 1.0 | 9.1E-01 | **-2.0** | **1.8E-02** | -1.6 | 1.1E-01 |
| NPTX1 | -1.9 | 1.7E-07 | **-2.0** | **2.1E-03** | -2.5 | 3.0E-05 |
| NR4A3 | -1.6 | 7.0E-02 | **-2.0** | **6.0E-03** | -1.9 | 3.2E-04 |
| NYAP1 | -1.4 | 1.5E-01 | **-2.0** | **1.9E-04** | -1.9 | 2.0E-03 |
| PRICKLE2 | -1.2 | 2.4E-01 | **-2.0** | **9.5E-04** | -1.6 | 8.6E-03 |
| PTGDR2 | -1.7 | 1.4E-07 | **-2.0** | **1.2E-03** | -1.8 | 2.2E-02 |
| PYCR1 | -1.2 | 7.8E-01 | **-2.0** | **1.8E-02** | -1.5 | 9.2E-02 |
| RDM1 | 1.0 | 7.2E-01 | **-2.0** | **1.1E-02** | -1.7 | 2.4E-02 |
| RYR3 | -1.3 | 4.9E-01 | **-2.0** | **4.0E-04** | -3.7 | 2.7E-08 |
| SDR42E2 | -1.3 | 3.7E-01 | **-2.0** | **3.1E-03** | -2.0 | 6.6E-02 |
| SEMA3G | -1.7 | 1.4E-01 | **-2.0** | **3.5E-02** | -1.9 | 1.3E-02 |
| TMPRSS6 | -1.3 | 7.2E-02 | **-2.0** | **6.4E-05** | -1.5 | 3.9E-02 |
| TOX2 | -1.4 | 3.9E-04 | **-2.0** | **2.1E-08** | -1.9 | 1.3E-09 |
| TRPC3 | -1.7 | 1.2E-02 | **-2.0** | **9.4E-03** | -1.8 | 2.0E-02 |
| TUB | -1.2 | 5.6E-01 | **-2.0** | **6.9E-04** | -1.7 | 6.7E-02 |
| UPK2 | -1.5 | 6.2E-02 | **-2.0** | **4.9E-02** | -1.5 | 2.0E-01 |
| WSCD2 | -1.0 | 8.8E-01 | **-2.0** | **2.8E-03** | -2.3 | 3.1E-03 |
| ZNF683 | -1.3 | 5.4E-03 | **-2.0** | **4.6E-11** | -1.8 | 1.4E-09 |
| MARCH1 | 1.5 | 4.0E-10 | **2.0** | **1.7E-15** | 1.7 | 1.2E-07 |
| AATK | 1.7 | 1.7E-05 | **2.0** | **4.4E-14** | 1.8 | 2.0E-05 |
| AC015871.1 | 1.4 | 6.0E-06 | **2.0** | **3.6E-14** | 1.6 | 2.3E-06 |
| AC018755.2 | 1.7 | 3.6E-02 | **2.0** | **3.2E-03** | 1.8 | 6.7E-03 |
| AC099489.1 | 1.4 | 2.0E-04 | **2.0** | **1.3E-10** | 1.7 | 1.4E-05 |
| AFF2 | 1.4 | 2.3E-03 | **2.0** | **7.2E-16** | 2.0 | 3.0E-17 |
| AP000646.1 | -1.0 | 8.3E-01 | **2.0** | **4.4E-03** | 1.9 | 1.4E-04 |
| ARHGAP15 | 1.6 | 2.5E-18 | **2.0** | **3.7E-31** | 1.7 | 3.9E-23 |
| ARL11 | 1.5 | 7.7E-23 | **2.0** | **0.0E+00** | 1.7 | 1.3E-17 |
| B4GALT5 | 1.6 | 1.9E-09 | **2.0** | **2.6E-28** | 1.8 | 9.0E-16 |
| BBOF1 | 1.0 | 9.6E-01 | **2.0** | **3.5E-05** | 2.2 | 1.0E-05 |
| BCL3 | 1.6 | 3.6E-05 | **2.0** | **3.7E-22** | 1.7 | 1.8E-08 |
| BSG | 1.1 | 8.7E-01 | **2.0** | **2.0E-03** | 2.1 | 1.3E-03 |
| BST1 | 1.6 | 7.4E-17 | **2.0** | **0.0E+00** | 1.8 | 6.0E-23 |
| BTNL3 | 1.5 | 2.4E-08 | **2.0** | **7.0E-11** | 1.8 | 7.4E-06 |
| C1RL | 1.5 | 8.5E-12 | **2.0** | **0.0E+00** | 1.7 | 1.1E-15 |
| CAPNS2 | 1.3 | 9.5E-03 | **2.0** | **1.5E-09** | 1.6 | 5.3E-06 |
| CKLF-CMTM1 | 1.4 | 1.4E-03 | **2.0** | **1.1E-07** | 1.6 | 2.2E-05 |
| CPEB4 | 1.4 | 2.4E-15 | **2.0** | **0.0E+00** | 1.8 | 3.5E-20 |
| CSF3R | 1.5 | 7.8E-07 | **2.0** | **2.1E-17** | 1.7 | 6.2E-08 |
| CSTA | 1.4 | 2.7E-03 | **2.0** | **1.6E-20** | 2.3 | 3.4E-06 |
| **CXCR1** | 1.6 | 5.6E-08 | **2.0** | **9.1E-19** | 1.6 | 4.0E-06 |
| DPCD | 1.1 | 8.2E-01 | **2.0** | **1.5E-09** | 2.2 | 4.0E-06 |
| DRC1 | 1.5 | 1.5E-01 | **2.0** | **6.1E-05** | 3.1 | 7.2E-06 |
| E2F2 | -1.1 | 7.9E-01 | **2.0** | **2.0E-06** | 2.0 | 9.8E-05 |
| EVI2A | 1.5 | 2.1E-02 | **2.0** | **1.4E-07** | 1.8 | 3.0E-04 |
| EVPL | 1.2 | 7.7E-01 | **2.0** | **1.2E-04** | 2.3 | 7.0E-09 |
| FBXL5 | 1.6 | 1.5E-19 | **2.0** | **0.0E+00** | 1.8 | 8.3E-21 |
| FCGR3B | 1.6 | 1.8E-06 | **2.0** | **1.6E-14** | 1.7 | 1.5E-06 |
| **FOXO4** | 1.2 | 1.9E-01 | **2.0** | **1.8E-08** | 1.9 | 9.3E-10 |
| GJB6 | 1.3 | 3.8E-01 | **2.0** | **3.5E-03** | 2.8 | 1.4E-06 |
| GLRX5 | -1.1 | 8.0E-01 | **2.0** | **3.4E-02** | 2.2 | 6.0E-03 |
| GPAT3 | 1.7 | 6.3E-21 | **2.0** | **1.3E-30** | 1.8 | 1.9E-17 |
| GPER1 | 1.4 | 1.1E-01 | **2.0** | **1.5E-03** | 2.1 | 8.4E-04 |
| HCK | 1.5 | 2.8E-11 | **2.0** | **3.9E-28** | 1.8 | 7.5E-12 |
| **IL27** | 1.4 | 3.4E-01 | **2.0** | **9.1E-03** | 1.8 | 2.6E-02 |
| KCNE1 | 1.3 | 1.2E-02 | **2.0** | **1.0E-11** | 1.7 | 5.3E-08 |
| KIF1B | 1.5 | 3.8E-08 | **2.0** | **3.9E-22** | 1.8 | 7.9E-14 |
| KISS1R | 1.6 | 7.5E-02 | **2.0** | **4.9E-05** | 1.8 | 4.0E-03 |
| LGALS3 | 1.1 | 9.8E-01 | **2.0** | **2.1E-04** | 2.3 | 8.0E-06 |
| LLCFC1 | 1.4 | 2.0E-01 | **2.0** | **4.9E-07** | 1.9 | 6.4E-07 |
| LRRC4 | 1.7 | 4.1E-12 | **2.0** | **2.7E-19** | 1.8 | 2.0E-08 |
| LTBP2 | 1.3 | 1.1E-02 | **2.0** | **7.6E-15** | 1.9 | 2.6E-14 |
| MAK | 1.6 | 2.0E-12 | **2.0** | **2.8E-16** | 1.6 | 1.5E-07 |
| MKRN1 | -1.0 | 7.9E-01 | **2.0** | **4.5E-04** | 2.1 | 4.3E-05 |
| MOSPD2 | 1.6 | 1.1E-17 | **2.0** | **1.9E-26** | 1.8 | 9.4E-15 |
| MYL6 | 1.3 | 6.4E-03 | **2.0** | **7.5E-29** | 2.0 | 7.0E-16 |
| NFIX | 1.0 | 8.9E-01 | **2.0** | **1.4E-02** | 2.2 | 1.8E-03 |
| PDZD3 | 1.4 | 1.7E-01 | **2.0** | **3.3E-10** | 1.8 | 6.2E-04 |
| PFKFB2 | 1.2 | 2.7E-02 | **2.0** | **7.1E-23** | 1.7 | 2.5E-15 |
| PGD | 1.5 | 1.4E-06 | **2.0** | **6.5E-16** | 1.8 | 5.8E-13 |
| PLBD1 | 1.6 | 6.3E-11 | **2.0** | **1.9E-27** | 2.0 | 1.3E-15 |
| SDCBP | 1.6 | 1.2E-14 | **2.0** | **2.5E-20** | 1.7 | 4.6E-12 |
| **SERPINA1** | 1.6 | 3.1E-11 | **2.0** | **5.9E-26** | 1.7 | 6.4E-11 |
| SLC6A9 | 1.0 | 9.4E-01 | **2.0** | **6.7E-03** | 2.1 | 3.9E-03 |
| SNCA | 1.0 | 9.1E-01 | **2.0** | **3.7E-03** | 2.1 | 1.7E-03 |
| ST6GALNAC3 | 1.4 | 9.5E-07 | **2.0** | **3.4E-36** | 1.9 | 1.5E-17 |
| TDP2 | 1.6 | 3.8E-22 | **2.0** | **4.5E-32** | 1.7 | 2.9E-15 |
| TMCO3 | 1.5 | 2.3E-11 | **2.0** | **1.2E-29** | 1.8 | 4.9E-19 |
| TMEM260 | 1.4 | 2.3E-06 | **2.0** | **2.9E-32** | 1.7 | 1.9E-20 |
| **TNFRSF10C** | 1.6 | 6.5E-09 | **2.0** | **3.8E-16** | 1.7 | 3.7E-06 |
| TUBA1A | 1.6 | 3.6E-13 | **2.0** | **7.8E-31** | 1.7 | 1.5E-10 |
| UBE2D1 | 1.6 | 1.6E-09 | **2.0** | **1.2E-23** | 1.7 | 1.1E-04 |
| UBXN2B | 1.5 | 5.9E-23 | **2.0** | **1.1E-34** | 1.7 | 1.2E-15 |
| UPB1 | 1.5 | 1.3E-03 | **2.0** | **6.5E-10** | 1.9 | 6.9E-07 |
| ADGRE2 | 1.6 | 1.0E-09 | **2.1** | **1.7E-22** | 1.9 | 4.4E-13 |
| AIM2 | 1.4 | 1.8E-01 | **2.1** | **2.5E-05** | 2.0 | 1.4E-03 |
| ALOX5 | 1.6 | 3.5E-12 | **2.1** | **0.0E+00** | 1.9 | 1.0E-20 |
| ALOX5AP | 1.6 | 2.0E-09 | **2.1** | **1.3E-14** | 1.8 | 4.7E-09 |
| ALPK1 | 1.5 | 1.7E-07 | **2.1** | **7.3E-28** | 1.9 | 8.3E-11 |
| AQP1 | 1.2 | 6.1E-01 | **2.1** | **3.8E-04** | 2.1 | 1.6E-06 |
| ATP9A | 1.2 | 2.1E-01 | **2.1** | **9.6E-07** | 2.0 | 3.5E-06 |
| BLVRB | 1.2 | 6.6E-01 | **2.1** | **1.0E-04** | 2.2 | 4.7E-09 |
| **CD55** | 1.5 | 6.5E-08 | **2.1** | **0.0E+00** | 1.7 | 7.4E-14 |
| CDA | 1.6 | 1.1E-12 | **2.1** | **4.9E-20** | 1.8 | 1.3E-09 |
| CDK14 | 1.7 | 1.3E-10 | **2.1** | **6.6E-28** | 1.8 | 8.5E-14 |
| **CEACAM3** | 1.5 | 1.5E-10 | **2.1** | **2.1E-23** | 1.7 | 2.5E-09 |
| CEBPB | 1.7 | 7.2E-07 | **2.1** | **8.0E-31** | 2.0 | 4.3E-13 |
| CHSY1 | 1.6 | 1.8E-10 | **2.1** | **4.1E-24** | 1.7 | 1.4E-10 |
| COL9A3 | 1.6 | 4.7E-06 | **2.1** | **4.7E-13** | 1.9 | 1.5E-08 |
| **CXCL1** | 1.6 | 6.1E-07 | **2.1** | **2.7E-18** | 1.8 | 4.0E-08 |
| CYP4F3 | 1.6 | 1.0E-05 | **2.1** | **1.1E-13** | 1.7 | 1.7E-05 |
| DHRS12 | 1.5 | 5.2E-07 | **2.1** | **1.1E-21** | 1.9 | 1.1E-16 |
| DPY19L3 | 1.5 | 2.0E-06 | **2.1** | **4.0E-30** | 1.9 | 3.9E-16 |
| EPHB4 | 1.7 | 1.5E-07 | **2.1** | **1.1E-20** | 1.8 | 3.0E-07 |
| ETS2 | 1.6 | 1.6E-16 | **2.1** | **0.0E+00** | 1.8 | 4.2E-16 |
| FAM126B | 1.6 | 1.7E-15 | **2.1** | **9.2E-36** | 1.7 | 5.1E-11 |
| FAT1 | -1.1 | 6.8E-01 | **2.1** | **9.5E-04** | 2.5 | 2.1E-07 |
| FCER1G | 1.5 | 5.5E-12 | **2.1** | **0.0E+00** | 2.0 | 0.0E+00 |
| FECH | -1.2 | 4.8E-01 | **2.1** | **3.6E-03** | 2.6 | 2.3E-05 |
| FKBP9 | 1.5 | 1.7E-05 | **2.1** | **4.4E-12** | 2.0 | 4.4E-07 |
| FPR1 | 1.6 | 1.3E-08 | **2.1** | **1.3E-28** | 1.8 | 1.1E-10 |
| FRAT2 | 1.7 | 4.0E-11 | **2.1** | **4.6E-27** | 1.8 | 1.3E-10 |
| GABRR2 | 1.4 | 1.0E-04 | **2.1** | **2.1E-11** | 2.0 | 1.7E-15 |
| GLT1D1 | 1.6 | 7.1E-10 | **2.1** | **3.3E-20** | 1.9 | 1.6E-10 |
| GPR160 | 1.4 | 4.6E-06 | **2.1** | **0.0E+00** | 1.9 | 1.0E-11 |
| GYPA | 1.1 | 6.8E-01 | **2.1** | **3.1E-04** | 2.2 | 7.9E-04 |
| HEMGN | 1.2 | 4.7E-01 | **2.1** | **1.1E-06** | 2.3 | 5.7E-08 |
| HEPH | 1.6 | 7.7E-04 | **2.1** | **4.6E-11** | 1.8 | 2.7E-07 |
| HORMAD1 | 1.6 | 7.5E-03 | **2.1** | **5.9E-07** | 1.9 | 1.1E-03 |
| **IFNGR1** | 1.6 | 2.6E-12 | **2.1** | **0.0E+00** | 1.9 | 8.5E-20 |
| IRAK3 | 1.6 | 8.9E-09 | **2.1** | **2.2E-36** | 1.9 | 1.7E-15 |
| KEL | 1.1 | 8.6E-01 | **2.1** | **4.2E-04** | 2.3 | 5.3E-07 |
| LILRA6 | 1.6 | 2.4E-15 | **2.1** | **2.2E-32** | 1.9 | 6.7E-25 |
| LIPN | 1.6 | 1.2E-08 | **2.1** | **1.0E-24** | 1.9 | 9.3E-16 |
| LMNB1 | 1.6 | 9.8E-06 | **2.1** | **1.4E-17** | 2.0 | 8.1E-13 |
| LRRC6 | 1.6 | 3.9E-16 | **2.1** | **2.7E-19** | 1.8 | 1.4E-30 |
| MAPK14 | 1.6 | 2.0E-13 | **2.1** | **0.0E+00** | 1.9 | 3.8E-21 |
| MMP1 | -1.1 | 9.1E-01 | **2.1** | **1.7E-02** | 2.9 | 1.7E-04 |
| MPP1 | 1.2 | 7.5E-01 | **2.1** | **7.7E-06** | 2.2 | 1.0E-06 |
| NABP1 | 1.6 | 3.1E-15 | **2.1** | **0.0E+00** | 1.8 | 9.7E-11 |
| NATD1 | 1.5 | 5.2E-04 | **2.1** | **1.7E-27** | 1.9 | 5.0E-12 |
| NDUFB3 | 1.6 | 2.2E-04 | **2.1** | **6.0E-25** | 2.1 | 1.2E-08 |
| NFIL3 | 1.6 | 8.0E-07 | **2.1** | **4.4E-16** | 1.8 | 7.4E-08 |
| NOS1AP | 1.3 | 3.4E-01 | **2.1** | **8.8E-04** | 1.6 | 3.6E-02 |
| NPL | 1.6 | 3.6E-13 | **2.1** | **2.5E-32** | 1.8 | 8.1E-13 |
| NRBF2 | 1.6 | 4.6E-17 | **2.1** | **4.1E-24** | 1.7 | 2.1E-10 |
| PHTF1 | 1.4 | 1.3E-05 | **2.1** | **9.0E-35** | 1.9 | 1.1E-21 |
| PLSCR4 | 1.1 | 7.2E-01 | **2.1** | **2.7E-06** | 2.0 | 2.6E-03 |
| PLXNC1 | 1.6 | 2.9E-12 | **2.1** | **5.1E-31** | 1.9 | 2.3E-12 |
| PTPRF | 1.1 | 7.6E-01 | **2.1** | **5.6E-05** | 2.5 | 5.1E-08 |
| RGS2 | 1.7 | 2.2E-11 | **2.1** | **1.3E-19** | 1.8 | 5.2E-13 |
| RHAG | 1.2 | 7.5E-01 | **2.1** | **1.4E-03** | 2.2 | 2.7E-04 |
| **SERPINB1** | 1.5 | 3.2E-07 | **2.1** | **9.6E-32** | 1.9 | 2.1E-16 |
| SFRP2 | 1.1 | 8.7E-01 | **2.1** | **1.2E-05** | 2.1 | 7.0E-06 |
| **SIGLEC5** | 1.8 | 8.0E-16 | **2.1** | **1.0E-14** | 1.8 | 7.4E-07 |
| **SIGLEC9** | 1.6 | 7.1E-10 | **2.1** | **5.1E-36** | 1.8 | 4.8E-12 |
| SLC25A37 | 1.2 | 6.9E-01 | **2.1** | **7.0E-05** | 2.2 | 1.2E-05 |
| SLC8A1 | 1.6 | 2.2E-19 | **2.1** | **6.0E-26** | 2.0 | 1.4E-22 |
| TENT5C | -1.1 | 6.5E-01 | **2.1** | **1.6E-02** | 2.5 | 6.0E-04 |
| **TLR8** | 1.7 | 2.8E-20 | **2.1** | **1.4E-36** | 1.9 | 2.7E-15 |
| TMEM119 | 1.5 | 1.6E-02 | **2.1** | **2.5E-10** | 1.9 | 1.5E-04 |
| TMEM121B | 1.6 | 8.5E-11 | **2.1** | **2.5E-24** | 2.0 | 7.6E-13 |
| TNNI2 | 1.5 | 1.3E-03 | **2.1** | **1.3E-07** | 1.9 | 5.6E-09 |
| UBE2O | 1.1 | 9.9E-01 | **2.1** | **3.3E-04** | 2.4 | 5.6E-07 |
| USB1 | 1.6 | 3.3E-15 | **2.1** | **7.9E-34** | 1.8 | 1.9E-20 |
| APMAP | 1.7 | 2.5E-08 | **2.2** | **1.8E-28** | 2.0 | 5.0E-17 |
| C4BPA | 1.8 | 6.4E-02 | **2.2** | **1.4E-02** | 1.5 | 8.1E-02 |
| C8orf88 | 1.5 | 4.8E-03 | **2.2** | **2.3E-12** | 1.9 | 2.4E-20 |
| CASP4 | 1.6 | 2.3E-17 | **2.2** | **2.5E-26** | 1.9 | 1.3E-16 |
| CD300LD | 1.7 | 2.6E-03 | **2.2** | **6.1E-13** | 1.6 | 3.1E-05 |
| CLCN1 | 1.3 | 1.8E-01 | **2.2** | **2.8E-04** | 1.5 | 5.9E-02 |
| CNTNAP3 | 1.5 | 3.9E-03 | **2.2** | **9.4E-10** | 1.8 | 1.1E-04 |
| CNTNAP3B | 1.4 | 2.2E-01 | **2.2** | **9.0E-04** | 1.5 | 1.3E-01 |
| **CXCL6** | 1.6 | 6.7E-02 | **2.2** | **1.1E-04** | 1.3 | 1.2E-01 |
| DOK3 | 1.7 | 9.5E-10 | **2.2** | **2.7E-34** | 1.9 | 1.7E-12 |
| DYRK3 | -1.1 | 7.0E-01 | **2.2** | **2.7E-04** | 2.5 | 1.4E-04 |
| FCGR2A | 1.7 | 3.5E-13 | **2.2** | **9.7E-35** | 1.9 | 2.8E-11 |
| FLOT1 | 1.6 | 6.7E-15 | **2.2** | **0.0E+00** | 1.9 | 9.2E-27 |
| FPR2 | 1.7 | 2.7E-08 | **2.2** | **1.9E-21** | 1.8 | 9.5E-09 |
| FUT7 | 1.7 | 4.6E-10 | **2.2** | **2.3E-36** | 1.9 | 3.9E-16 |
| GMPR | -1.0 | 7.3E-01 | **2.2** | **4.6E-02** | 2.4 | 1.3E-02 |
| HAUS4 | 1.5 | 6.1E-08 | **2.2** | **1.1E-23** | 1.9 | 6.1E-16 |
| HSPA6 | 1.7 | 2.1E-11 | **2.2** | **8.5E-21** | 1.9 | 7.7E-11 |
| **IL1B** | 1.6 | 8.4E-06 | **2.2** | **3.5E-15** | 1.7 | 1.8E-06 |
| KIAA0319 | 1.7 | 1.1E-06 | **2.2** | **3.3E-15** | 2.0 | 4.4E-07 |
| LIMK2 | 1.6 | 2.5E-07 | **2.2** | **1.2E-21** | 1.8 | 1.4E-09 |
| MKNK1 | 1.5 | 6.7E-11 | **2.2** | **1.2E-33** | 1.9 | 1.5E-18 |
| MMP25 | 1.7 | 5.0E-07 | **2.2** | **6.3E-19** | 2.0 | 1.0E-10 |
| MPO | 1.0 | 8.1E-01 | **2.2** | **2.4E-08** | 2.9 | 5.9E-08 |
| MXD3 | 1.6 | 7.8E-09 | **2.2** | **4.6E-31** | 1.8 | 1.4E-14 |
| MYLK3 | 1.6 | 8.1E-03 | **2.2** | **2.6E-07** | 2.0 | 1.8E-05 |
| NRN1 | 1.4 | 2.4E-01 | **2.2** | **1.4E-04** | 2.0 | 2.1E-03 |
| OR2W3 | -1.0 | 6.5E-01 | **2.2** | **1.0E-02** | 2.5 | 7.9E-04 |
| OTX1 | 1.7 | 1.0E-04 | **2.2** | **1.3E-09** | 1.9 | 3.9E-08 |
| PADI2 | 1.7 | 1.9E-13 | **2.2** | **5.3E-19** | 2.0 | 1.1E-12 |
| **PADI4** | 1.6 | 3.3E-07 | **2.2** | **1.7E-14** | 1.9 | 3.8E-11 |
| PITHD1 | -1.1 | 7.5E-01 | **2.2** | **1.1E-02** | 2.3 | 1.0E-02 |
| PLEK2 | 1.1 | 9.5E-01 | **2.2** | **2.0E-04** | 2.4 | 7.4E-08 |
| PRDM5 | 1.5 | 1.1E-04 | **2.2** | **6.8E-13** | 2.1 | 6.3E-09 |
| RAB20 | 1.6 | 1.1E-06 | **2.2** | **2.3E-13** | 1.8 | 2.5E-07 |
| RALB | 1.6 | 2.9E-15 | **2.2** | **2.7E-28** | 1.9 | 5.9E-15 |
| SAMSN1 | 1.4 | 2.1E-08 | **2.2** | **6.5E-18** | 1.9 | 7.9E-15 |
| SEPTIN5 | 1.2 | 4.3E-01 | **2.2** | **8.7E-04** | 2.2 | 1.6E-04 |
| SLC2A14 | 1.7 | 8.5E-02 | **2.2** | **3.3E-04** | 2.3 | 1.9E-04 |
| SLC6A8 | 1.1 | 8.9E-01 | **2.2** | **5.2E-03** | 2.3 | 2.1E-03 |
| TDRD9 | 1.4 | 3.3E-05 | **2.2** | **4.0E-20** | 2.1 | 1.8E-15 |
| TECPR2 | 1.7 | 6.6E-09 | **2.2** | **1.1E-27** | 1.9 | 2.3E-10 |
| TG | 1.7 | 1.3E-10 | **2.2** | **1.3E-30** | 1.8 | 1.0E-12 |
| **TLR4** | 1.7 | 6.2E-15 | **2.2** | **0.0E+00** | 1.9 | 9.3E-20 |
| **TLR6** | 1.7 | 1.9E-20 | **2.2** | **1.1E-29** | 1.9 | 1.2E-11 |
| TMC5 | 1.3 | 5.3E-01 | **2.2** | **9.3E-04** | 2.5 | 2.2E-04 |
| TMCC2 | 1.0 | 8.8E-01 | **2.2** | **6.5E-03** | 2.4 | 1.1E-03 |
| TNS1 | 1.1 | 9.6E-01 | **2.2** | **1.7E-03** | 2.3 | 7.0E-04 |
| TRIM58 | -1.1 | 6.7E-01 | **2.2** | **1.7E-02** | 2.5 | 1.1E-03 |
| TSPAN7 | 1.0 | 9.1E-01 | **2.2** | **2.4E-05** | 2.1 | 5.5E-04 |
| A3GALT2 | 1.6 | 7.8E-03 | **2.3** | **6.1E-11** | 1.9 | 5.0E-05 |
| ACKR1 | 1.2 | 5.5E-01 | **2.3** | **7.4E-04** | 2.4 | 4.8E-05 |
| AIF1 | 1.7 | 1.0E-08 | **2.3** | **9.0E-24** | 2.3 | 5.9E-11 |
| AL162377.3 | 1.5 | 2.6E-06 | **2.3** | **2.2E-22** | 1.9 | 1.8E-09 |
| ANKRD34B | 1.7 | 1.0E-04 | **2.3** | **2.7E-20** | 2.1 | 3.6E-11 |
| ARHGEF37 | 1.2 | 4.6E-01 | **2.3** | **3.1E-06** | 2.2 | 1.1E-08 |
| BMP2 | 1.4 | 1.9E-01 | **2.3** | **6.7E-05** | 2.4 | 2.3E-03 |
| BTNL8 | 1.7 | 4.2E-14 | **2.3** | **3.8E-28** | 1.9 | 2.3E-12 |
| C1orf226 | 1.1 | 7.1E-01 | **2.3** | **1.6E-03** | 1.9 | 9.7E-03 |
| C5orf47 | 2.1 | 6.7E-02 | **2.3** | **2.7E-03** | 1.3 | 5.8E-01 |
| CCDC71L | 1.6 | 4.7E-07 | **2.3** | **2.7E-34** | 2.0 | 4.1E-14 |
| CYP26B1 | 1.5 | 1.0E-01 | **2.3** | **2.3E-03** | 2.1 | 5.4E-02 |
| FLOT2 | 1.7 | 2.2E-12 | **2.3** | **5.0E-35** | 2.0 | 3.7E-17 |
| GADD45A | 1.3 | 1.7E-01 | **2.3** | **2.9E-15** | 2.3 | 2.8E-19 |
| GPR27 | 1.8 | 6.7E-08 | **2.3** | **1.6E-23** | 1.9 | 1.7E-10 |
| HCAR2 | 1.7 | 1.7E-10 | **2.3** | **2.5E-28** | 1.8 | 3.4E-10 |
| KCNJ2 | 1.7 | 1.4E-09 | **2.3** | **3.1E-18** | 2.0 | 1.4E-07 |
| KLC3 | -1.0 | 7.4E-01 | **2.3** | **1.9E-02** | 2.4 | 2.2E-03 |
| LRRN1 | 1.6 | 2.6E-06 | **2.3** | **5.4E-16** | 2.2 | 5.2E-15 |
| MARCKS | 1.8 | 2.1E-15 | **2.3** | **1.1E-24** | 2.0 | 3.8E-09 |
| MBOAT2 | 1.5 | 9.1E-12 | **2.3** | **0.0E+00** | 2.1 | 5.9E-22 |
| MYL4 | 1.1 | 9.9E-01 | **2.3** | **4.2E-04** | 2.2 | 1.5E-05 |
| NCF4 | 1.6 | 8.1E-14 | **2.3** | **7.2E-35** | 1.9 | 5.1E-16 |
| NFE2 | 1.6 | 1.0E-06 | **2.3** | **3.4E-33** | 2.0 | 4.9E-21 |
| **NLRP6** | 1.6 | 8.5E-06 | **2.3** | **2.1E-19** | 1.9 | 1.2E-07 |
| NQO2 | 1.6 | 3.2E-10 | **2.3** | **2.3E-31** | 2.0 | 1.6E-16 |
| P4HA2 | 1.7 | 7.6E-05 | **2.3** | **2.1E-15** | 2.2 | 4.9E-08 |
| PI3 | 1.7 | 4.1E-03 | **2.3** | **3.8E-11** | 2.0 | 3.8E-05 |
| PRRT4 | 1.2 | 1.2E-01 | **2.3** | **3.1E-05** | 3.2 | 6.0E-09 |
| SAMD15 | 1.6 | 1.4E-01 | **2.3** | **4.7E-04** | 2.3 | 1.0E-04 |
| SLC11A1 | 1.7 | 5.2E-08 | **2.3** | **1.9E-30** | 2.0 | 1.7E-15 |
| SLC12A1 | 5.4 | 1.1E-01 | **2.3** | **3.6E-05** | 4.0 | 7.8E-06 |
| TLR1 | 1.7 | 1.6E-18 | **2.3** | **7.0E-36** | 2.0 | 4.0E-16 |
| TMOD1 | 1.0 | 9.5E-01 | **2.3** | **1.0E-03** | 2.5 | 6.1E-07 |
| TRIM10 | -1.0 | 9.1E-01 | **2.3** | **4.9E-05** | 2.4 | 9.1E-06 |
| ANK1 | 1.1 | 9.6E-01 | **2.4** | **3.0E-04** | 2.6 | 5.1E-06 |
| ASPH | 1.7 | 4.8E-12 | **2.4** | **4.5E-20** | 2.1 | 6.7E-10 |
| B3GNT5 | 1.7 | 2.5E-13 | **2.4** | **1.2E-28** | 2.3 | 2.4E-23 |
| BEND7 | 1.7 | 1.6E-05 | **2.4** | **2.0E-19** | 2.0 | 7.6E-07 |
| C17orf99 | 1.2 | 9.9E-01 | **2.4** | **1.6E-03** | 2.3 | 4.3E-02 |
| CCNJL | 1.9 | 3.0E-10 | **2.4** | **5.8E-19** | 2.0 | 4.3E-08 |
| GK | 1.6 | 2.9E-08 | **2.4** | **1.1E-29** | 1.9 | 5.1E-08 |
| GYPB | 1.3 | 1.4E-01 | **2.4** | **5.4E-05** | 2.3 | 4.0E-05 |
| HCAR3 | 1.9 | 2.9E-09 | **2.4** | **4.6E-29** | 2.1 | 5.6E-13 |
| HEPACAM2 | 1.2 | 9.0E-01 | **2.4** | **5.0E-04** | 2.4 | 5.4E-04 |
| HRH2 | 1.7 | 1.6E-08 | **2.4** | **2.8E-19** | 2.1 | 2.9E-10 |
| **IL1R2** | 1.6 | 1.0E-06 | **2.4** | **7.9E-34** | 1.9 | 4.4E-09 |
| IL4R | 1.7 | 4.2E-09 | **2.4** | **4.9E-27** | 2.2 | 4.8E-19 |
| KLF1 | 1.2 | 6.5E-01 | **2.4** | **1.7E-04** | 2.5 | 8.4E-05 |
| LSMEM1 | 1.8 | 7.6E-13 | **2.4** | **4.8E-22** | 2.0 | 4.3E-16 |
| MCTP2 | 1.7 | 2.9E-10 | **2.4** | **6.7E-31** | 2.0 | 6.8E-14 |
| MRC2 | 1.2 | 4.7E-01 | **2.4** | **1.2E-08** | 2.3 | 3.6E-10 |
| NT5DC4 | 1.7 | 1.8E-04 | **2.4** | **3.2E-10** | 2.1 | 2.6E-09 |
| OLAH | 1.4 | 1.2E-01 | **2.4** | **3.4E-04** | 1.9 | 1.0E-02 |
| POU5F1 | 1.8 | 5.1E-09 | **2.4** | **4.6E-08** | 1.9 | 2.1E-07 |
| SHOX2 | 1.6 | 7.2E-02 | **2.4** | **1.1E-05** | 2.4 | 2.9E-09 |
| SIRPD | 1.6 | 2.5E-09 | **2.4** | **0.0E+00** | 2.1 | 6.4E-15 |
| SRGN | 1.6 | 4.9E-09 | **2.4** | **0.0E+00** | 2.1 | 4.0E-12 |
| SRPK1 | 1.7 | 2.1E-18 | **2.4** | **0.0E+00** | 2.1 | 4.6E-29 |
| TMTC1 | 1.6 | 1.9E-09 | **2.4** | **9.6E-12** | 2.2 | 2.2E-09 |
| TP53I11 | 1.7 | 4.1E-09 | **2.4** | **4.4E-29** | 2.0 | 2.2E-14 |
| TXN | 1.6 | 2.0E-06 | **2.4** | **4.6E-28** | 2.4 | 5.7E-27 |
| WDFY3 | 1.7 | 2.1E-10 | **2.4** | **1.7E-25** | 2.0 | 5.2E-11 |
| XK | 1.1 | 9.7E-01 | **2.4** | **4.5E-05** | 3.0 | 1.8E-07 |
| XKR3 | 1.8 | 9.8E-04 | **2.4** | **7.5E-07** | 1.7 | 5.6E-04 |
| ALDH1A2 | 1.8 | 1.5E-05 | **2.5** | **2.1E-22** | 2.1 | 9.3E-08 |
| BPGM | 1.0 | 9.8E-01 | **2.5** | **2.2E-03** | 3.0 | 1.0E-03 |
| CREB5 | 1.8 | 1.5E-24 | **2.5** | **0.0E+00** | 2.0 | 5.5E-14 |
| CST7 | 1.7 | 7.0E-05 | **2.5** | **3.2E-35** | 2.2 | 8.8E-17 |
| FBXL13 | 1.9 | 6.2E-09 | **2.5** | **2.2E-18** | 2.0 | 8.5E-08 |
| GRB10 | 1.4 | 1.2E-02 | **2.5** | **1.1E-21** | 2.2 | 1.7E-12 |
| IL18RAP | 1.7 | 2.0E-09 | **2.5** | **1.5E-22** | 2.0 | 5.0E-19 |
| IL1R1 | 1.6 | 4.1E-06 | **2.5** | **6.9E-21** | 2.1 | 4.6E-08 |
| KANK2 | 1.1 | 7.8E-01 | **2.5** | **2.6E-09** | 2.8 | 4.8E-09 |
| LIN7A | 1.8 | 7.7E-23 | **2.5** | **0.0E+00** | 2.2 | 3.9E-17 |
| NIBAN1 | 1.8 | 3.3E-12 | **2.5** | **1.1E-25** | 2.2 | 2.0E-11 |
| PLAU | 1.7 | 1.3E-03 | **2.5** | **1.0E-11** | 2.3 | 4.4E-07 |
| QPCT | 1.9 | 2.9E-09 | **2.5** | **2.1E-22** | 2.1 | 7.0E-13 |
| RHCE | 1.3 | 2.8E-01 | **2.5** | **1.4E-06** | 2.5 | 2.4E-06 |
| ROPN1L | 1.8 | 6.2E-12 | **2.5** | **5.0E-29** | 2.0 | 1.2E-14 |
| TGM2 | 1.2 | 6.7E-01 | **2.5** | **4.3E-05** | 2.8 | 3.8E-08 |
| TRPM6 | 1.8 | 8.9E-09 | **2.5** | **6.0E-24** | 2.2 | 5.1E-12 |
| UBB | 1.2 | 9.4E-01 | **2.5** | **1.4E-03** | 2.7 | 9.6E-05 |
| ZNF254 | 1.7 | 1.9E-06 | **2.5** | **1.6E-19** | 2.4 | 2.4E-17 |
| ZNF438 | 1.7 | 1.9E-13 | **2.5** | **0.0E+00** | 2.1 | 5.6E-15 |
| AZU1 | -1.1 | 9.8E-01 | **2.6** | **7.8E-09** | 3.8 | 1.8E-07 |
| CA12 | 2.0 | 1.6E-01 | **2.6** | **4.1E-05** | 2.2 | 1.4E-02 |
| DGAT2 | 1.9 | 4.8E-09 | **2.6** | **1.3E-25** | 2.3 | 6.0E-11 |
| DUSP13 | 1.8 | 1.7E-01 | **2.6** | **5.3E-05** | 2.6 | 1.4E-04 |
| DYSF | 1.8 | 1.4E-09 | **2.6** | **2.3E-26** | 2.3 | 3.3E-16 |
| FCAR | 1.8 | 7.0E-23 | **2.6** | **0.0E+00** | 2.3 | 8.1E-23 |
| FCGR1A | 1.5 | 2.1E-01 | **2.6** | **2.7E-06** | 2.6 | 4.8E-05 |
| FGF13 | 1.6 | 1.2E-03 | **2.6** | **3.4E-08** | 2.6 | 2.2E-07 |
| GYG1 | 1.6 | 5.2E-06 | **2.6** | **1.4E-33** | 2.4 | 1.2E-34 |
| IFIT1B | 1.0 | 9.6E-01 | **2.6** | **2.8E-04** | 3.1 | 2.1E-04 |
| KIAA0825 | 1.8 | 5.9E-19 | **2.6** | **2.9E-31** | 2.3 | 1.2E-15 |
| NFE4 | 1.8 | 1.1E-11 | **2.6** | **5.5E-30** | 2.1 | 6.5E-15 |
| OMG | 1.8 | 5.6E-05 | **2.6** | **1.9E-09** | 2.1 | 1.8E-05 |
| PGS1 | 1.7 | 1.5E-09 | **2.6** | **0.0E+00** | 2.2 | 3.2E-25 |
| RAB13 | 1.4 | 7.0E-01 | **2.6** | **2.0E-02** | 3.4 | 6.5E-03 |
| RAB3IL1 | 1.3 | 6.8E-01 | **2.6** | **2.3E-05** | 2.6 | 1.7E-06 |
| SEC14L4 | 1.3 | 4.0E-01 | **2.6** | **1.9E-06** | 2.5 | 1.2E-08 |
| SHISA7 | 1.4 | 2.6E-01 | **2.6** | **4.9E-04** | 2.9 | 2.7E-05 |
| SLC37A3 | 1.7 | 1.9E-06 | **2.6** | **3.2E-28** | 2.3 | 2.1E-19 |
| SPINK8 | 1.5 | 3.4E-01 | **2.6** | **2.9E-03** | 2.5 | 3.6E-03 |
| SULT1B1 | 1.9 | 1.3E-13 | **2.6** | **1.3E-25** | 2.2 | 8.1E-12 |
| TGFA | 1.9 | 1.1E-11 | **2.6** | **9.6E-25** | 2.0 | 1.2E-09 |
| VNN2 | 1.9 | 3.4E-11 | **2.6** | **5.4E-22** | 2.3 | 6.0E-12 |
| APOBEC2 | 1.1 | 8.2E-01 | **2.7** | **5.1E-03** | 3.3 | 2.3E-04 |
| CTSE | 1.3 | 3.4E-01 | **2.7** | **2.1E-07** | 2.9 | 1.8E-12 |
| LOXL1 | 1.7 | 4.1E-04 | **2.7** | **1.0E-19** | 2.4 | 1.0E-12 |
| MAP3K20 | 1.8 | 1.6E-15 | **2.7** | **0.0E+00** | 2.4 | 0.0E+00 |
| OOSP3 | 1.8 | 2.4E-03 | **2.7** | **2.2E-11** | 1.9 | 4.5E-05 |
| RHD | 1.3 | 8.8E-02 | **2.7** | **7.0E-08** | 2.6 | 4.6E-10 |
| RNASE3 | -1.4 | 2.5E-02 | **2.7** | **5.0E-07** | 3.3 | 5.0E-11 |
| SLC1A3 | 1.7 | 1.1E-03 | **2.7** | **8.5E-08** | 2.4 | 7.7E-10 |
| SLC22A4 | 1.8 | 1.4E-08 | **2.7** | **0.0E+00** | 2.5 | 6.6E-27 |
| SLC4A1 | 1.2 | 1.0E+00 | **2.7** | **5.3E-03** | 2.9 | 6.3E-04 |
| SPTA1 | 1.5 | 1.9E-01 | **2.7** | **8.1E-08** | 2.7 | 6.3E-15 |
| TEAD3 | 1.9 | 3.9E-03 | **2.7** | **1.4E-05** | 1.9 | 2.9E-03 |
| TRPV5 | 1.7 | 2.6E-02 | **2.7** | **1.7E-07** | 1.8 | 7.7E-03 |
| AC072022.2 | 1.9 | 3.7E-03 | **2.8** | **3.4E-07** | 2.7 | 4.7E-06 |
| ACSS3 | 1.9 | 1.2E-18 | **2.8** | **9.7E-36** | 2.4 | 1.5E-12 |
| CASP5 | 1.9 | 9.8E-08 | **2.8** | **1.6E-13** | 2.2 | 1.8E-06 |
| DOC2B | 1.2 | 1.3E-01 | **2.8** | **2.8E-06** | 3.0 | 9.4E-08 |
| FOLR3 | 1.8 | 1.2E-02 | **2.8** | **1.7E-09** | 2.5 | 5.8E-06 |
| KLHL2 | 1.9 | 9.1E-14 | **2.8** | **0.0E+00** | 2.3 | 3.1E-24 |
| NLRC4 | 1.7 | 9.7E-15 | **2.8** | **0.0E+00** | 2.5 | 1.0E-28 |
| OSBP2 | 1.0 | 9.2E-01 | **2.8** | **4.1E-04** | 3.1 | 6.4E-04 |
| RGL4 | 1.7 | 1.7E-14 | **2.8** | **6.9E-30** | 2.5 | 4.2E-18 |
| SELENBP1 | 1.1 | 9.9E-01 | **2.8** | **1.9E-03** | 3.0 | 5.7E-04 |
| SIPA1L2 | 1.9 | 5.3E-07 | **2.8** | **1.6E-22** | 2.2 | 3.6E-10 |
| SPTB | 1.2 | 9.4E-01 | **2.8** | **5.8E-03** | 3.1 | 2.9E-04 |
| ST3GAL4 | 1.9 | 3.7E-08 | **2.8** | **2.0E-33** | 2.4 | 4.1E-16 |
| STEAP4 | 1.9 | 4.7E-15 | **2.8** | **6.0E-26** | 2.2 | 1.4E-12 |
| AC011498.4 | 1.6 | 1.8E-05 | **2.9** | **7.4E-26** | 2.3 | 2.7E-14 |
| AL049634.2 | 1.8 | 2.1E-07 | **2.9** | **2.1E-20** | 2.6 | 6.3E-23 |
| CELF3 | 1.1 | 8.3E-01 | **2.9** | **4.4E-05** | 3.4 | 1.5E-08 |
| CLEC4D | 1.7 | 3.1E-13 | **2.9** | **3.9E-32** | 2.5 | 1.1E-18 |
| DHRS13 | 1.9 | 2.4E-09 | **2.9** | **2.8E-34** | 2.3 | 7.6E-13 |
| KCNJ15 | 1.9 | 8.8E-11 | **2.9** | **1.4E-34** | 2.4 | 7.2E-13 |
| LILRA5 | 1.9 | 3.5E-10 | **2.9** | **0.0E+00** | 2.6 | 5.4E-33 |
| LY96 | 1.8 | 8.3E-15 | **2.9** | **1.3E-33** | 2.5 | 7.6E-15 |
| NEBL | 1.2 | 5.4E-02 | **2.9** | **3.4E-05** | 3.1 | 1.1E-05 |
| NSUN7 | 1.9 | 1.0E-06 | **2.9** | **5.3E-17** | 2.4 | 1.4E-08 |
| TSPO2 | 1.2 | 9.6E-01 | **2.9** | **1.6E-05** | 4.0 | 1.0E-06 |
| VNN1 | 1.9 | 2.5E-13 | **2.9** | **4.6E-26** | 2.6 | 1.4E-15 |
| CACNA1E | 1.8 | 1.0E-05 | **3.0** | **0.0E+00** | 2.8 | 4.7E-27 |
| CYYR1 | 1.6 | 3.3E-02 | **3.0** | **1.7E-12** | 3.0 | 3.1E-18 |
| F5 | 1.9 | 4.5E-10 | **3.0** | **0.0E+00** | 2.7 | 1.3E-19 |
| FCGR1B | 1.8 | 1.0E-03 | **3.0** | **7.0E-18** | 2.6 | 6.8E-08 |
| MYO10 | 1.7 | 9.1E-08 | **3.0** | **2.7E-17** | 2.7 | 9.4E-14 |
| PLIN4 | 1.9 | 7.3E-07 | **3.0** | **2.1E-22** | 2.6 | 9.9E-15 |
| TCTEX1D1 | 1.1 | 9.5E-01 | **3.0** | **1.8E-07** | 2.7 | 3.2E-04 |
| THEM5 | 1.1 | 7.9E-01 | **3.0** | **3.8E-03** | 3.6 | 9.1E-04 |
| EPB42 | 1.2 | 8.2E-01 | **3.1** | **1.0E-04** | 3.2 | 1.1E-05 |
| PLB1 | 1.8 | 1.4E-06 | **3.1** | **2.7E-23** | 2.5 | 1.1E-12 |
| CTSG | -1.3 | 2.8E-01 | **3.2** | **6.2E-06** | 4.3 | 3.3E-08 |
| ITLN1 | 1.2 | 8.6E-01 | **3.2** | **2.0E-04** | 4.4 | 4.8E-05 |
| SPATC1 | 1.8 | 1.6E-04 | **3.2** | **1.5E-07** | 3.0 | 1.2E-07 |
| TCN1 | 1.2 | 4.3E-01 | **3.2** | **9.3E-15** | 4.2 | 1.1E-13 |
| PLIN5 | 1.9 | 5.4E-09 | **3.3** | **0.0E+00** | 2.7 | 6.9E-25 |
| CHIT1 | 1.7 | 3.7E-07 | **3.4** | **3.9E-36** | 3.4 | 0.0E+00 |
| CYSTM1 | 1.9 | 1.6E-09 | **3.4** | **0.0E+00** | 3.0 | 4.7E-23 |
| GPR17 | 2.0 | 5.2E-02 | **3.4** | **7.8E-07** | 2.3 | 6.1E-04 |
| CBS | 2.8 | 7.6E-02 | **3.5** | **2.4E-06** | 3.1 | 2.0E-02 |
| GPR84 | 1.9 | 1.0E-02 | **3.5** | **1.2E-10** | 3.8 | 2.5E-10 |
| TARM1 | -1.2 | 9.6E-01 | **3.5** | **1.8E-04** | 4.7 | 2.3E-06 |
| BPI | 1.2 | 2.3E-01 | **3.7** | **2.1E-18** | 4.8 | 6.9E-15 |
| BCAM | 1.3 | 5.7E-01 | **3.8** | **8.1E-06** | 4.8 | 7.6E-07 |
| FSTL4 | 1.9 | 2.2E-02 | **3.8** | **1.1E-08** | 3.0 | 2.8E-07 |
| RETN | 1.2 | 2.7E-01 | **4.1** | **2.2E-11** | 5.4 | 4.3E-17 |
| TWIST2 | 1.7 | 6.5E-02 | **4.2** | **3.6E-04** | 3.7 | 4.5E-08 |
| ELANE | 1.3 | 2.0E-01 | **4.3** | **1.1E-18** | 5.5 | 3.2E-08 |
| ORM2 | 2.0 | 1.0E-01 | **4.3** | **1.6E-10** | 4.8 | 3.6E-08 |
| CA1 | 1.2 | 8.5E-01 | **4.4** | **6.1E-05** | 6.0 | 3.2E-06 |
| STOX2 | 1.2 | 9.1E-01 | **4.4** | **3.6E-04** | 5.9 | 8.6E-09 |
| ERG | 1.2 | 2.7E-01 | **4.5** | **3.0E-18** | 5.3 | 4.0E-15 |
| PAGE2B | 1.2 | 9.4E-01 | **4.5** | **3.8E-09** | 4.8 | 2.7E-18 |
| SERPINB10 | 1.4 | 3.3E-01 | **4.6** | **1.4E-09** | 5.9 | 1.3E-16 |
| ATP2C2 | 1.8 | 9.0E-02 | **5.4** | **1.7E-08** | 6.6 | 7.2E-13 |
| CRISP2 | 2.0 | 4.5E-01 | **5.7** | **9.6E-04** | 5.8 | 6.8E-05 |
| MAOA | 1.6 | 5.9E-01 | **5.7** | **1.2E-06** | 7.0 | 3.0E-08 |
| PRTN3 | 1.6 | 3.9E-01 | **5.7** | **1.5E-12** | 7.1 | 6.7E-10 |
| CEACAM6 | 1.1 | 4.6E-01 | **5.8** | **6.3E-19** | 8.0 | 1.8E-12 |
| INHBA | 1.4 | 7.3E-01 | **5.9** | **4.8E-12** | 8.2 | 1.5E-10 |
| COL17A1 | 1.2 | 5.8E-01 | **6.1** | **9.9E-09** | 7.2 | 5.4E-12 |
| ABCA13 | 1.7 | 5.0E-02 | **6.7** | **4.7E-15** | 9.5 | 2.4E-17 |
| LCN2 | 1.3 | 1.1E-01 | **7.1** | **2.1E-20** | 9.6 | 3.5E-29 |
| DEFA4 | 1.5 | 7.1E-02 | **7.3** | **1.5E-15** | 10.9 | 1.6E-19 |
| CEACAM8 | 1.5 | 6.0E-02 | **7.4** | **1.8E-18** | 10.0 | 1.2E-25 |
| DEFA3 | 1.2 | 5.0E-02 | **7.9** | **3.3E-15** | 10.1 | 4.0E-12 |
| FOXQ1 | 4.2 | 5.8E-02 | **8.4** | **1.7E-04** | 5.8 | 2.8E-03 |
| OLR1 | 1.5 | 9.8E-02 | **8.9** | **5.6E-20** | 13.5 | 0.0E+00 |
| LTF | 1.8 | 4.0E-02 | **9.5** | **1.2E-32** | 14.4 | 1.7E-26 |
| PCOLCE2 | 1.8 | 2.7E-01 | **10.1** | **1.4E-16** | 15.2 | 1.1E-12 |
| OLFM4 | 1.7 | 1.2E-01 | **14.9** | **1.3E-16** | 23.3 | 2.5E-18 |
| KLHDC7A | -1.5 | 7.6E-01 | -3.7 | 1.0E-01 | **-12.2** | **3.1E-05** |
| IGKV2-28 | 1.1 | 4.1E-01 | -2.6 | 7.4E-02 | **-3.8** | **2.4E-04** |
| IGKV2D-28 | -1.1 | 3.1E-01 | -2.5 | 1.8E-01 | **-3.6** | **1.6E-04** |
| ARPP21 | -1.0 | 7.6E-01 | -1.6 | 1.8E-01 | **-2.9** | **2.9E-03** |
| CAV1 | -1.7 | 6.9E-01 | -1.8 | 8.8E-02 | **-2.9** | **7.6E-05** |
| TPSAB1 | -1.9 | 3.1E-01 | 1.0 | 5.5E-01 | **-2.8** | **3.1E-03** |
| IGLV10-54 | 1.1 | 7.5E-01 | -1.8 | 5.5E-01 | **-2.7** | **9.4E-04** |
| SLC2A10 | -1.1 | 7.6E-01 | -2.3 | 8.2E-02 | **-2.6** | **4.8E-03** |
| EMP1 | -1.6 | 6.8E-03 | -1.7 | 1.7E-03 | **-2.5** | **2.6E-07** |
| IGHV3-72 | -1.5 | 7.4E-01 | -2.2 | 5.4E-01 | **-2.5** | **1.9E-03** |
| CCR9 | -1.3 | 8.4E-01 | -1.8 | 2.8E-05 | **-2.4** | **1.2E-12** |
| ENTPD2 | -1.4 | 1.1E-01 | -1.9 | 7.8E-03 | **-2.4** | **1.5E-08** |
| GCSAML | -1.1 | 8.0E-01 | -1.7 | 5.7E-03 | **-2.4** | **4.8E-06** |
| SNAI1 | -1.4 | 3.9E-02 | -1.8 | 1.7E-02 | **-2.4** | **3.6E-06** |
| CAV2 | -1.2 | 5.5E-01 | -1.9 | 3.6E-04 | **-2.3** | **8.5E-07** |
| CNN1 | -1.0 | 7.9E-01 | -1.5 | 3.1E-01 | **-2.3** | **5.1E-04** |
| FKBP2 | -1.5 | 6.9E-02 | -1.8 | 9.4E-04 | **-2.3** | **2.3E-02** |
| **GATA6** | -1.5 | 7.9E-02 | -1.7 | 5.9E-02 | **-2.3** | **1.3E-04** |
| RBM20 | -1.6 | 2.6E-03 | -1.7 | 1.7E-05 | **-2.3** | **1.9E-08** |
| BICC1 | 1.0 | 5.5E-01 | -1.2 | 4.4E-01 | **-2.2** | **4.2E-03** |
| CACNB4 | -1.6 | 2.7E-08 | -1.8 | 5.0E-11 | **-2.2** | **1.9E-21** |
| EREG | -1.2 | 7.7E-01 | -1.9 | 4.3E-02 | **-2.2** | **2.9E-03** |
| **FGFR2** | -1.8 | 1.1E-16 | -1.9 | 1.9E-11 | **-2.2** | **1.6E-05** |
| FTCD | -1.7 | 4.9E-02 | -1.9 | 2.5E-01 | **-2.2** | **1.6E-02** |
| LCN10 | -1.4 | 1.1E-01 | -1.2 | 2.1E-01 | **-2.2** | **2.0E-04** |
| RGS17 | -1.5 | 3.3E-06 | -1.3 | 1.4E-01 | **-2.2** | **1.4E-07** |
| ROR1 | -1.7 | 1.1E-01 | -1.8 | 8.0E-02 | **-2.2** | **4.7E-02** |
| TM4SF19 | -1.3 | 3.9E-01 | -1.9 | 3.3E-02 | **-2.2** | **1.5E-05** |
| TP63 | -1.4 | 1.3E-01 | -1.7 | 1.2E-02 | **-2.2** | **3.0E-05** |
| TRAJ58 | -1.2 | 6.0E-01 | -1.3 | 2.8E-01 | **-2.2** | **2.1E-03** |
| ZDHHC11B | -1.4 | 8.2E-02 | -1.9 | 2.0E-07 | **-2.2** | **7.5E-10** |
| CDHR1 | -1.3 | 4.8E-03 | -1.8 | 3.1E-09 | **-2.1** | **4.5E-11** |
| GFPT2 | -1.2 | 1.9E-01 | -1.9 | 7.6E-04 | **-2.1** | **8.9E-07** |
| GOLGA8T | -1.5 | 7.0E-03 | -1.7 | 1.7E-03 | **-2.1** | **3.9E-06** |
| IGHV3-53 | -1.1 | 7.5E-01 | -1.8 | 4.8E-02 | **-2.1** | **3.4E-03** |
| KIR2DL3 | -1.2 | 3.6E-01 | -1.8 | 7.5E-03 | **-2.1** | **3.5E-06** |
| KIR3DL1 | -1.2 | 2.1E-01 | -1.8 | 5.8E-04 | **-2.1** | **6.7E-07** |
| MT-ATP8 | -1.4 | 2.2E-02 | -1.9 | 3.1E-04 | **-2.1** | **5.3E-06** |
| NAV3 | -1.2 | 9.2E-01 | -1.5 | 1.8E-01 | **-2.1** | **1.8E-03** |
| NKX3-1 | -1.2 | 9.9E-01 | -1.7 | 1.5E-01 | **-2.1** | **7.7E-03** |
| **SOX5** | -1.1 | 5.3E-01 | -1.7 | 7.8E-03 | **-2.1** | **3.8E-06** |
| TRDJ4 | -1.3 | 1.3E-01 | -1.8 | 1.3E-04 | **-2.1** | **9.6E-08** |
| VMO1 | -1.1 | 8.1E-01 | -1.8 | 1.3E-02 | **-2.1** | **1.1E-02** |
| B3GAT1 | -1.4 | 5.1E-02 | -1.9 | 2.1E-04 | **-2.0** | **1.9E-08** |
| CDR2L | 1.0 | 7.4E-01 | -1.6 | 8.5E-03 | **-2.0** | **1.1E-05** |
| COL5A3 | -1.4 | 9.2E-02 | -1.9 | 2.3E-05 | **-2.0** | **7.7E-07** |
| IGLV2-14 | -1.3 | 4.0E-01 | -1.9 | 5.2E-12 | **-2.0** | **3.6E-14** |
| **IL5RA** | -1.8 | 9.1E-09 | -1.9 | 6.9E-03 | **-2.0** | **5.8E-03** |
| LRFN2 | -1.5 | 8.1E-02 | -1.4 | 3.7E-02 | **-2.0** | **1.5E-03** |
| RTKN | -1.4 | 7.1E-02 | -1.8 | 1.7E-04 | **-2.0** | **7.5E-16** |
| SCN4A | -1.6 | 2.0E-01 | -1.5 | 1.4E-01 | **-2.0** | **6.0E-04** |
| SLC12A3 | -1.1 | 4.6E-01 | -1.9 | 3.8E-04 | **-2.0** | **3.1E-05** |
| TNNC1 | -1.8 | 2.6E-01 | -1.3 | 4.1E-01 | **-2.0** | **5.4E-03** |
| TRDD3 | -1.8 | 1.1E-01 | -1.8 | 4.2E-02 | **-2.0** | **6.4E-03** |
| MARCH8 | -1.0 | 7.2E-01 | 1.9 | 1.6E-03 | **2.0** | **1.8E-04** |
| ARL4A | 1.0 | 6.2E-01 | 1.8 | 1.1E-03 | **2.0** | **1.1E-07** |
| ATP5F1E | 1.3 | 9.6E-03 | 1.8 | 9.6E-15 | **2.0** | **4.0E-10** |
| **C1QA** | 1.1 | 2.2E-01 | 1.3 | 5.6E-02 | **2.0** | **9.6E-05** |
| **C1QB** | 1.2 | 3.0E-01 | 1.4 | 5.9E-02 | **2.0** | **6.2E-05** |
| DCAF12 | -1.1 | 7.0E-01 | 1.9 | 1.4E-02 | **2.0** | **3.4E-03** |
| DNAJC6 | -1.1 | 4.9E-01 | 1.8 | 2.8E-03 | **2.0** | **1.2E-05** |
| FAM104A | 1.0 | 9.9E-01 | 1.8 | 1.2E-03 | **2.0** | **1.8E-04** |
| FGF2 | 1.2 | 6.4E-01 | 1.2 | 7.6E-01 | **2.0** | **1.0E-02** |
| GPRC5B | -1.1 | 7.7E-01 | 1.4 | 2.8E-03 | **2.0** | **4.1E-08** |
| PRDX2 | 1.0 | 9.4E-01 | 1.9 | 3.0E-05 | **2.0** | **8.1E-06** |
| RBM38 | 1.1 | 9.6E-01 | 1.9 | 9.0E-03 | **2.0** | **3.5E-03** |
| RIPOR3 | -1.1 | 5.0E-01 | 1.7 | 1.5E-02 | **2.0** | **1.6E-04** |
| RNF10 | 1.1 | 9.8E-01 | 1.9 | 6.0E-04 | **2.0** | **2.1E-05** |
| RPL23 | 1.2 | 4.3E-01 | 1.6 | 3.1E-04 | **2.0** | **5.0E-05** |
| RPS27 | 1.2 | 5.4E-01 | 1.6 | 3.6E-05 | **2.0** | **3.2E-06** |
| SIAH2 | -1.1 | 6.3E-01 | 1.6 | 1.7E-01 | **2.0** | **3.0E-02** |
| SMIM5 | 1.1 | 9.6E-01 | 1.9 | 3.2E-04 | **2.0** | **7.8E-06** |
| STRADB | 1.0 | 9.5E-01 | 1.9 | 6.9E-04 | **2.0** | **2.3E-05** |
| SYT2 | 1.3 | 5.6E-01 | 1.5 | 1.7E-01 | **2.0** | **3.1E-02** |
| TRAPPC3L | 1.6 | 7.4E-03 | 1.7 | 3.3E-03 | **2.0** | **9.7E-03** |
| AC000093.1 | -1.2 | 6.3E-01 | 1.7 | 1.3E-01 | **2.1** | **2.7E-02** |
| ANKRD35 | 1.2 | 6.8E-01 | 1.8 | 2.3E-01 | **2.1** | **2.6E-03** |
| BCL2L1 | -1.0 | 7.5E-01 | 1.9 | 6.6E-03 | **2.1** | **2.2E-03** |
| C9orf78 | -1.0 | 6.3E-01 | 1.8 | 1.4E-01 | **2.1** | **3.6E-02** |
| ELOVL3 | -1.0 | 9.6E-01 | 1.7 | 5.8E-02 | **2.1** | **7.0E-03** |
| GADD45G | 1.5 | 2.0E-01 | 1.8 | 4.4E-04 | **2.1** | **1.1E-05** |
| HIST3H2BB | 1.0 | 7.7E-01 | 1.7 | 1.1E-01 | **2.1** | **1.7E-02** |
| ITGA7 | 1.3 | 1.2E-01 | 1.8 | 1.4E-03 | **2.1** | **8.8E-07** |
| M1AP | -1.0 | 9.9E-01 | 1.8 | 1.1E-03 | **2.1** | **1.5E-04** |
| MFSD2B | 1.0 | 9.6E-01 | 1.9 | 5.1E-04 | **2.1** | **3.9E-07** |
| MS4A3 | -1.2 | 5.7E-01 | 1.7 | 4.3E-04 | **2.1** | **1.6E-04** |
| PTGES | 1.4 | 1.4E-01 | 1.8 | 2.5E-05 | **2.1** | **2.0E-06** |
| PTX3 | 1.1 | 6.8E-01 | 1.6 | 2.7E-02 | **2.1** | **1.4E-05** |
| RPL21 | 1.3 | 2.0E-01 | 1.7 | 1.3E-07 | **2.1** | **3.9E-08** |
| RPL41 | 1.2 | 4.5E-01 | 1.8 | 2.5E-06 | **2.1** | **1.0E-06** |
| RPS3A | 1.3 | 3.2E-01 | 1.7 | 8.0E-06 | **2.1** | **8.7E-06** |
| RUNDC3A | -1.1 | 4.7E-01 | 1.9 | 1.6E-02 | **2.1** | **2.2E-04** |
| SIGLEC11 | 1.7 | 3.0E-04 | 1.7 | 3.9E-04 | **2.1** | **3.6E-05** |
| XKR7 | 1.6 | 3.2E-02 | 1.8 | 5.8E-05 | **2.1** | **2.5E-06** |
| **C1QC** | -1.1 | 7.6E-01 | 1.2 | 4.0E-01 | **2.2** | **2.4E-03** |
| DNAH14 | 1.3 | 5.6E-01 | 1.4 | 4.5E-01 | **2.2** | **9.9E-03** |
| DNAH17 | 1.5 | 4.8E-01 | 1.8 | 1.9E-02 | **2.2** | **2.7E-03** |
| FAM210B | -1.0 | 7.7E-01 | 1.9 | 2.8E-02 | **2.2** | **1.5E-03** |
| IQGAP3 | -1.3 | 1.8E-01 | 1.8 | 8.6E-02 | **2.2** | **1.2E-02** |
| ITGA9 | 1.3 | 1.9E-02 | 1.7 | 8.6E-05 | **2.2** | **2.2E-08** |
| OSBPL6 | 1.5 | 2.2E-02 | 1.7 | 1.2E-04 | **2.2** | **4.1E-05** |
| PAQR9 | 1.6 | 3.4E-01 | 2.3 | 5.7E-02 | **2.2** | **1.2E-02** |
| PKD1L3 | 1.1 | 8.8E-01 | 1.4 | 2.4E-01 | **2.2** | **6.8E-03** |
| RPL34 | 1.2 | 4.4E-01 | 1.6 | 1.5E-04 | **2.2** | **6.6E-05** |
| SLFN14 | 1.1 | 9.7E-01 | 1.9 | 4.8E-05 | **2.2** | **2.6E-08** |
| **CD24** | -1.1 | 6.1E-01 | 1.9 | 4.0E-03 | **2.3** | **2.3E-05** |
| FAM20A | 1.3 | 3.5E-01 | 1.5 | 1.4E-01 | **2.3** | **1.8E-05** |
| LPL | 1.2 | 4.8E-01 | 1.3 | 3.0E-01 | **2.3** | **1.0E-02** |
| PHACTR3 | -1.1 | 7.7E-01 | 1.4 | 3.2E-01 | **2.3** | **6.8E-03** |
| PLOD2 | -1.0 | 8.5E-01 | 1.7 | 2.0E-05 | **2.3** | **2.2E-04** |
| PTGFR | -1.1 | 7.7E-01 | 1.7 | 4.0E-04 | **2.3** | **8.9E-07** |
| RPL39 | 1.2 | 4.9E-01 | 1.6 | 1.2E-02 | **2.3** | **2.7E-04** |
| RPL7 | 1.5 | 5.5E-02 | 1.8 | 8.1E-06 | **2.3** | **2.3E-07** |
| SLC2A5 | 1.1 | 6.5E-01 | 1.8 | 1.5E-03 | **2.3** | **5.9E-05** |
| UNC5A | 1.0 | 9.0E-01 | 1.1 | 5.4E-01 | **2.3** | **4.4E-02** |
| SCN5A | -1.0 | 9.6E-01 | 1.2 | 8.3E-01 | **2.4** | **2.4E-03** |
| SLC2A4 | 1.1 | 9.5E-01 | 2.2 | 9.4E-02 | **2.5** | **1.4E-02** |
| SLC28A3 | 1.2 | 4.9E-01 | 1.8 | 1.5E-02 | **2.6** | **7.2E-08** |
| ACHE | -1.0 | 9.3E-01 | 2.3 | 9.8E-02 | **3.4** | **1.7E-02** |
| RELN | -1.5 | 6.2E-01 | 2.9 | 9.2E-02 | **4.0** | **5.9E-03** |
