# Supplementary material for: Pregnancy-associated systemic gene expression compared to a pre-pregnancy baseline, among healthy women with term pregnancies
Source: Front Immunol. 2023 Jun 5;14:1161084. doi: 10.3389/fimmu.2023.1161084 (PMC10277629; doi:10.3389/fimmu.2023.1161084)
Supplement: Supplementary file 4 [file Table_2.docx]

**Table S2.**

**Genes with significant pregnancy-associated expression in the GEE model adjusted for cell type proportions.**

Fold-changes (FCs) in expression at each trimester (compared to the pre-pregnancy [T0] baseline) are shown (to 1 decimal place), together with FDR-adjusted p values (FDR), for genes that were significantly associated with pregnancy (FC≤-2 or FC≥2, FDR<0.05). FCs corresponding to a decrease in expression are shown as negative values. FC and FDR values in bold indicate at which trimester a gene first became significantly associated with pregnancy.

|  | **T1** | | **T2** | | **T3** | |
| --- | --- | --- | --- | --- | --- | --- |
| **Gene name** | **FC (vs T0)** | **FDR** | **FC (vs T0)** | **FDR** | **FC (vs T0)** | **FDR** |
| RSAD2 | **-6.5** | **1.4E-02** | -13.4 | 8.4E-07 | -7.4 | 2.3E-03 |
| CMPK2 | **-4.4** | **1.5E-02** | -7.2 | 1.6E-04 | -4.1 | 1.4E-02 |
| OAS3 | **-4.1** | **2.3E-02** | -7.7 | 6.7E-04 | -4.9 | 1.5E-02 |
| IDO1 | **-3.7** | **8.7E-10** | -3.3 | 4.9E-04 | -2.6 | 6.5E-03 |
| EPSTI1 | **-3.2** | **1.7E-02** | -4.9 | 1.0E-03 | -3.4 | 2.2E-02 |
| AGRN | **-3.0** | **3.9E-02** | -5.3 | 1.2E-03 | -3.5 | 2.1E-02 |
| OR13A1 | **-2.8** | **4.4E-02** | -2.3 | 2.1E-01 | -1.9 | 3.1E-01 |
| FBXO39 | **-2.7** | **1.8E-02** | -4.7 | 4.9E-04 | -3.8 | 3.7E-04 |
| LY6E | **-2.5** | **1.4E-02** | -3.8 | 4.6E-02 | -2.9 | 1.0E-01 |
| UBQLN3 | **-2.5** | **5.9E-03** | -3.0 | 7.9E-01 | -1.8 | 9.3E-01 |
| CCNA1 | **-2.4** | **2.2E-02** | -4.9 | 1.2E-04 | -2.4 | 2.0E-02 |
| IL34 | **-2.4** | **2.5E-02** | -2.0 | 5.9E-01 | -2.0 | 4.5E-01 |
| OAS2 | **-2.4** | **4.8E-02** | -3.4 | 8.9E-03 | -2.7 | 3.2E-02 |
| UPK2 | **-2.4** | **1.3E-02** | -4.6 | 5.6E-02 | -2.7 | 9.5E-02 |
| CACNG6 | **-2.2** | **9.9E-05** | -3.1 | 2.7E-03 | -4.0 | 2.1E-03 |
| MDK | **-2.2** | **4.2E-02** | -2.6 | 4.2E-02 | -1.9 | 1.1E-01 |
| ABTB2 | **-2.0** | **3.7E-09** | -2.3 | 2.0E-05 | -1.8 | 1.8E-03 |
| ADAMTS14 | **-2.0** | **4.0E-02** | -1.9 | 9.0E-02 | -1.2 | 7.4E-01 |
| IRF6 | **-2.0** | **2.1E-02** | -3.0 | 3.0E-02 | -1.8 | 1.8E-01 |
| SCUBE3 | **-2.0** | **5.0E-02** | -2.2 | 6.5E-01 | -2.1 | 1.9E-01 |
| FAM167B | **2.0** | **4.1E-02** | 2.0 | 5.1E-01 | 1.3 | 9.5E-01 |
| CAPN13 | **2.2** | **8.6E-03** | 3.1 | 8.5E-04 | 3.3 | 3.3E-05 |
| PSMA8 | **2.2** | **6.8E-03** | 3.0 | 6.3E-04 | 3.3 | 5.4E-10 |
| ZNF492 | **2.2** | **4.9E-02** | 3.2 | 2.3E-02 | 2.0 | 1.5E-01 |
| CD177 | **2.9** | **2.1E-04** | 4.6 | 4.9E-02 | 6.5 | 6.3E-03 |
| CBSL | **3.1** | **1.1E-02** | 3.9 | 4.4E-08 | 3.6 | 9.2E-05 |
| SERPING1 | -4.0 | 1.2E-01 | **-8.6** | **7.7E-04** | -5.4 | 2.6E-02 |
| OTOF | -3.9 | 1.2E-01 | **-8.4** | **1.6E-02** | -3.9 | 1.3E-01 |
| LAMP3 | -4.1 | 1.1E-01 | **-8.3** | **4.2E-03** | -5.8 | 1.6E-02 |
| IFI44L | -4.7 | 6.0E-02 | **-7.9** | **4.5E-03** | -4.6 | 5.7E-02 |
| ISG15 | -4.2 | 7.8E-02 | **-7.6** | **4.5E-03** | -5.0 | 2.7E-02 |
| IFIT1 | -3.8 | 1.2E-01 | **-7.4** | **1.5E-03** | -3.9 | 7.2E-02 |
| HERC5 | -3.6 | 6.5E-02 | **-6.5** | **2.0E-04** | -4.4 | 1.1E-02 |
| MX1 | -3.2 | 9.7E-02 | **-5.6** | **4.8E-04** | -3.6 | 1.7E-02 |
| IFI44 | -3.4 | 7.8E-02 | **-5.1** | **3.5E-03** | -3.5 | 3.5E-02 |
| IFI6 | -3.1 | 1.3E-01 | **-5.1** | **4.7E-03** | -3.3 | 6.9E-02 |
| C10orf82 | 1.6 | 7.2E-01 | **-5.1** | **1.5E-05** | -26.0 | 4.5E-08 |
| SIGLEC1 | -3.4 | 9.7E-02 | **-5.0** | **2.8E-02** | -2.7 | 2.5E-01 |
| GATA2 | -1.9 | 4.5E-05 | **-4.9** | **8.2E-07** | -8.9 | 1.7E-26 |
| ITGB8 | -1.8 | 2.8E-03 | **-4.9** | **2.6E-06** | -12.0 | 9.1E-19 |
| USP18 | -3.4 | 7.1E-02 | **-4.8** | **2.3E-03** | -3.8 | 1.4E-02 |
| IFIT3 | -2.7 | 1.5E-01 | **-4.7** | **7.0E-03** | -3.3 | 6.5E-02 |
| HDC | -1.8 | 6.3E-02 | **-4.6** | **9.5E-04** | -10.9 | 6.6E-18 |
| SEPTIN4 | -2.5 | 3.0E-01 | **-4.5** | **2.7E-02** | -3.3 | 8.4E-02 |
| DCT | -1.5 | 3.2E-01 | **-4.5** | **2.8E-03** | -2.6 | 1.7E-02 |
| OASL | -2.8 | 1.6E-01 | **-4.4** | **1.8E-02** | -3.6 | 3.4E-02 |
| AKAP12 | -1.4 | 6.1E-02 | **-4.2** | **8.5E-05** | -13.2 | 2.8E-28 |
| MS4A2 | -1.7 | 3.4E-01 | **-4.1** | **4.4E-02** | -9.1 | 2.6E-07 |
| TMEM132C | -1.2 | 6.5E-01 | **-3.9** | **2.8E-02** | -1.7 | 4.1E-01 |
| OAS1 | -2.7 | 6.3E-02 | **-3.8** | **1.4E-02** | -3.0 | 5.1E-02 |
| SCUBE1 | -1.4 | 6.0E-01 | **-3.8** | **1.7E-04** | -2.9 | 5.5E-04 |
| IL4 | -1.6 | 1.2E-01 | **-3.7** | **2.5E-03** | -11.2 | 2.6E-09 |
| IGHV5-51 | -2.0 | 5.0E-01 | **-3.6** | **1.9E-02** | -3.1 | 2.0E-02 |
| RSPH9 | -2.2 | 1.5E-01 | **-3.2** | **2.4E-02** | -2.8 | 3.4E-02 |
| XAF1 | -2.2 | 1.4E-01 | **-3.2** | **9.8E-03** | -2.5 | 5.8E-02 |
| CCDC194 | -2.1 | 8.9E-02 | **-3.2** | **4.7E-03** | -2.5 | 1.7E-02 |
| RAPGEF3 | -1.8 | 4.6E-01 | **-3.2** | **3.7E-02** | -1.8 | 3.8E-01 |
| DHX58 | -2.3 | 7.4E-02 | **-3.1** | **4.5E-03** | -2.5 | 2.1E-02 |
| DDX60 | -2.1 | 2.0E-01 | **-3.0** | **1.1E-02** | -2.4 | 6.1E-02 |
| RTP4 | -2.1 | 1.3E-01 | **-2.9** | **4.3E-02** | -2.3 | 1.2E-01 |
| AC005520.1 | -1.7 | 4.3E-02 | **-2.8** | **8.5E-03** | -2.0 | 2.3E-03 |
| LRRN2 | -1.7 | 1.1E-01 | **-2.8** | **1.0E-03** | -1.6 | 3.8E-01 |
| ZCCHC2 | -1.9 | 5.0E-02 | **-2.7** | **5.8E-04** | -2.2 | 4.6E-03 |
| RYR3 | -1.5 | 2.7E-01 | **-2.7** | **8.8E-03** | -4.7 | 1.0E-06 |
| KRT5 | -1.1 | 9.2E-01 | **-2.7** | **2.0E-04** | -1.8 | 4.1E-02 |
| PARP12 | -2.0 | 7.6E-02 | **-2.6** | **6.6E-03** | -2.2 | 2.3E-02 |
| HELZ2 | -1.9 | 2.2E-02 | **-2.6** | **1.1E-03** | -2.0 | 1.0E-02 |
| HERC6 | -1.9 | 1.1E-01 | **-2.6** | **1.3E-03** | -2.2 | 1.6E-02 |
| IFIH1 | -1.9 | 1.9E-01 | **-2.6** | **2.5E-02** | -2.2 | 7.9E-02 |
| WNT6 | -1.5 | 3.0E-01 | **-2.6** | **1.5E-02** | -1.7 | 1.5E-01 |
| IGHV3-64D | -1.4 | 4.2E-02 | **-2.5** | **2.6E-03** | -1.9 | 8.0E-06 |
| WNK2 | -1.4 | 6.3E-01 | **-2.5** | **4.4E-02** | -2.0 | 1.2E-02 |
| TTC21A | -1.9 | 5.0E-02 | **-2.4** | **3.6E-03** | -2.3 | 7.9E-03 |
| PML | -1.8 | 1.2E-01 | **-2.4** | **5.6E-03** | -2.0 | 3.6E-02 |
| SLC45A3 | -1.4 | 4.3E-04 | **-2.4** | **4.9E-08** | -3.3 | 3.5E-17 |
| ENPP3 | -1.3 | 1.9E-01 | **-2.4** | **6.6E-03** | -4.7 | 1.2E-07 |
| RFX8 | -1.1 | 8.7E-01 | **-2.4** | **4.1E-02** | -1.8 | 8.9E-02 |
| MT2A | -1.9 | 1.2E-01 | **-2.3** | **2.7E-02** | -2.1 | 5.9E-02 |
| STAT2 | -1.9 | 1.4E-01 | **-2.3** | **2.9E-02** | -1.9 | 8.6E-02 |
| EIF2AK2 | -1.9 | 1.4E-01 | **-2.2** | **2.9E-02** | -1.9 | 7.8E-02 |
| PNPT1 | -1.8 | 1.4E-01 | **-2.2** | **2.2E-02** | -2.0 | 6.6E-02 |
| KIAA1958 | -1.7 | 1.1E-01 | **-2.2** | **4.0E-02** | -1.9 | 5.0E-02 |
| KPTN | -1.7 | 2.1E-01 | **-2.2** | **3.6E-02** | -1.8 | 1.3E-01 |
| CPA3 | -1.1 | 2.7E-01 | **-2.2** | **1.7E-03** | -5.0 | 9.9E-13 |
| SLC2A10 | -1.1 | 4.6E-01 | **-2.2** | **2.4E-02** | -2.5 | 6.4E-03 |
| MOV10 | -1.7 | 1.5E-01 | **-2.1** | **3.3E-02** | -1.9 | 5.7E-02 |
| PARP10 | -1.7 | 1.3E-01 | **-2.1** | **2.6E-02** | -1.8 | 5.3E-02 |
| RNF213 | -1.7 | 7.5E-02 | **-2.1** | **7.3E-03** | -1.8 | 1.5E-02 |
| KIAA0895L | -1.6 | 1.8E-01 | **-2.1** | **6.2E-03** | -1.7 | 9.8E-02 |
| MAP1LC3A | -1.6 | 5.8E-05 | **-2.1** | **3.8E-05** | -1.8 | 3.5E-04 |
| RASAL2 | -1.4 | 2.8E-03 | **-2.1** | **3.4E-05** | -1.5 | 1.6E-02 |
| PTGER3 | -1.1 | 4.4E-01 | **-2.1** | **6.5E-06** | -3.7 | 2.9E-16 |
| OR52K2 | -1.6 | 7.0E-02 | **-2.0** | **3.2E-02** | -1.8 | 1.4E-02 |
| SHFL | -1.6 | 8.9E-02 | **-2.0** | **7.1E-03** | -1.8 | 2.7E-02 |
| TOR1B | -1.6 | 2.0E-01 | **-2.0** | **3.6E-02** | -1.8 | 6.6E-02 |
| HSH2D | -1.5 | 6.8E-02 | **-2.0** | **1.6E-03** | -1.8 | 2.3E-03 |
| RPL39L | -1.3 | 4.3E-01 | **-2.0** | **3.2E-02** | -1.8 | 6.7E-02 |
| PHYHD1 | -1.2 | 5.1E-01 | **-2.0** | **9.9E-03** | -1.6 | 7.4E-02 |
| FCER1A | -1.1 | 5.7E-01 | **-2.0** | **1.7E-02** | -3.5 | 1.6E-06 |
| COL17A1 | -1.7 | 9.6E-01 | **2.0** | **2.0E-02** | 2.9 | 3.4E-05 |
| ZDHHC19 | 1.2 | 4.9E-01 | **2.0** | **2.7E-02** | 2.7 | 3.3E-04 |
| SEMA6B | 1.3 | 1.7E-01 | **2.0** | **1.6E-02** | 1.9 | 8.5E-03 |
| TPT1 | 1.3 | 3.4E-01 | **2.0** | **7.2E-03** | 2.0 | 2.0E-03 |
| SHROOM4 | 1.4 | 1.1E-01 | **2.0** | **9.3E-03** | 2.1 | 2.9E-03 |
| OMG | 1.5 | 1.4E-03 | **2.0** | **3.3E-07** | 1.7 | 8.7E-04 |
| INHBB | 1.6 | 5.5E-02 | **2.0** | **1.1E-03** | 2.0 | 1.4E-04 |
| TRMT9B | 1.7 | 1.5E-01 | **2.0** | **3.3E-04** | 1.4 | 2.4E-02 |
| GALNT14 | 1.8 | 6.0E-03 | **2.0** | **6.4E-03** | 2.0 | 2.3E-03 |
| ATP2C2 | 1.1 | 7.5E-01 | **2.1** | **2.0E-02** | 3.3 | 1.1E-04 |
| SIAH3 | 1.3 | 4.5E-01 | **2.1** | **3.0E-02** | 1.6 | 1.1E-01 |
| EVI2A | 1.5 | 1.8E-01 | **2.1** | **2.8E-03** | 1.8 | 1.8E-02 |
| RPL23 | 1.5 | 6.0E-02 | **2.1** | **2.4E-03** | 2.3 | 1.4E-04 |
| SMIM30 | 1.5 | 3.1E-02 | **2.1** | **1.9E-03** | 2.2 | 3.1E-05 |
| S100A9 | 1.6 | 2.2E-02 | **2.1** | **4.1E-04** | 2.4 | 3.5E-06 |
| AC090517.4 | 1.9 | 3.6E-02 | **2.1** | **3.9E-03** | 1.7 | 3.7E-03 |
| COX7C | 1.4 | 3.9E-02 | **2.2** | **4.2E-05** | 2.1 | 2.5E-07 |
| RPL31 | 1.5 | 1.4E-02 | **2.2** | **1.8E-04** | 2.3 | 9.2E-05 |
| RPL9 | 1.5 | 4.1E-02 | **2.2** | **4.8E-04** | 2.2 | 2.4E-05 |
| RPS27 | 1.5 | 1.1E-01 | **2.2** | **1.7E-03** | 2.4 | 8.9E-05 |
| S100P | 1.6 | 1.4E-01 | **2.2** | **3.8E-02** | 2.2 | 7.2E-04 |
| STPG2 | 1.6 | 1.3E-01 | **2.2** | **3.6E-03** | 2.1 | 1.1E-02 |
| ITGB4 | 1.8 | 6.2E-03 | **2.2** | **5.4E-05** | 2.5 | 2.1E-05 |
| ELANE | -1.0 | 8.0E-01 | **2.3** | **1.3E-03** | 3.4 | 8.0E-06 |
| BCL2A1 | 1.6 | 4.2E-03 | **2.3** | **6.9E-09** | 2.4 | 6.7E-12 |
| POLR2K | 1.6 | 3.0E-02 | **2.3** | **1.4E-03** | 2.3 | 2.8E-05 |
| RPS7 | 1.6 | 2.2E-02 | **2.3** | **7.9E-04** | 2.4 | 1.3E-04 |
| SRGAP1 | 1.6 | 5.2E-04 | **2.3** | **8.5E-04** | 2.2 | 6.3E-05 |
| RNASE3 | -1.5 | 5.2E-01 | **2.4** | **7.6E-03** | 3.0 | 1.3E-05 |
| BPI | -1.1 | 8.4E-01 | **2.4** | **1.0E-03** | 3.3 | 1.6E-06 |
| RPL34 | 1.5 | 1.5E-03 | **2.4** | **2.1E-05** | 2.7 | 7.8E-07 |
| GRIK3 | 1.7 | 1.4E-02 | **2.4** | **1.7E-03** | 1.2 | 2.1E-01 |
| ARG1 | 1.5 | 1.0E-01 | **2.5** | **3.7E-03** | 3.3 | 4.4E-05 |
| RPL41 | 1.5 | 3.0E-01 | **2.5** | **3.6E-02** | 2.5 | 7.4E-03 |
| SH2D4A | 1.8 | 8.5E-02 | **2.5** | **2.6E-02** | 1.7 | 1.2E-01 |
| IQGAP3 | -1.0 | 9.5E-01 | **2.6** | **1.7E-02** | 2.7 | 4.2E-04 |
| RPS3A | 1.7 | 2.5E-02 | **2.6** | **1.4E-03** | 2.8 | 1.2E-04 |
| CKS2 | 1.9 | 4.9E-02 | **2.7** | **3.3E-03** | 2.6 | 1.9E-04 |
| RPL39 | 1.7 | 1.7E-02 | **2.8** | **4.1E-03** | 3.4 | 5.2E-05 |
| HHLA2 | 1.4 | 8.3E-01 | **2.9** | **3.2E-02** | 1.9 | 2.4E-01 |
| S100A8 | 1.9 | 1.8E-03 | **3.1** | **1.8E-12** | 3.6 | 2.7E-19 |
| MCEMP1 | 1.7 | 1.8E-01 | **3.2** | **7.3E-05** | 3.4 | 4.7E-06 |
| S100A12 | 1.9 | 5.8E-03 | **3.4** | **3.4E-09** | 4.0 | 1.5E-10 |
| CEACAM6 | -1.2 | 5.9E-01 | **3.5** | **3.8E-04** | 5.4 | 2.9E-07 |
| RETN | 1.2 | 5.4E-01 | **3.6** | **1.2E-06** | 4.6 | 8.8E-13 |
| DEFA3 | -1.2 | 1.6E-01 | **3.7** | **1.4E-05** | 5.6 | 1.2E-07 |
| DEFA4 | 1.1 | 4.4E-01 | **3.9** | **7.7E-05** | 6.7 | 2.9E-12 |
| SERPINB10 | 1.2 | 1.7E-01 | **3.9** | **1.2E-05** | 4.8 | 3.8E-10 |
| LCN2 | -1.1 | 6.3E-01 | **4.1** | **3.2E-05** | 6.3 | 2.4E-13 |
| ERG | 1.2 | 4.1E-01 | **4.2** | **2.6E-06** | 4.9 | 2.7E-11 |
| STOX2 | 1.2 | 9.4E-01 | **4.2** | **3.2E-02** | 5.7 | 1.1E-04 |
| ABCA13 | 1.3 | 4.9E-01 | **4.2** | **2.8E-04** | 6.6 | 3.9E-09 |
| DEFA1 | -1.1 | 4.6E-01 | **4.4** | **8.1E-05** | 6.4 | 3.0E-05 |
| CEACAM8 | 1.1 | 3.8E-01 | **4.4** | **4.2E-05** | 6.7 | 7.1E-12 |
| INHBA | 1.2 | 9.3E-01 | **4.4** | **2.6E-02** | 6.4 | 6.4E-04 |
| PRTN3 | 1.5 | 8.2E-01 | **5.0** | **7.5E-03** | 6.3 | 1.0E-06 |
| CAMP | 1.8 | 3.1E-02 | **5.3** | **1.2E-07** | 7.0 | 2.4E-17 |
| LTF | 1.3 | 2.2E-01 | **5.7** | **2.5E-05** | 9.8 | 1.1E-12 |
| HTRA3 | 1.6 | 1.3E-01 | **6.3** | **2.5E-13** | 9.7 | 6.2E-14 |
| OLR1 | 1.3 | 3.1E-01 | **6.6** | **5.4E-10** | 10.4 | 7.8E-25 |
| CRISP3 | 1.8 | 3.0E-02 | **7.3** | **7.2E-11** | 10.7 | 1.4E-29 |
| MMP8 | 1.8 | 1.1E-02 | **8.3** | **2.9E-10** | 13.5 | 2.7E-26 |
| OLFM4 | 1.2 | 6.0E-01 | **8.8** | **3.4E-04** | 14.9 | 3.9E-06 |
| CCL2 | -4.0 | 2.7E-01 | -9.1 | 6.3E-02 | **-10.3** | **2.5E-02** |
| KRT86 | -1.5 | 2.6E-01 | -4.5 | 8.6E-02 | **-5.3** | **1.3E-08** |
| IGHA1 | -2.1 | 8.2E-01 | -4.5 | 1.6E-01 | **-4.7** | **3.9E-02** |
| THSD7A | -1.1 | 4.9E-01 | -1.9 | 3.2E-02 | **-3.9** | **7.8E-07** |
| IGLV10-54 | -1.2 | 4.2E-01 | -2.8 | 8.5E-01 | **-3.8** | **9.5E-03** |
| FTCD | -2.2 | 5.3E-02 | -3.0 | 8.6E-02 | **-3.2** | **3.1E-02** |
| CNN1 | -1.2 | 5.5E-01 | -2.1 | 3.3E-01 | **-3.1** | **4.1E-02** |
| IGKV4-1 | -1.2 | 9.5E-01 | -2.9 | 5.5E-02 | **-2.8** | **1.0E-02** |
| ANKUB1 | -1.9 | 1.5E-01 | -2.5 | 5.6E-02 | **-2.6** | **4.0E-05** |
| NTRK1 | -1.1 | 9.2E-02 | -1.3 | 1.2E-01 | **-2.4** | **1.1E-08** |
| ENTPD2 | -1.4 | 2.6E-01 | -1.9 | 1.3E-01 | **-2.3** | **8.4E-03** |
| SCN4A | -1.9 | 3.8E-01 | -1.9 | 4.5E-01 | **-2.3** | **2.5E-02** |
| CACNB4 | -1.6 | 5.6E-02 | -1.7 | 1.5E-01 | **-2.2** | **1.6E-03** |
| CAV2 | -1.2 | 4.3E-01 | -1.9 | 9.8E-03 | **-2.2** | **4.5E-04** |
| CDR2L | -1.1 | 7.1E-01 | -1.8 | 8.6E-02 | **-2.2** | **2.0E-02** |
| APCDD1 | -1.4 | 2.8E-01 | -1.9 | 7.0E-02 | **-2.1** | **8.5E-03** |
| DHRS9 | -1.8 | 2.6E-01 | -2.2 | 7.4E-02 | **-2.1** | **8.8E-03** |
| SEMA3G | -1.8 | 2.5E-01 | -2.2 | 8.5E-02 | **-2.1** | **4.1E-02** |
| SNAI1 | -1.3 | 9.7E-02 | -1.5 | 2.8E-01 | **-2.1** | **1.1E-02** |
| TRGJP | -1.3 | 2.1E-01 | -1.7 | 3.7E-02 | **-2.1** | **2.0E-03** |
| TTLL7 | -1.1 | 2.9E-01 | -1.9 | 7.9E-03 | **-2.1** | **4.5E-03** |
| CCR9 | -1.1 | 9.2E-01 | -1.2 | 9.9E-01 | **-2.0** | **1.1E-02** |
| GCSAML | 1.0 | 4.8E-01 | -1.4 | 5.9E-03 | **-2.0** | **1.4E-04** |
| IGHV2-5 | -1.1 | 9.7E-01 | -1.7 | 4.7E-01 | **-2.0** | **2.8E-02** |
| IGHV3-15 | -1.0 | 8.1E-01 | -1.5 | 5.1E-01 | **-2.0** | **4.9E-02** |
| IGLV3-25 | -1.2 | 8.1E-01 | -2.0 | 1.5E-01 | **-2.0** | **1.6E-02** |
| MT-ND6 | -1.2 | 8.9E-01 | -1.7 | 5.5E-01 | **-2.0** | **3.9E-02** |
| NUAK1 | -1.4 | 5.8E-01 | -1.7 | 2.4E-01 | **-2.0** | **1.6E-02** |
| RTKN | -1.4 | 4.4E-01 | -1.8 | 1.9E-01 | **-2.0** | **3.6E-03** |
| ANXA3 | 1.4 | 3.0E-06 | 1.7 | 2.3E-08 | **2.0** | **4.4E-12** |
| DACT1 | 1.1 | 6.5E-01 | 1.6 | 2.3E-01 | **2.0** | **3.8E-02** |
| DSC2 | 1.3 | 4.1E-03 | 1.9 | 2.5E-04 | **2.0** | **1.8E-06** |
| GPR84 | 1.2 | 7.3E-01 | 1.6 | 3.1E-02 | **2.0** | **2.6E-03** |
| MS4A3 | -1.2 | 9.2E-01 | 1.7 | 1.9E-01 | **2.0** | **2.0E-02** |
| RPS21 | 1.4 | 2.8E-01 | 2.0 | 7.0E-02 | **2.0** | **2.9E-02** |
| ANKRD35 | 1.2 | 6.6E-01 | 1.8 | 3.3E-01 | **2.1** | **1.1E-02** |
| CD24 | -1.1 | 7.0E-01 | 1.7 | 8.4E-03 | **2.1** | **3.0E-05** |
| CDCA2 | 1.8 | 2.2E-01 | 1.7 | 1.2E-01 | **2.1** | **1.7E-02** |
| MPO | -1.2 | 6.3E-01 | 1.5 | 1.0E-01 | **2.1** | **3.7E-04** |
| RPS18 | 1.4 | 2.0E-01 | 2.0 | 6.8E-02 | **2.1** | **2.0E-02** |
| SLC2A5 | 1.1 | 6.8E-01 | 1.8 | 4.7E-02 | **2.1** | **1.5E-03** |
| SLPI | 1.4 | 9.2E-02 | 1.9 | 9.4E-05 | **2.1** | **3.3E-08** |
| TARM1 | -2.0 | 8.5E-01 | 1.3 | 1.2E-01 | **2.1** | **8.2E-03** |
| CHIT1 | 1.2 | 3.4E-01 | 1.8 | 2.5E-03 | **2.2** | **5.0E-06** |
| EEF1B2 | 1.6 | 2.3E-01 | 2.1 | 1.3E-01 | **2.2** | **4.1E-02** |
| GPRC5B | 1.0 | 8.7E-01 | 1.6 | 2.9E-01 | **2.2** | **1.6E-02** |
| PGLYRP1 | 1.4 | 2.5E-02 | 1.8 | 9.8E-03 | **2.2** | **9.1E-07** |
| ANOS1 | 1.1 | 9.7E-03 | 1.5 | 4.3E-05 | **2.3** | **1.4E-09** |
| AZU1 | -1.5 | 9.6E-01 | 1.4 | 3.0E-02 | **2.3** | **2.3E-04** |
| CYYR1 | 1.3 | 6.4E-01 | 2.0 | 1.9E-01 | **2.3** | **3.2E-02** |
| ELOVL3 | 1.1 | 8.2E-01 | 2.1 | 1.2E-01 | **2.3** | **2.1E-02** |
| HPDL | 2.4 | 2.0E-01 | 2.5 | 5.1E-02 | **2.3** | **8.3E-03** |
| LPL | 1.2 | 1.8E-01 | 1.4 | 2.4E-01 | **2.4** | **2.6E-02** |
| LRRIQ3 | 1.6 | 3.4E-01 | 2.9 | 7.5E-02 | **2.5** | **3.8E-02** |
| ORM1 | 1.2 | 7.8E-01 | 2.1 | 2.9E-01 | **2.5** | **4.4E-02** |
| CTSG | -1.8 | 5.0E-01 | 1.8 | 5.9E-02 | **2.7** | **2.5E-04** |
| HEMGN | 1.2 | 8.5E-01 | 2.4 | 1.1E-01 | **2.7** | **1.8E-02** |
| NDST3 | 1.7 | 2.2E-01 | 2.0 | 6.1E-02 | **2.7** | **9.9E-03** |
| ORM2 | 1.2 | 8.6E-01 | 1.9 | 8.6E-02 | **2.7** | **6.9E-04** |
| INSC | 2.2 | 5.2E-02 | 2.4 | 6.3E-02 | **2.8** | **7.8E-03** |
| PAPPA2 | 1.8 | 1.1E-01 | 2.5 | 1.5E-01 | **2.8** | **4.1E-05** |
| RPL7 | 1.8 | 1.3E-01 | 2.6 | 1.3E-01 | **2.8** | **3.6E-02** |
| SLC51A | 1.1 | 5.4E-01 | 1.9 | 3.2E-02 | **2.8** | **4.7E-05** |
| TCN1 | -1.2 | 7.8E-01 | 1.9 | 2.3E-03 | **2.8** | **4.1E-07** |
| TFF3 | -1.0 | 8.8E-01 | 2.5 | 2.2E-01 | **2.8** | **3.8E-02** |
| GYPB | 1.4 | 8.3E-01 | 2.9 | 1.1E-01 | **2.9** | **7.9E-03** |
| PRRT4 | 1.2 | 7.0E-01 | 2.0 | 2.0E-01 | **2.9** | **3.5E-03** |
| TWIST2 | 1.4 | 3.9E-01 | 3.2 | 9.2E-02 | **2.9** | **2.9E-02** |
| XK | 1.1 | 3.5E-01 | 2.4 | 5.0E-01 | **3.2** | **1.7E-02** |
| BCAM | 1.2 | 6.5E-01 | 3.4 | 7.1E-01 | **4.7** | **3.4E-02** |
| PCOLCE2 | -1.3 | 8.6E-01 | 2.2 | 1.5E-01 | **5.2** | **2.0E-03** |
