# Supplementary material for: Pregnancy-associated systemic gene expression compared to a pre-pregnancy baseline, among healthy women with term pregnancies
Source: Front Immunol. 2023 Jun 5;14:1161084. doi: 10.3389/fimmu.2023.1161084 (PMC10277629; doi:10.3389/fimmu.2023.1161084)
Supplement: Supplementary file 5 [file Table_3.docx]

**Table S3. Protein-coding genes and lncRNAs co-expressed within WGCNA modules, together with GO terms associated with these modules.**

In the Weighted Gene Co-expression Network Analysis (WGCNA) of all genes that were analyzed in the GEE models, there were several modules with protein-coding genes and lncRNA co-expression and/or with pregnancy-associated gene enrichment. The specific pregnancy-associated lncRNAs and/or genes co-expressed within each of these modules, together with the GO terms enriched in these genes, are shown here. There were no GO terms enriched in genes that were in the darkred, darkgrey, green, darkturquoise, magenta, and black modules.

| **WGCNA module name** | **Long non-coding RNAs** | **Protein coding genes** | **Gene Ontology (GO) terms** |
| --- | --- | --- | --- |
| darkred | AL645608.7, MSTRG.13724 | IDO1, IL34, RASAL2 |  |
| darkgrey | LINC02458, AL356489.2, DLGAP1-AS3, PTENP1-AS | GATA2, IL4, THSD7A, PTGER3, ITGB8, CAV2, SNAI1, CACNG6, AKAP12, TTLL7, HDC, MS4A2, ENPP3, SLC45A3, SCUBE1, FTCD, CPA3, C10orf82, GCSAML, PHYHD1, FCER1A, KRT5, SLC2A10, NTRK1, RYR3 |  |
| midnightblue | AC034238.1 | TRGJP | Natural killer cell mediated immunity (FDR=0.0009)  Cellular defense response (FDR=0.001)  Lymphocyte mediated immunity (FDR=0.002)  Regulation of immune response (FDR=0.03)  Natural killer cell activation (FDR=0.04) |
| blue | LINC00200, CSTF3-DT, AP000265.1, MSTRG.36453 | NUAK1, DCT, RTKN, CCR9, IGLV1-47, IGHV2-5, SIAH3 | Cellular amino acid metabolic process (FDR=6.7E-07)  Carboxylic acid metabolic process (FDR=7.9E-05)  tRNA metabolic process (FDR=0.0003)  Oxoacid metabolic process (FDR=0.0003)  Organic acid metabolic process (FDR=0.0003)  Alpha-amino acid metabolic process (FDR=0.007)  ncRNA metabolic process (FDR=0.008)  tRNA aminoacylation (FDR=0.009)  Amino acid activation (FDR=0.01)  Neuromuscular process controlling posture (FDR=0.02) |
| green | MIR5189, AC008079.1 | ENTPD2, SHFL, RPL39L, WNK2, KIF7, NLRP7, AGRN |  |
| darkturquoise |  | IGKV4-1, IGLV10-54, IGLV3-25, IGHA1, IGHV3-15, IGLV1-40, IGHV3-15, IGHV1-46 |  |
| yellow | MIR644A, AC022079.1 | PSMA8 | N-terminal peptidyl-methionine acetylation (FDR=0.0264)  ncRNA processing (FDR=0.03)  Regulation of DNA damage checkpoint (FDR=0.03)  Regulation of cell cycle checkpoint (FDR=0.04) |
| magenta | MIR591, RABGAP1L-IT1, PKN2-AS1, MIR4451 | KIAA1958, RNF213, OR52K2 |  |
| darkgreen | AC131011.1 | SIGLEC1, OAS1, OAS2, OAS3, OASL, IFI6, IFI44, IFI44L, IFIH1, IFIT1, IFIT3, MX1, STAT2, RSAD2, CMPK2, HERC5, HERC6, SERPING1, KPTN, MT2A, XAF1, EPSTI1, RTP4, TOR1B, DDX60, PNPT1, PML, ZCCHC2, MOV10, FBXO39, PARP10, USP18, ISG15, PARP12, DHX58, DHRS9, LAMP3 | Response to biotic stimulus (FDR<2.2E-16)  Innate immune response (FDR<2.2E-16)  Defense response to other organism (FDR=0)  Response to virus (FDR<2.2E-16)  Response to type I interferon (FDR<2.2E-16)  Type I interferon signaling pathway (FDR<2.2E-16) |
| lightyellow |  | RPL31, RPL34, CKS2, RPL23, COX7C, TPT1, RPS3A, POLR2K, RPL9, RPS7, RPS27, RPL39, SMIM30, RPL41, RPL7 | SRP-dependent co-translational protein targeting to membrane (FDR<2.2E-16)  mRNA catabolic process (FDR=1.12E-09)  Translation (FDR=8.51E-09)  Proton transmembrane transport (FDR=1.2E-06)  ATP metabolic process (FDR=1.8E-06)  Purine ribonucleotide metabolic process (FDR=0.0002)  Mitochondrial ATP synthesis coupled proton transport (FDR=0.0002)  Mitochondrial ATP synthesis coupled electron transport (FDR=0.02) |
| purple | AL358072.1 | LRRIQ3 | RNA processing (FDR=7.7E-08)  Ribonucleoprotein complex biogenesis (FDR=0.0006)  RNA localization (FDR=0.0008)  RNA splicing (FDR=0.0008)  ncRNA metabolic process (FDR=0.0008)  Ribosome biogenesis (FDR=0.0008)  ncRNA processing (FDR=0.001)  mRNA splicing, via spliceosome (FDR=0.001) |
| black | AC079305.1, AL135960.1, AP001610.2, AP006621.1, AL139022.1, AC010761.1, AC011498.6, AC006547.3, AC005387.1, ABALON, MSTRG.23857, MSTRG.3367, MSTRG.38112 | MAP1LC3A, UPK2, WNT6, SCUBE3, TMEM262 |  |
| orange | LINC00900, LINC02009 | OLFM4, CAMP, DEFA4, ELANE, DEFA1, DEFA3, CD24, MMP8, CEACAM6, CEACAM8, CRISP3, MPO, LTF, ATP2C2, COL17A1, CTSG, BPI, RETN, INHBA, TCN1, SLC2A5, LCN2, MS4A3, ERG, TFF3, RNASE3, HTRA3, AZU1, STOX2, OLR1, ABCA13, IQGAP3, PRTN3, SERPINB10, TARM1, PRRT4 | Exocytosis (FDR<2.2E-16)  Leukocyte mediated immunity (FDR<2.2E-16)  Cell activation involved in immune response (FDR=0)  Myeloid leukocyte activation (FDR<2.2E-16)  Leukocyte degranulation (FDR<2.2E-16)  Granulocyte activation (FDR<2.2E-16) |
| red | CCR5AS, LINC00853, NRIR, AC078802.1 | XK, HEMGN, CELF3, IGF2, LRRN2, BCAM, GYPB | Mitochondrion disassembly (FDR=3.3E-06)  Cellular protein catabolic process (FDR=3.3E-06)  Organelle disassembly (FDR=1.2E-05)  Proteolysis involved in cellular protein catabolic process (FDR=2.4E-05)  Cellular component disassembly (FDR=3.2E-05)  Protein ubiquitination (FDR=9.0E-05) |
| turquoise | BASP1-AS1, LINC01907, LINC02818, CLRN1-AS1, AP003086.1, AC073172.1, AP001063.1, AC011524.1, AC011524.2, AC087645.2, AC073342.2, AL035661.1, AC009301.1, AC132938.6, AC006511.6, MSTRG.23626, MSTRG.24196, MSTRG.28602, MSTRG.33153, MSTRG.6504, MSTRG.7338 | PGLYRP1, S100A8, S100A9, S100A12, S100P, ARG1, CD177, ORM2, ORM1, ANOS1, EIF2AK2, PAPPA2, SLPI, EVI2A, OMG, HELZ2, ITGB4, CHIT1, DSC2, ANXA3, GPR84, BCL2A1, GALNT14, SHROOM4, CAPN13, INHBB, STPG2, PCOLCE2, ZDHHC19, SLC51A, NDST3, ABTB2, CYYR1, SEMA6B, RSPH9, INSC, HSH2D, SRGAP1, TWIST2, CBSL | Response to cytokine (FDR<2.2E-16)  Regulation of immune response (FDR<2.2E-16)  Exocytosis (FDR<2.2E-16)  Leukocyte mediated immunity (FDR<2.2E-16)  Innate immune response (FDR<2.2E-16) |
| grey | LINC00469, LINC01597, MIR25, MIR589, AC087071.1, LINC01765, TPRG1-AS1, AC007879.3, AC090952.1, TUSC8, LINC01252, AC079848.1, AC027117.1, AL355432.1, LINC02367, AL138787.2, AL021807.1, AC006238.1, AC093458.1, AC006946.3, AL445584.2, AC092902.4, AC132153.1, AC023283.1, MSTRG.2080, MSTRG.684 | SCN4A, SEMA3G, RAPGEF3, SH2D4A, CNFN, CCL2, MDK, HHLA2, OTOF, IRF6, ELOVL3, CCNA1, ADAMTS14, APCDD1, LY6E, GRIK3, DACT1, GPRC5B, TTC21A, KRT86, LPL, UBQLN3, TMEM132C, CACNB4, FAM167B, HPDL, RFX8, ANKRD35, ANKUB1, TRMT9B, OR13A1, AC005520.1, IGHV2-70, IGHV3-64D, AC090517.4 | Sensory perception (FDR=3.3E-06)  G protein-coupled receptor signaling pathway (FDR=3.3E-06)  Nervous system process (FDR=3.3E-06)  Neuron differentiation (FDR=8.6E-06)  Detection of stimulus involved in sensory perception (FDR=1.03E-05)  Detection of chemical stimulus involved in sensory perception of smell (FDR=2.4E-05)  Regulation of neurogenesis (FDR=2.4E-04) |
