# Supplementary material for: Pregnancy-associated systemic gene expression compared to a pre-pregnancy baseline, among healthy women with term pregnancies
Source: Front Immunol. 2023 Jun 5;14:1161084. doi: 10.3389/fimmu.2023.1161084 (PMC10277629; doi:10.3389/fimmu.2023.1161084)
Supplement: Supplementary file 6 [file Table_4.docx]

**Table S4. Transcription factors and their target genes whose expression patterns were associated with different stages of pregnancy.**

Several of the genes whose expression were associated with one or more trimesters of pregnancy or with the postpartum time-point (in the GEE model adjusted for changes in cell type proportion) were identified as target genes for transcription factors (TFs). Only TFs that had at least 2 target genes showing pregnancy-associated expression at or from a specific time-point are shown.

| **Transcription factor (TF)** | **TF gene targets** |
| --- | --- |
| *Genes whose expression were under transcriptional regulation at/from T1 (n=26)* | |
| STAT2 | CMPK2, EPSTI1, OAS2, OAS3 |
| IRF9 | OAS2, OAS3, RSAD2 |
| STAT1 | IDO1, OAS2, OAS3, RSAD2 |
| E2F1 | ABTB2, CCNA1 |
| FOS | ABTB2, AGRN |
| EGR1 | AGRN, LY6E |
|  |  |
| *Genes whose expression were under transcriptional regulation at/from T2 (n=136)* | |
| STAT2 | DDX60, DHX58, EIF2AK2, IFI6, IFIH1, IFIT1, IFIT3, ISG15, MOV10, MX1, OAS1, OASL, PARP10, PARP12, PML, PNPT1, RNF213, RTP4, XAF1 |
| IRF9 | IFIT1, IFIT3, ISG15, MX1, PML |
| STAT1 | ABCA13, EIF2AK2, IFI6, IFIT1, IFIT3, ISG15, MX1, OAS1, OASL, PML, RNF213, SHFL, STAT2, ZCCHC2 |
| CEBPA | ATP2C2, BCL2A1, DEFA3, ELANE, ENPP3, ERG, IL4, LTF, OLR1, S100A9, WNK2 |
| CTCF | ABCA13, GALNT14, GRIK3, OTOF, PARP10, RAPGEF3, RPL39L, RYR3, SEMA6B, WNK2 |
| SPI1 | BCL2A1, BPI, ELANE, ERG, FCER1A, IFIT3, MMP8, PRTN3 |
| TFAP2C | ATP2C2, GALNT14, GATA2, GRIK3, HSH2D, PARP10, WNK2 |
| EGR1 | AKAP12, ERG, GATA2, MOV10, SRGAP1, TMEM132C, ZCCHC2 |
| HNF4A | RPS3A, RPS7, SHFL, WNK2, ZCCHC2 |
| IRF1 | EIF2AK2, IFIT3, IL4, ISG15 |
| RUNX1 | ERG, BPI, HHLA2, RPS3A |
| MYC | CKS2, HERC5, PRTN3, RPL23 |
| GATA2 | ERG, FCER1A, MS4A2, RASAL2 |
| ESR1 | ERG, LTF, PML, WNK2 |
| MITF | BCL2A1, DCT, ERG, GRIK3 |
| GATA3 | ERG, IL4, PTGER3, RASAL2 |
| FOXA1 | ERG, GALNT14, OTOF, RASAL2 |
| ETS1 | ERG, KIAA0895L, LTF, RNF213 |
| IRF2 | IFIT3, IL4, ISG15 |
| TFAP2A | ATP2C2, ITGB4, KRT5 |
| NFKB1 | BCL2A1, IL4, OLR1 |
| SOX2 | ABCA13, LTF, RYR3 |
| SP1 | KRT5, LTF, RPS3A |
| ZNF263 | ERG, SRGAP1, WNK2 |
| TAL1 | ERG, LTF, RYR3 |
| TP53 | PML, RPL39L, ZCCHC2 |
| FOS | ABCA13, MAP1LC3A, SRGAP1 |
| PRDM1 | ERG, OLR1 |
| LEF1 | DCT, ELANE |
| POU2F1 | IL4, MS4A2 |
| MYB | ELANE, GATA2 |
| CEBPB | IL4, S100A9 |
| USF1 | CEACAM6, FCER1A |
| ZEB1 | HELZ2, ITGB4 |
| USF2 | CEACAM6, FCER1A |
| RELA | BCL2A1, IL4 |
| VDR | CAMP, SH2D4A |
| PRDM14 | GATA2, INHBB |
| E2F1 | MAP1LC3A, TOR1B |
| E2F4 | CKS2, IQGAP3 |
|  |  |
| *Genes whose expression were under transcriptional regulation at/from T3 (n=71)* | |
| ESR1 | CAV2, CD24, DHRS9, KRT86 |
| NFKB1 | CCL2, LPL, TFF3 |
| HIF1A | CCL2, NTRK1, TFF3 |
| SPI1 | CTSG, DHRS9, MPO |
| FOXA1 | CAV2, PAPPA2, THSD7A |
| FOS | ANXA3, CCL2, CDCA2 |
| STAT1 | CCL2, PAPPA2 |
| GATA2 | SNAI1, THSD7A |
| JUN | CCL2, NTRK1 |
| AR | ORM1, ORM2 |
| RELA | CCL2, SNAI1 |
| PRDM14 | ANXA3, NTRK1 |
| SP1 | CCL2, MPO |
| ZNF263 | FTCD, NTRK1 |
| E2F4 | CDCA2, NTRK1 |
| HNF4A | CHIT1, DSC2 |
| TFAP2C | APCDD1, GPRC5B |
| CTCF | CACNB4, CHIT1 |
